# Supplementary material for: Using clinical cascades to measure health facilities’ obstetric emergency readiness: testing the cascade model using cross-sectional facility data in East Africa
Source: BMJ Open. 2022 Apr 3;12(4):e057954. doi: 10.1136/bmjopen-2021-057954 (PMC8981352; doi:10.1136/bmjopen-2021-057954)
Supplement: Supplementary data [file bmjopen-2021-057954supp001.pdf]

SUPPLEMENTAL MATERIAL

Definition of Terms

- **Maternal deaths:** The death of a woman during pregnancy or within 42 days of termination of the pregnancy, regardless of the duration and site of the pregnancy, from any cause related to or exacerbated by the pregnancy or its management, excluding accidental or incidental causes[1]
- **Live birth:** The complete expulsion or extraction of a product of conception from its mother, irrespective of pregnancy duration, which, after separation, breathes or shows other signs of life[1]
- **Maternal Mortality Ratio (MMR):** Maternal deaths per 100,000 live births[1]
- **Hemorrhage:** Severe bleeding before (antepartum hemorrhage) or after (postpartum hemorrhage) labor[2]
- **Sepsis:** A complication caused by infection, resulting in high temperatures (38° C or higher) and one or more of the following symptoms: lower abdominal pain, abnormal vaginal discharge, and tender or poorly contracted uterus[2]
- **Preeclampsia:** A sudden spike in maternal blood pressure accompanied by protein in the urine, which can progress to a more serious eclampsia[2]
- **Eclampsia:** A blood-pressure related complication that can result in maternal seizures which, untreated, can progress to coma or death[2]
- **Prolonged or obstructed labor:** Occurs when the first stage of labor lasts longer than 12 hours, the second stage of labor lasts longer than one hour, or labor is obstructed by uterine scarring or fetal malpresentation (fetus side (transverse), brow, or face presenting in the uterine isthmus instead of the head)[2]
- **Abortion:** The termination of a pregnancy through the removal of pregnancy tissue, products of conception, or the fetus and placenta from the uterus[3]. Complications of a spontaneous or induced abortion, especially an induced abortion performed in non-medical/unsterile settings, can result in hemorrhage, sepsis, or other complications[2]
- **Signal functions:** Six clinical actions, three medical and three manual procedures, used during obstetric emergencies[2]
- **Clinical cascades:** A clinically-oriented approach to measuring facility readiness using a step-wise cascading relationship between emergency resources[4,5]
- **Emergency Obstetric Care (EmOC):** Clinical processes needed to rapidly manage or stabilize a patient whose life is in imminent danger during pregnancy[6]
- **Basic Emergency Obstetric Care (BEmOC):** The ability to perform six maternal signal functions: (1) administer parenteral antibiotics, (2) administer uterotonic drugs, (3) administer parenteral anticonvulsants for pre-eclampsia and eclampsia, (4) manually remove the placenta, (5) remove retained products of conception, and (6) perform assisted vaginal delivery[2]
- **Comprehensive Emergency Obstetric Care (CEmOC):** The ability to perform the six basic signal functions as well as surgery and blood transfusion[2]
- **Drop-off in readiness:** The decrease in the percentage of facilities with the resources necessary to manage basic obstetric emergencies.
- **Private facilities:** Facilities across both Kenya and Uganda that are neither owned nor operated by the government.

Table S1: Items that Define Signal Functions and Clinical Cascades

**Obstetric Emergency**	**Signal Function**	**Signal Function Tracer Items**	**Clinical Cascade Items by Stage of Care**		
			Stage 1: Identify	Stage 2: Treat	Stage 3: Monitor-Modify
Manage Sepsis-Infection	Administer Parenteral Antibiotics	IV Fluid (1)<br>IV Cannula/Catheter<br>IV Tubing<br>Ampicillin or Penicillin<br>Gentamicin or Cefotaxime or Ceftriaxone	Thermometer<br>Stethoscope<br>Sphygmomanometer	IV Tubing<br>IV Cannula/Catheter<br>IV Fluid (1)<br>IV Pole<br>Ampicillin or Penicillin<br>Gentamicin or Cefotaxime or Ceftriaxone	Protocol: Sepsis (2)
Manage Hemorrhage	Administer Parenteral Uterotonics	Gloves (3)<br>IV Fluid (1)<br>IV Cannula/Catheter<br>IV Tubing<br>Oxytocin or Misoprostol or Ergometrine	Staff skill (4)	Gloves (3)<br>IV Tubing<br>IV Cannula/Catheter<br>IV Fluid (1)<br>IV Pole<br>Refrigeration (5)<br>Oxytocin	Sphygmomanometer<br>Stethoscope<br>Misoprostol or Ergometrine<br>Urinary Catheter<br>Oxygen Source (6)<br>Oxygen Tubing<br>Oxygen Delivery (7)<br>Protocol: Hemorrhage
Manage Hypertensive Emergencies	Administer Parenteral Anticonvulsant	IV Fluid (1)<br>IV Cannula/Catheter<br>IV Tubing	Sphygmomanometer<br>Stethoscope<br>Urine Cup	IV Tubing<br>IV Cannula/Catheter<br>IV Fluid (1)	Urinary Catheter<br>Calcium Gluconate<br>Oxygen Source (6)

		Magnesium Sulfate	Urine Dipstick	IV Pole<br>Magnesium Sulfate<br>Hydralazine or Labetalol or Nifedipine or Methyldopa	Oxygen Tubing<br>Oxygen Delivery [7]<br>Protocol: Eclampsia
Manage Retained Placenta	Perform Manual Removal of Retained Placenta	Flashlight or power<br>IV Fluid [1]<br>IV Cannula/Catheter<br>IV Tubing<br>Oxytocin<br>Ampicillin or Penicillin or Gentamicin or Cefotaxime or Ceftriaxone	Staff Skill [4]<br>Light Source [8]	Gloves [3]<br>IV Tubing<br>IV Cannula/Catheter<br>IV Fluid [1]<br>IV Pole<br>Refrigeration [5]<br>Oxytocin<br>Diazepam<br>Ampicillin or Penicillin	Sphygmomanometer<br>Stethoscope<br>Misoprostol or Ergometrine<br>Gentamicin or Cefotaxime or Ceftriaxone<br>Urinary Catheter<br>Protocol(s) [9]
Manage Incomplete Abortion	Perform Manual Removal of Retained Products of Conception	Flashlight or power<br>MVA Kit<br>IV Fluid [1]<br>IV Cannula/Catheter<br>IV Tubing	Speculum<br>Light Source [8]	Gloves [3]<br>IV Tubing<br>IV Cannula/Catheter<br>IV Fluid [1]<br>Manual Vacuum Aspirator Kit [10]	Sphygmomanometer<br>Stethoscope<br>Refrigeration [5]<br>Misoprostol or Ergometrine<br>Gentamicin or Cefotaxime or Ceftriaxone

		Ampicillin or Penicillin or Gentamicin or Cefotaxime or Ceftriaxone		IV Pole<br><br>Lidocaine<br>Ampicillin or Penicillin	Urinary Catheter<br><br>Protocol(s) [(11)]

[(1)] Either normal saline (NS) or lactated ringer's (LR)

[(2)] Puerperal sepsis, infection, or antibiotic administration Protocol

[(3)] Sterile gloves or clean disposable latex gloves (represented as a single variable in the original dataset)

[(4)] 100% staff skill for identifying the emergency was assumed

[(5)] Power used as proxy for refrigeration

[(6)] Oxygen concentrator and power or oxygen cylinder and oxygen head

[(7)] Oxygen mask or nasal cannula

[(8)] Flashlight or power (as proxy for electric lights)

[(9)] Retained placenta protocol or hemorrhage protocol or infection protocol

[(10)] Manual vacuum device and cannula

[(11)] Incomplete abortion protocol or hemorrhage protocol or infection protocol

Table S2. Facility Characteristics

	**%**	**n** [1]
**Country**		
Kenya	73.9%	17
Uganda	26.1%	6
**Level (ownership)**		
Private	17.4%	4
Government	82.6%	19
Regional Referral Hospital	*4.4%*	*1*
District Hospital	*13.0%*	*3*
County Referral Hospital	*4.4%*	*1*
Sub-County Hospital	*43.5%*	*10*
Health Center	*17.4%*	*4*
**Reported C-Section Capability**		
Yes	43.5%	10
No	56.5%	13

[1] n = 23

Table S3. Annual Delivery Volume

	n	Annual Total [(1)]	Median [(2)]	25th	75th	IQR
**Kenya**	17	15,858.5	598.9	495.3	999.3	504.0
**Uganda**	6	21,591.3	2,662.5	2,059.3	5,396.0	3,336.7
Total	23	*37,449.8*	*994.5*	*537.3*	*1,808.6*	*1,271.4*

[(1)] Data was provided for up to 11 months in Kenya and up to 12 months in Uganda. To calculate the total annual delivery volume, we calculate the monthly average using the number of reported months for each individual facility and then multiplied that number by 12.
[(2)] The median and IQR are reported instead of the mean and standard deviation as the data are not normally distributed.

Table S4. Comparison of Emergency Readiness Using Clinical Cascades and Signal Functions by Reported C-Section Capability

	**Signal Functions**		**Clinical Cascades**		**Overestimated Readiness**	
	**% Readiness, Tracer Items**		**% Readiness, Stage 2**		**Percentage Point Difference [Signal Functions (-) Cascade]**	
**Clinical Cascade** *(Signal Function)*	**C-section** (1)	**No C-section** (2)	**C-section**	**No C-section**	**C-section**	**No C-section**
**Medical Treatments**						
**Manage Sepsis - Infection** *(Antibiotic)*	90.0%	53.9%	50.0%	46.2%	40.0	7.7
**Manage Hemorrhage** *(Uterotonics)*	70.0%	53.9%	70.0%	53.9%	0.0	0.0
**Manage Hypertensive Emergency** *(Anticonvulsant)*	90.0%	69.2%	30.0%	23.1%	60.0	46.2
Medical Readiness, Overall Mean (pooled)	83.3%	59.0%	50.0%	41.0%	33.3	18.0
**Manual Procedures**						
**Manage Retained Placenta** *(Removal of retained placenta)*	70.0%	69.2%	70.0%	23.1%	0.0	46.2
**Manage Incomplete Abortion** *(Removal of retained products of conception)*	80.0%	61.5%	70.0%	46.2%	10.0	15.4
Manual Readiness, Overall Mean (pooled)	75.0%	65.4%	70.0%	34.6%	5.0	30.8
**Overall Mean Readiness (pooled)**	**80.0%**	**61.5%**	**58.0%**	**38.5%**	**22.0**	**23.1**
	Signal Function Estimate		*Cascade Estimate*		*Percentage Point Overestimation by Signal Functions*	

(1) n = 10 facilities
(2) n = 13 facilities

Table S5. Comparison of Emergency Readiness Using Clinical Cascades and Signal Functions by Ownership

	**Signal Functions**		**Clinical Cascades**		**Overestimated Readiness**	
	**% Readiness, Tracer Items**		**% Readiness, Stage 2**		**Percentage Point Difference [Signal Functions (-) Cascade]**	
**Clinical Cascade** *(Signal Function)*	**Gov.** [1]	**Private** [2]	**Gov.**	**Private**	**Government**	**Private**
**Medical Treatments**						
**Manage Sepsis - Infection** *(Antibiotic)*	63.2%	100.0%	42.1%	75.0%	21.1	25.0
**Manage Hemorrhage** *(Uterotonics)*	57.9%	100.0%	57.9%	100.0%	0.0	0.0
**Manage Hypertensive Emergency** *(Anticonvulsant)*	73.7%	100.0%	26.3%	25.0%	47.4	75.0
Medical Readiness, Overall Mean (pooled)	64.9%	100.0%	42.1%	66.7%	22.8	33.3
**Manual Procedures**						
**Manage Retained Placenta** *(Removal of retained placenta)*	68.4%	100.0%	36.8%	100.0%	31.6	0.0
**Manage Incomplete Abortion** *(Removal of retained products of conception)*	63.2%	75.0%	52.6%	75.0%	10.5	0.0
Manual Readiness, Overall Mean (pooled)	65.8%	87.5%	44.7%	87.5%	21.1	0.0
**Overall Mean Readiness (pooled)**	**65.3%**	**95.0%**	**43.2%**	**75.0%**	**22.1**	**20.0**
	Signal Function Estimate		*Cascade Estimate*		*Percentage Point Overestimation by Signal Functions*	

[1] n = 19 facilities
[2] n = 4 facilities

Table S6. Comparison of Emergency Readiness Using Clinical Cascades and Signal Functions by Country

	**Signal Functions**		**Clinical Cascades**		**Overestimated Readiness**	
	**% Readiness, Tracer Items**		**% Readiness, Stage 2**		**Percentage Point Difference[Signal Functions (-) Cascade]**	
**Clinical Cascade** *(Signal Function)*	**Kenya**[1]	**Uganda**[2]	**Kenya**	**Uganda**	**Kenya**	**Uganda**
**Medical Treatments**						
**Manage Sepsis – Infection** *(Antibiotic)*	64.7%	83.3%	52.9%	33.3%	11.8	50.0
**Manage Hemorrhage** *(Uterotonics)*	58.8%	66.7%	58.8%	66.7%	0.0	0.0
**Manage Hypertensive Emergency** *(Anticonvulsant)*	76.5%	83.3%	23.5%	33.3%	52.9	50.0
Medical Readiness, Overall Mean (pooled)	66.7%	77.8%	45.1%	44.4%	21.6	33.3
**Manual Procedures**						
**Manage Retained Placenta** *(Removal of retained placenta)*	70.6%	66.7%	35.3%	66.7%	35.3	0.0
**Manage Incomplete Abortion** *(Removal of retained products of conception)*	70.6%	66.7%	58.8%	50.0%	11.8	16.7
Manual Readiness, Overall Mean (pooled0	70.6%	66.7%	47.0%	58.3%	23.5	8.3
**Overall Mean Readiness (pooled)**	**68.2%**	**73.3%**	**45.9%**	**50.0%**	**22.4**	**23.3**
	Signal Function Estimate		*Cascade Estimate*		*Percentage Point Overestimation by Signal Functions*	

(1) n = 17 facilities
(2) n = 6 facilities

Table S7. Emergency Readiness by Clinical Cascade

**Clinical Cascade** ***(Signal Function)***	**Cascade Stage**	**Item**	**%**	**n [1]**
Manage Sepsis-Infection *(Antibiotic)*	Identify	Thermometer	95.7	22
		Stethoscope	78.3	18
		Sphygmomanometer	73.9	17
	Treat (Consumables)	IV Tubing	60.9	14
		IV Cannula-Catheter	52.2	12
		IV Fluid [2]	52.2	12
	Treat (Durables)	IV Pole	52.2	12
	Treat (Treatments)	Parenteral Antibiotic-Step 1 [3]	52.2	12
		Parenteral Antibiotic-Step 2 [4]	47.8	7
	Monitor-Modify	Protocol: Sepsis [5]	30.4	1
Manage Hemorrhage (*Uterotonic*)	Identify	Staff skill [6]	100.0	23
	Treat (Consumables)	Gloves, aseptic [7]	78.3	18
		IV Tubing	69.6	16
		IV Cannula-Catheter	65.2	15
		IV Fluid	65.2	15
	Treat (Durables)	IV Pole	65.2	15
		Refrigeration [8]	65.2	15
	Treat (Treatments)	Parenteral Uterotonic [9]	60.9	14
	Monitor-Modify	Sphygmomanometer	52.2	12
		Stethoscope	52.2	12
		Uterotonic, Non-oxytocin [10]	21.7	5
		Urinary catheter	21.7	5
		Oxygen source [11]	21.7	5
		Oxygen tubing	17.4	4
		Oxygen delivery [12]	17.4	4
		Protocol: Hemorrhage	13.0	3
Manage Hypertensive Emergencies (*Anticonvulsant*)	Identify	Sphygmomanometer	91.3	21
		Stethoscope	78.3	18
		Urine Cup	43.5	10
		Urine Dipstick	30.4	7
	Treat (Consumables)	IV Tubing	30.4	7

		IV Cannula-Catheter	30.4	6
		IV Fluid	26.1	6
	Treat (Durables)	IV Pole	26.1	6
	Treat (Treatments)	Parenteral Anticonvulsant [13]	26.1	6
		Parenteral Antihypertensive [14]	26.1	6
	Monitor-Modify	Urinary catheter	26.1	6
		Calcium Gluconate	17.4	4
		Oxygen source [11]	17.4	4
		Oxygen tubing	13.0	3
		Oxygen delivery [12]	13.0	3
		Protocol: Eclampsia	13.0	3

Manage Retained Placenta *(Manual removal of retained placenta)*	Identify	Staff Skill [6]	100.0	23
		Light Source [15]	100.0	23
	Treat (Consumables)	Gloves, Aseptic [7]	78.3	18
		IV Tubing	69.6	16
		IV Cannula-Catheter	65.2	15
		IV Fluid	65.2	15
	Treat (Durables)	IV Pole	65.2	15
		Refrigeration [8]	65.2	15
	Treat (Treatments)	Parenteral Uterotonic (Oxytocin)	60.9	14
		Parenteral Sedative (Diazepam)	43.5	10
		Parenteral Antibiotic-Step 1 [3]	43.5	10
	Monitor-Modify	Sphygmomanometer	34.8	8
		Stethoscope	34.8	8
		Uterotonic, non-oxytocin [10]	17.4	4
		Parenteral Antibiotic-Step 2 [4]	13.0	3
		Urinary catheter	13.0	3
		Protocols [16]	8.7	2
Manage Incomplete Abortion *(Manual removal of retained products of conception)*	Identify	Speculum	100.0	23
		Light Source [15]	100.0	23
	Treat (Consumables)	Gloves, Sterile [7]	78.3	18
		IV Tubing	69.6	16
		IV Cannula-Catheter	65.2	15
		IV Fluid	65.2	15

	Treat (Durables)	Manual Vacuum Aspirator Kit (17)	56.5	13
		IV Pole	56.5	13
	Treat (Treatments)	Local Anesthetic (Lidocaine)	56.5	13
		Parenteral Antibiotic-Step 1 (3)	56.5	13
	Monitor-Modify	Sphygmomanometer	52.2	12
		Stethoscope	47.8	11
		Refrigeration (8)	47.8	11
		Uterotonic, non-oxytocin (10)	17.4	4
		Parenteral Antibiotic-Step 2 (4)	13.0	3
		Catheter, Urinary	13.0	3
		Protocols (18)	8.7	2

(1) Total sample n=23 facilities
(2) Normal saline (NS) or Lactated Ringer's (LR)
(3) Parenteral ampicillin or parenteral penicillin
(4) Parenteral gentamycin or cefotaxime/ceftriaxone
(5) Puerperal sepsis, infection or antibiotic administration protocol
(6) 100% staff skill for identifying the emergency was assumed
(7) Sterile gloves or aseptic disposable gloves were reported as a single item in the dataset
(8) Power used as a proxy for refrigeration
(9) Oxytocin (as tranexamic acid and carbetocene were not available in the dataset
(10) Misoprostol tablets or ergometrine (IM)
(11) Oxygen concentrator plus power or oxygen cylinder and oxygen head
(12) Oxygen mask or nasal cannula
(13) Magnesium sulfate IV
(14) Hydralazine or Labetalol or Nifedipine or Methyldopa
(15) Flashlight or power (as proxy for electric lights
(16) Protocol for retained placenta or hemorrhage or infection
(17) Manual vacuum aspirator device and MVA cannula
(18) Protocol for incomplete abortion, hemorrhage or infection

Table S8. Mean Drop-Off by Cascade and Stage of Care by Reported C-Section Capability

**Readiness Drop-Off by Stage of Care**							**Readiness Drop-Off by Emergency**			
**Clinical Cascade**	**1**		**2**		**3**		***Mean Drop-Off Across 3 Cascade Stages of Care***		***SD***	
	**Identify**		**Treat**		**Monitor-Modify**					
**Reported C-Section Capability**	**C-section** [1]	**No C-section** [2]	**C-section**	**No C-section**	**C-section**	**No C-section**	**C-section**	**No C-section**	**C-section**	**No C-section**
	--		--		--		**26.7%**[3]	**29.7%**	24.8[4]	29.5
**Sepsis-Infection**	40.0%	15.4%	10.0%	38.5%	10.0%	23.1%	20.0%	25.6%	17.3	11.8
**Hemorrhage**	0.0%	0.0%	30.0%	46.2%	60.0%	38.5%	30.0%	28.2%	30.0	24.7
**Hypertensive Emergency**	60.0%	76.9%	10.0%	0.0%	20.0%	7.7%	30.0%	28.2%	26.5	42.4
**Retained Placenta**	0.0%	0.0%	30.0%	76.9%	50.0%	23.1%	26.7%	33.3%	25.2	39.5
**Incomplete Abortion**	0.0%	0.0%	30.0%	53.9%	50.0%	46.2%	26.7%	33.3%	25.2	29.1
**Overall Drop-Off by Stage of Care**										
Drop-Off by Stage of Care (Across All Emergencies), Pooled Mean	**20.0%**	**18.5%**	**22.0%**	**43.1%**	**38.0%**	**27.7%**				
SD	28.3	33.4	11.0	28.1	21.7	15.0	4.1[5]	3.4		

(1) n = 10 facilities
(2) n = 13 facilities
(3) Pooled mean readiness drop-off across 3 clinical cascade stages of care and 5 emergencies
(4) Mean of the standard deviations
(5) Standard deviation across 3 stages of care and 5 emergency cascades

Table S9. Mean Drop-Off in Readiness by Cascade and Stage of Care by Ownership

Readiness Drop-Off by Stage of Care							Readiness Drop-Off by Emergency			
**Clinical Cascade**	**1**		**2**		**3**		***Mean Drop-Off Across 3 Cascade Stages of Care***		***SD***	
	**Identify**		**Treat**		**Monitor-Modify**					
**Ownership**	**Gov.** [1]	**Private** [2]	**Gov.**	**Private**	**Gov.**	**Private**	**Gov.**	**Private**	**Gov.**	**Private**
	--		--		--		**29.1%** [3]	**25.0%**	**24.7** [4]	**35.7**
**Sepsis-Infection**	26.3%	25.0%	31.6%	0.0%	21.1%	0.0%	26.3%	8.3%	5.3	14.4
**Hemorrhage**	0.0%	0.0%	42.1%	0.0%	42.1%	100.0%	28.1%	33.3%	24.3	57.7
**Hypertensive Emergency**	68.4%	75.0%	5.3%	0.0%	10.5%	25.0%	28.1%	33.3%	35.0	38.2
**Retained Placenta**	0.0%	0.0%	63.2%	0.0%	31.6%	75.0%	31.6%	25.0%	31.6	43.3
**Incomplete Abortion**	0.0%	0.0%	47.4%	25.0%	47.4%	50.0%	31.6%	25.0%	27.4	25.0
**Overall Drop-Off by Stage of Care**										
Drop-Off by Stage of Care (Across All Emergencies), Pooled Mean	**19.1%**	**20.0%**	**37.9%**	**5.0%**	**30.5%**	**50.0%**				
SD	**29.9**	**32.6**	**21.5**	**11.2**	**15.1**	**39.5**	**2.4** [5]	**10.2**		

[1] n = 19 facilities
[2] n = 4 facilities
[3] Pooled mean readiness drop-off across 3 clinical cascade stages of care and 5 emergencies
[4] Mean of the standard deviations
[5] Standard deviation across 3 stages of care and 5 emergencies

Table S10. Mean Drop-Off in Readiness by Cascade and Stage of Care by Country

**Readiness Drop-Off by Stage of Care**							**Readiness Drop-Off by Emergency**			
**Clinical Cascade**	**1**		**2**		**3**		***Mean Drop-Off Across 3 Cascade Stages of Care***		***SD***	
	**Identify**		**Treat**		**Monitor-Modify**					
**Country**	**Kenya**[1]	**Uganda**[2]	**Kenya**	**Uganda**	**Kenya**	**Uganda**	**Kenya**	**Uganda**	**Kenya**	**Uganda**
	--		--		--		**27.8%**[3]	**30.0%**	26.7[4]	24.0
**Sepsis-Infection**	17.7%	50.0%	29.4%	16.7%	17.7%	16.7%	21.6%	27.8%	6.8	19.3
**Hemorrhage**	0.0%	0.0%	41.2%	33.3%	41.2%	66.7%	27.5%	33.3%	23.8	33.3
**Hypertensive Emergency**	76.5%	50.0%	0.0%	16.7%	5.9%	33.3%	27.5%	33.3%	42.6	16.7
**Retained Placenta**	0.0%	0.0%	64.7%	33.3%	29.4%	50.0%	31.4%	27.8%	32.4	25.5
**Incomplete Abortion**	0.0%	0.0%	41.2%	50.0%	52.9%	33.3%	31.4%	27.8%	27.8	25.5
**Overall Drop-Off by Stage of Care**										
Drop-Off by Stage of Care (Across All Emergencies), Pooled Mean	**18.8%**	**20.0%**	**35.3%**	**30.0%**	**29.4%**	**40.0%**				
SD	**33.1**	**27.4**	**23.5**	**13.9**	**18.6**	**19.0**	**4.0**[5]	**3.0**		

(1) n = 17 facilities
(2) n = 6 facilities
(3) Pooled mean readiness drop-off across 3 clinical cascade stages of care and 5 emergencies
(4) Mean of the standard deviations
(5) Standard deviation across 3 stages of care and 5 emergency cascades

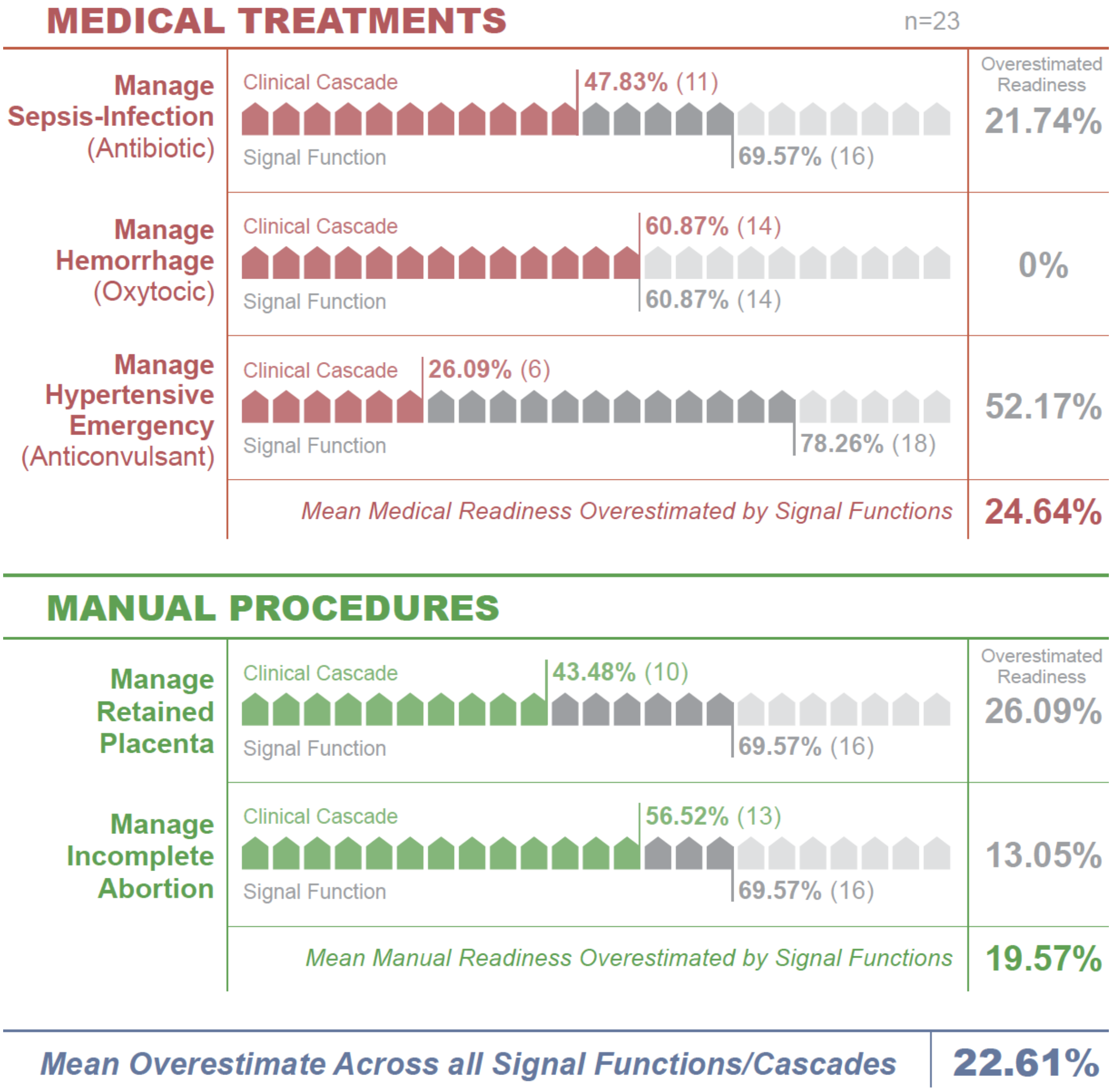

Figure S1. Mean Overestimate Across All Signal Functions and Cascades

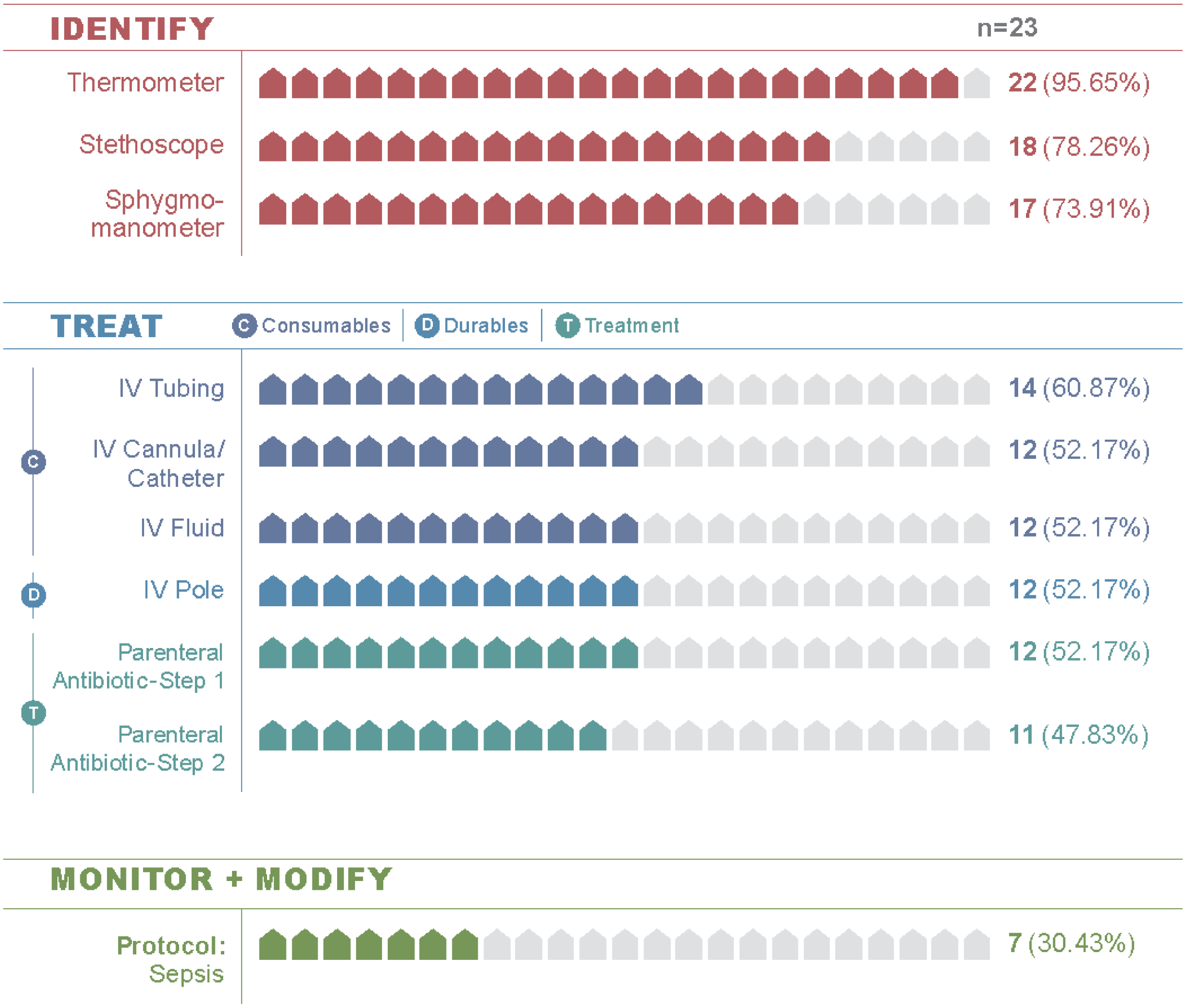

Figure S2. Sepsis Clinical Cascade

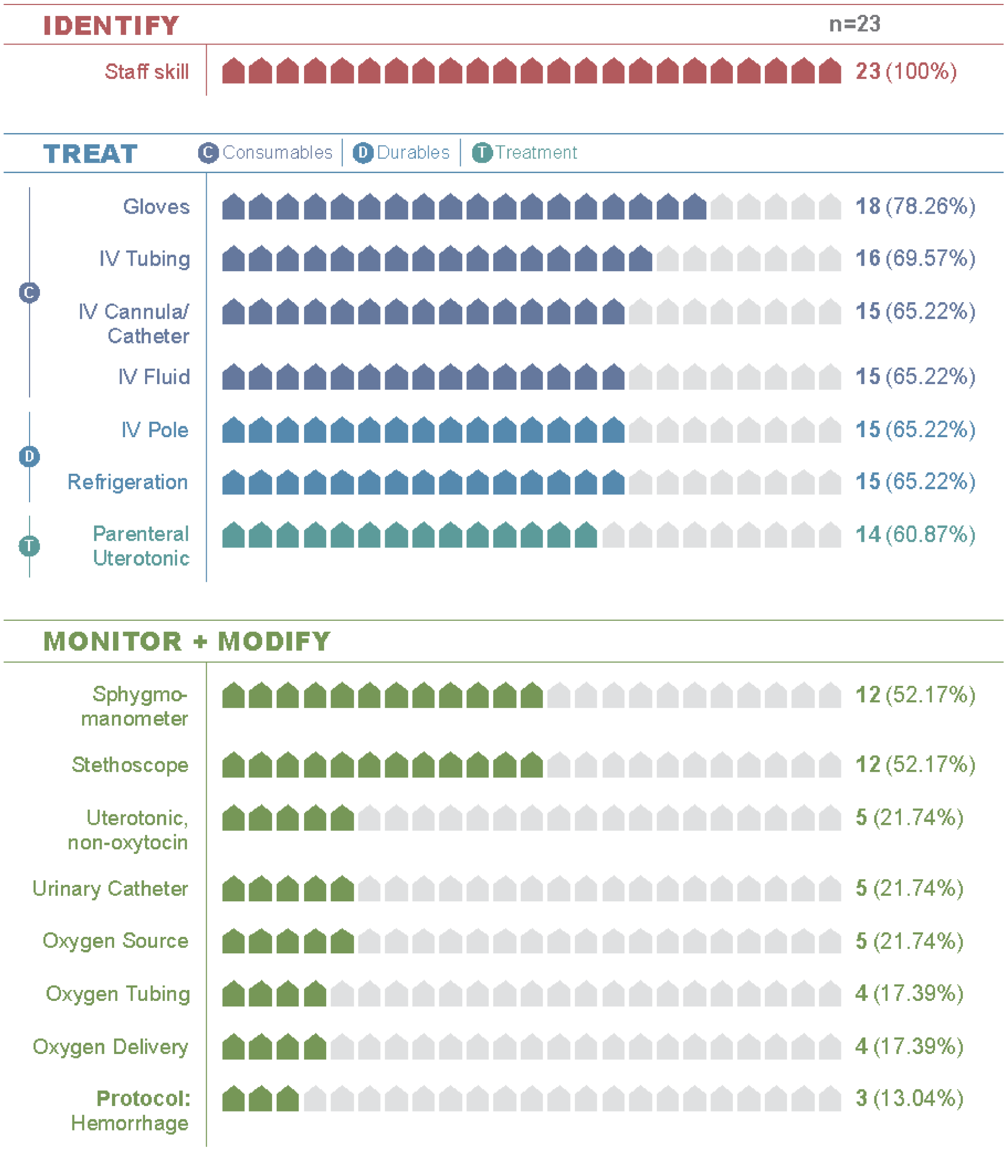

Figure S3. Hemorrhage Clinical Cascade

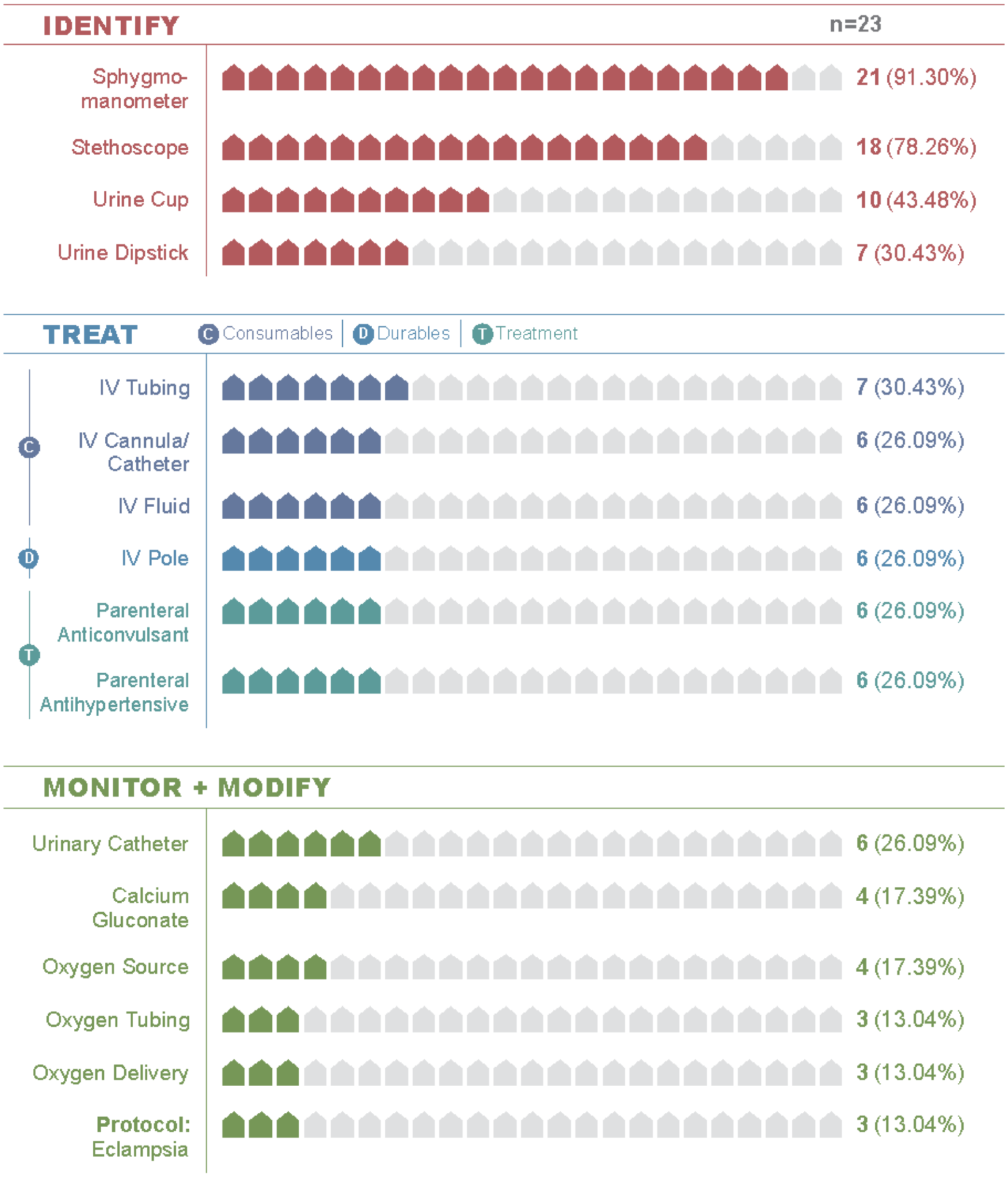

Figure S4. Hypertensive Emergency Clinical Cascade

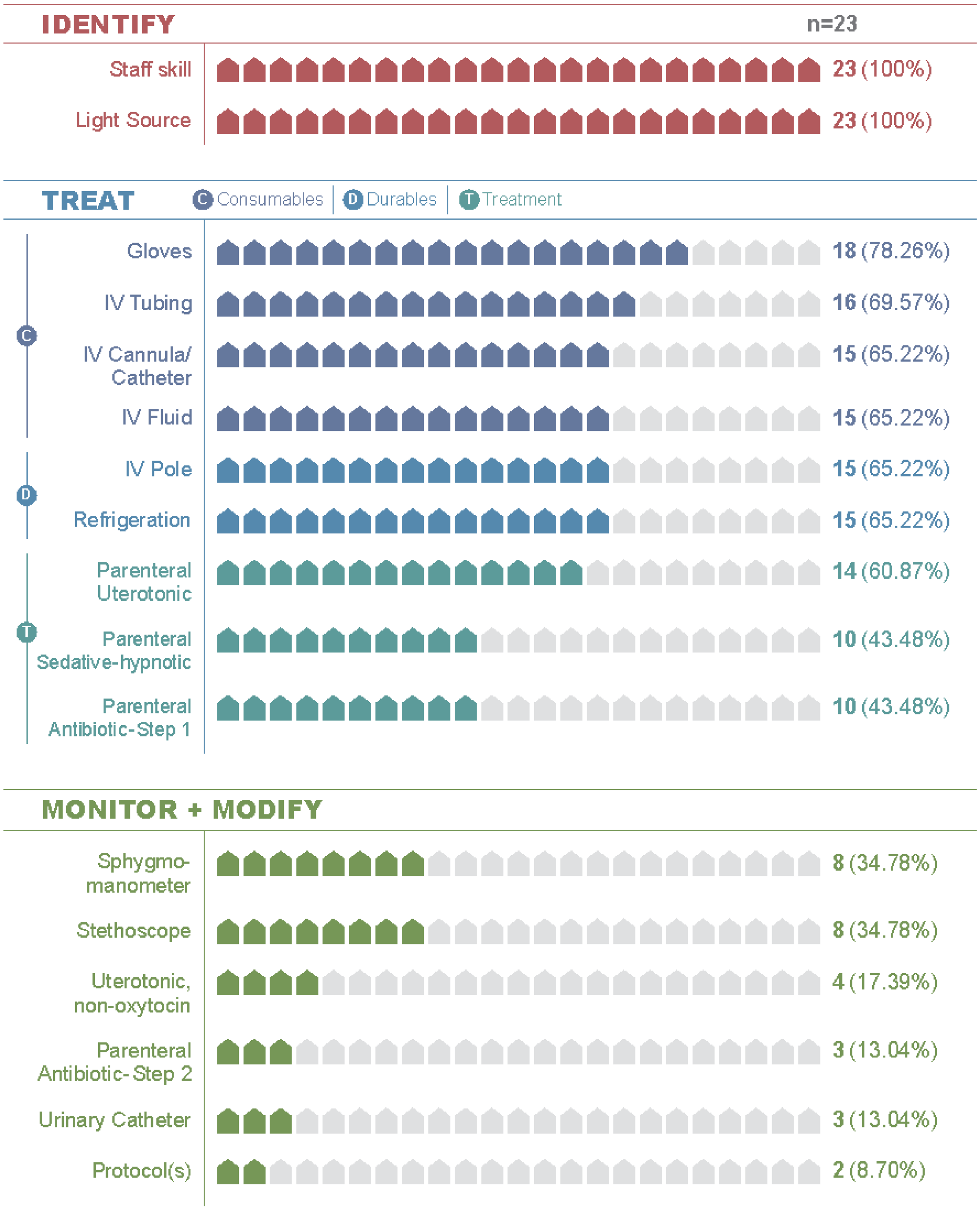

Figure S5. Retained Placenta Clinical Cascade

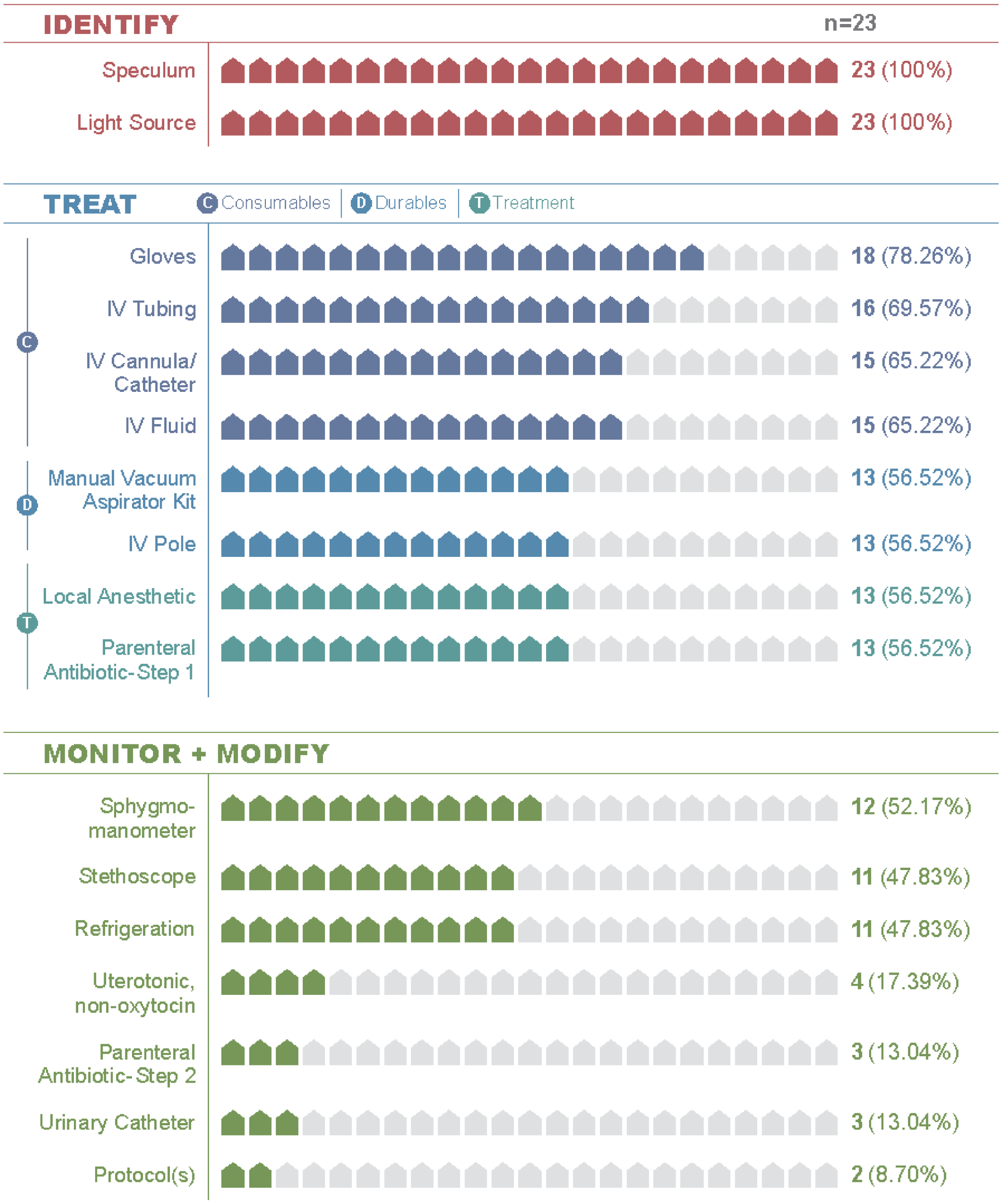

Figure S6. Incomplete Abortion Clinical Cascade

REFERENCES

[1] World Health Organization. Health statistics and information systems: maternal mortality ratio (per 100,000 live births), 2020. Available: https://www.who.int/healthinfo/statistics/indmaternalmortality/en/.

[2] World Health Organization. Monitoring emergency obstetric care: a handbook. Geneva, Switzerland: Department of Reproductive Health and Research, 2009.

[3] Harvard School of Medicine. Abortion (Termination of Pregnancy), 2019. Available: https://www.health.harvard.edu/medical-tests-and-procedures/abortion-termination-of-pregnancy-a-to-z.

[4] Cranmer JN, Dettinger J, Calkins K, et al. Beyond signal functions in global obstetric care: Using a clinical cascade to measure emergency obstetric readiness. PLoS One. 2018;13:e0184252. doi:10.1371/journal.pone.0184252

[5] Morgan MC, Spindler H, Nambuya H, et al. Clinical cascades as a novel way to assess physical readiness of facilities for the care of small and sick neonates in Kenya and Uganda. *PLoS One*. 2018;13:e0207156. doi:10.1371/journal.pone.0207156

[6] Brenner S, De Allegri M, Gabrysch S, et al. The quality of clinical maternal and neonatal healthcare – A strategy for identifying 'routine care signal functions'. *PLoS One.* 2015;10:e0123968. doi:10.1371/journal.pone.0123968
