# Supplementary material for: Using clinical cascades to measure health facilities’ obstetric emergency readiness: testing the cascade model using cross-sectional facility data in East Africa
Source: BMJ Open. 2022 Apr 3;12(4):e057954. doi: 10.1136/bmjopen-2021-057954 (PMC8981352; doi:10.1136/bmjopen-2021-057954)
Supplement: Author's manuscript [file bmjopen-2021-057954.draft_revisions.pdf]

BMJ Open is committed to open peer review. As part of this commitment we make the peer review history of every article we publish publicly available.

When an article is published we post the peer reviewers' comments and the authors' responses online. We also post the versions of the paper that were used during peer review. These are the versions that the peer review comments apply to.

The versions of the paper that follow are the versions that were submitted during the peer review process. They are not the versions of record or the final published versions. They should not be cited or distributed as the published version of this manuscript.

BMJ Open is an open access journal and the full, final, typeset and author-corrected version of record of the manuscript is available on our site with no access controls, subscription charges or pay-per-view fees (http://bmjopen.bmj.com).

If you have any questions on BMJ Open's open peer review process please email info.bmjopen@bmj.com

BMJ Open

Using Clinical Cascades to Measure Health Facilities' Obstetric Emergency Readiness: An Analysis of Cross-Sectional Facility Assessments in East Africa

Journal:	*BMJ Open*
Manuscript ID	bmjopen-2021-057954
Article Type:	Original research
Date Submitted by the Author:	02-Oct-2021
Complete List of Authors:	Whaley, Bridget; Emory University, Behavioral, Social, and Health Education Sciences<br>Butrick, Elizabeth ; University of California San Francisco<br>Sales, Jessica; Emory University, Behavioral, Social, and Health Education Sciences<br>Wanyoro, Anthony; Kenyatta University, Department of OB/GYN<br>Waiswa, Peter; Makerere University, School of Public Health<br>Walker, Dilys; University of California San Francisco Department of Obstetrics Gynecology and Reproductive Sciences, ; University of California San Francisco Global Health Sciences,<br>Cranmer, John; Emory University Woodruff Health Sciences Center,
Keywords:	OBSTETRICS, Maternal medicine < OBSTETRICS, International health services < HEALTH SERVICES ADMINISTRATION & MANAGEMENT, Quality in health care < HEALTH SERVICES ADMINISTRATION & MANAGEMENT

SCHOLARONE™
Manuscripts

I, the Submitting Author has the right to grant and does grant on behalf of all authors of the Work (as defined in the below author licence), an exclusive licence and/or a non-exclusive licence for contributions from authors who are: i) UK Crown employees; ii) where BMJ has agreed a CC-BY licence shall apply, and/or iii) in accordance with the terms applicable for US Federal Government officers or employees acting as part of their official duties; on a worldwide, perpetual, irrevocable, royalty-free basis to BMJ Publishing Group Ltd ("BMJ") its licensees and where the relevant Journal is co-owned by BMJ to the co-owners of the Journal, to publish the Work in this journal and any other BMJ products and to exploit all rights, as set out in our licence.

The Submitting Author accepts and understands that any supply made under these terms is made by BMJ to the Submitting Author unless you are acting as an employee on behalf of your employer or a postgraduate student of an affiliated institution which is paying any applicable article publishing charge ("APC") for Open Access articles. Where the Submitting Author wishes to make the Work available on an Open Access basis (and intends to pay the relevant APC), the terms of reuse of such Open Access shall be governed by a Creative Commons licence – details of these licences and which Creative Commons licence will apply to this Work are set out in our licence referred to above.

Other than as permitted in any relevant BMJ Author's Self Archiving Policies, I confirm this Work has not been accepted for publication elsewhere, is not being considered for publication elsewhere and does not duplicate material already published. I confirm all authors consent to publication of this Work and authorise the granting of this licence.

For peer review only

TITLE PAGE

Article Title:
Using Clinical Cascades to Measure Health Facilities' Obstetric Emergency Readiness: An Analysis of Cross-Sectional Facility Assessments in East Africa

Corresponding Author:
Bridget Whaley
1520 Clifton Road NE
Atlanta, GA, 30322
whaley.bridget@gmail.com

Author Information:
Bridget Whaley[1], Elizabeth Butrick[2], Jessica M. Sales[1], Anthony Wanyoro[3], Peter Waiswa[4], Dilys Walker[2], John N. Cranmer[5]

1 Rollins School of Public Health, Emory University, Atlanta, Georgia, United States of America
2 Department of Obstetrics, Gynecology, and Reproductive Sciences, University of California San Francisco, San Francisco, California, United States of America
3 Department of Obstetrics and Gynecology, Kenyatta National Hospital, University of Nairobi, Nairobi, Kenya
4 School of Public Health, Makerere University, Kampala, Uganda
5 Emory University, Atlanta, Georgia, United States of America

Funding: Bill & Melinda Gates Foundation (Grant # OPP1107312) funded primary data collection and article processing charges. Emory University Rollins School of Public Health's Global Field Experience Financial Award funded effort for the literature review.

Competing Interests: AW represents his university on the Sexual, Reproductive, and Child Health Committee for the Bill & Melinda Gates Foundation. JNC has grants from UNICEF and is an Advisory Board Member for Towards Unity in Health and Chair for the Community-Based Primary Health Care working group with the American Public Health Association, International Health Section. DW is on the Board of Director of PRONTO International, a not-for-profit agency. The other authors declare no competing interests.

Data availability statement: Data are available upon reasonable request. The deidentified facility-level data underlying this nested analysis and the results presented in this Article are available to researchers who present ethically sound proposals to DW, the principal investigator of the parent study. Requestors will need to sign a data access agreement that is subject to approval by the parent study principal investigators.

Abstract Word Count: 300

Manuscript Word Count: 4,826

Number of Tables and Figures (manuscript): 5

Number of Tables and Figures (Online Supplementary Document): 15

For peer review only

ABSTRACT

Introduction: Globally, hundreds of women die each day from preventable pregnancy- and childbirth-related causes, with the greatest burden in Sub-Saharan Africa. Five key conditions - severe bleeding, infections, high blood pressure, delivery complications, and unsafe abortions - account for nearly 75% of these deaths. Skilled health providers with strategic emergency supplies could prevent most of these deaths. This study: (1) measured facility readiness to manage common obstetric emergencies using the clinical cascades and signal functions; (2) compared these estimates by level of care, facility ownership, and country; and (3) measured the cascading drop-off in emergency resources.

Methods: Data were collected in 2016 from 23 facilities in the Preterm Birth Initiative (PTBi) study in East Africa (Migori County, Kenya and Busoga region, Uganda). Baseline data from these facilities were used to estimate a facility's readiness to manage common obstetric emergencies using the signal functions and clinical cascades. We compared emergency readiness estimates using the proportion of tracer items (signal functions) and the proportion of items needed to identify and treat the emergency (cascades).

Results: The signal functions overestimated practical obstetric emergency readiness by 23% across five emergencies. Not all comprehensive emergency obstetric care (CEmOC) facilities could perform basic emergency obstetric care (BEmOC), with 42% of CEmOC facilities unable to perform BEmOC based on cascade estimates (20% according to signal function estimates). Across the three stages of care and five emergencies, there was a consistent 28% drop-off in emergency readiness, with the largest drop off occurring in the treatment stage. Patterns in drop off remained largely consistent across level of care, facility ownership, and country.

Conclusions: Accurate measurement of obstetric emergency readiness is a prerequisite for strengthening facilities' capacity to manage common emergencies. The cascades present stepwise, emergency-specific readiness estimates that can improve policies and programs to decrease global maternal death rates.

STRENGHTS & LIMITATIONS

- Measuring emergency obstetric readiness according to the clinical cascades provides a more nuanced picture of where key resources are lost for emergency care and can support targeted strategies to optimize critical emergency obstetric supplies for maternal survival.
- To the authors' knowledge, this is the first study to compare signal functions and clinical cascade estimates of obstetric emergency readiness across multiple countries.
- This study expands upon previous scholarship by highlighting emergency readiness estimates by level of care (C-section capability), facility ownership (government vs. private mission hospitals), and country (Kenya and Uganda).
- We did not have data to estimate cascading resource availability losses for assisted vaginal deliveries—one of six signal functions.
- Only measuring the resource availability does not provide a complete picture of emergency readiness as the clinicians' ability to use the resources effectively is critical but difficult to measure.

For peer review only

INTRODUCTION

In 2017, more than 800 women died each day from preventable pregnancy- and childbirth-related causes, which contributed to a global rate of nearly 300,000 deaths annually[1,2]. A few major complications account for nearly 75% of all maternal deaths: severe bleeding (hemorrhage), infections (sepsis), high blood pressure (pre-eclampsia and eclampsia), delivery complications (prolonged or obstructed labor), and unsafe abortions[3]. These deaths can be prevented when skilled health care providers, supported with the supplies necessary to identify and manage emergencies, attend births and can transfer patients when an emergency exceeds the facility's scope of management[3]. Labor complications require immediate and easy access to high-quality emergency obstetric care (EmOC). Basic Emergency Obstetric Care (BEmOC), which efficiently treats the majority of causes of maternal death, requires essential supplies, durable equipment, and targeted drugs. When emergencies are beyond the capacity of BEmOC facilities, patients are referred to Comprehensive Emergency Obstetric Care (CEmOC) facilities. CEmOC builds on BEmOC and also includes resources for blood transfusion and surgery[3]. Mobilizing and dispensing the clinical resources required to manage basic obstetric emergencies may be a critical step for reducing persistently elevated Maternal Mortality Ratios (MMRs) in Sub-Saharan African contexts such as Kenya and Uganda. Therefore, accurate measurement of a facility's readiness to manage the common emergencies that drive maternal deaths is urgently needed.

The World Health Organization (WHO) first identified the resources necessary to manage common obstetric emergencies in the 1990s[4]. This recommended approach evolved into the "signal functions," consisting of six clinical actions (three medical treatments and three manual procedures) used during obstetric emergencies. For BEmOC, the medical treatments are administering parenteral (1) antibiotics, (2) uterotonic drugs, and (3) anticonvulsants/antihypertensives and the manual procedures include (4) manually removing retained placentas, (5) removing retained products of conception, and (6) performing assisted vaginal deliveries[5]. CEmOC adds two additional actions: perform (7) surgeries such as Caesarean section (C-section) and (8) blood transfusion[5]. Thus, all CEmOC facilities should be prepared to perform all BEmOC signal functions, in addition to performing surgery and blood transfusion. Specific items (i.e., "tracers") are used as proxies to measure a facility's capacity to handle obstetric emergencies in the signal function model[5-11].

Signal functions are the dominant method for assessing obstetric emergency readiness at facilities worldwide[12-15]. They provide one aggregate estimate of overall facility readiness—the percent of tracer items present at the facility[15]. However, in recent years, scientists and practitioners have increasingly called for revised approaches[7,9,16-21]. One particular weakness is the signal function's inability to predict readiness for *specific* emergencies[16,18]. Further, this approach does not identify strategic resources for managing multiple emergencies nor suggest indicators for system-wide emergency readiness[21]. Also, the signal functions do not distinguish between the resources required to first *identify* the emergency/disorder and the consumable supplies and durable goods required to deliver the treatment or perform the procedure[21]. Thus, to further reduce delivery-related mortality, a more robust set of measurable indicators that are emergency/outcome-specific and relevant for multiple levels of the health system is needed[21]. The clinical cascade model aims to achieve this.

The cascade model builds off of both the signal functions and two underlying frameworks - the Systemic Capacity Hierarchy of Needs and Spectrum of Engagement[22,23]. The cascades emerged as a clinically-oriented approach to measuring facility readiness by reporting the step-wise relationship among emergency resources[21-23]. The cascade model defines emergency readiness as the proportion of facilities that can *identify* an emergency and have available the resources (including drugs, supplies, and equipment) to *treat* the identified emergency. Facilities' ability to *monitor and modify* the initial treatment as clinically indicated has been proposed as a care quality metric[21].

For peer review only - http://bmjopen.bmj.com/site/about/guidelines.xhtml

Unlike the wealth of research on signal functions, the clinical cascades have been tested in fewer geographic locations. There are limited studies comparing this emerging metric with the signal functions as methods to assess facilities' readiness to handle basic obstetric emergencies. To date, one study compared estimates of readiness based on signal functions and clinical cascades. Conducted among a sample of BEmOC facilities in Kakamega County, Kenya, the study found that, across the emergencies driving maternal mortality, signal functions overestimated practical emergency management readiness by 55%[21]. Moreover, a consistent 33% drop off in readiness emerged across all cascades (emergencies) and stages of care (identification, treatment, monitor/modify)[21]. Similarly, a study examining the clinical cascades as applied to neonatal care in Kenya and Uganda confirmed a similar 30% mean drop off across all emergencies and stages[24]. This study, however, did not compare clinical cascade-based estimates of readiness against signal function estimates because no standard signal function indicators for newborn emergencies exist[9]. The findings from these two studies warrant additional investigation into the relevance and transferability of cascade analysis to additional global contexts, health systems, and levels of facility care. A more comprehensive analysis of a facility's readiness to handle obstetric emergencies, such as the cascade analysis, may be critical to guide emergency-specific or supply-specific interventions to close gaps in the availability of emergency supplies.

Aims

Building off the wealth of research on the signal functions and formative research on clinical cascades, this cross-sectional facility-based study was designed to assess readiness for pregnancy- related emergencies at BEmOC and CEmOC facilities in Migori County, Kenya and the Busoga region in Uganda. The facility data used in the analysis of emergency obstetric readiness was originally collected as part of a pair-matched, cluster randomized controlled trial evaluating a package of facility-based interventions to improve care for preterm infants[25]. This nested analysis was designed to: (1) estimate facility readiness to manage common obstetric emergencies using clinical cascades and signal functions; (2) compare these estimates between levels of care, facility ownership, and country; and (3) test if the cascading loss of emergency obstetric resources first identified in Kenya held true in other global settings. The limited number of published studies suggested that using signal functions to assess readiness would convey a false sense of security by indicating a higher level of emergency obstetric preparedness than actually exists in facilities (based on clinical cascade estimates)[21,24].

METHODS

Study Design and Data Collection

We conducted a cross-sectional analysis of facility data collected in 2016-2019 by the Preterm Birth Initiative Kenya and Uganda Implementation Research Collaborative (PTBi)[25]. Data from pre-intervention baseline facility assessments at 23 facilities in the PTBi study were used to create the signal function and clinical cascade estimates of emergency obstetric readiness[25]. In all, data from 17 PTBi facilities in Migori County (western Kenya) and 6 in Busoga Region (eastern Uganda) were used. For this study, we used the baseline data (2016) to accurately capture standard facility readiness prior to PTBi intervention.

During data collection, research assistants used standardized forms to visually identify emergency resources in each facility. During the physical inventory, the team captured information about facility demographics, obstetric drugs, consumable supplies, durable goods, and existing guidelines and protocols. Researchers recorded both the presence/absence of the item and its location (i.e., Unit). In this analysis, we used a resource's presence or absence to estimate facility-level readiness regardless of the unit in which the items were located.

For peer review only

Emergency Readiness

Signal Functions

WHO's Service Readiness Index (SRI) defines a facility's obstetric emergency readiness using the six clinical actions that define BEmOC[5]. In the SRI methodology, the average number of tracer items present on the day of the facility assessment determines a facility's overall obstetric emergency readiness[14,15]. This method utilizes the signal functions framework. Traditionally, signal function readiness estimates are reported as a single indicator, the proportion of facilities with the tracer items for all medical treatments and manual procedures[21]. However, for the purposes of our analysis, we reported additional indicators: (1) total readiness estimates across all signal functions; (2) readiness estimates by type of signal function (medical treatment vs. manual procedure); and (3) individual readiness estimates for each signal function. This allowed for a direct comparison to clinical cascade estimates.

Clinical Cascades

Given that the signal functions do not have quality indicators for monitoring and modifying the primary emergency treatment based on a patient's clinical response, we defined mean clinical cascade readiness using stages 1 and 2 of the clinical cascades (identify and treat). We measured clinical cascade estimates of readiness across facilities at the individual resource level. This approach allowed us to demonstrate precisely where in the clinical cascade of emergency care readiness drops off. We estimated overall emergency readiness across sites as the percentage of facilities with all resources necessary to complete the *identify* and *treat* stages of care.

Operational definitions

In the absence of certain variables, we used proxies for the missing tracer items to not unduly penalize a facility's readiness estimate using the signal functions. This was especially true for consumable supplies and durable goods, such as refrigeration (electrical power as proxy) and light source (electrical power or flashlight as proxy). Moreover, some tracer items, namely drugs, are not concretely defined in the signal functions model. When drugs were not explicitly defined in the signal functions, we referred to WHO's first-line recommendations for obstetric care[5,26,27]. For instance, in the signal functions, antibiotics are broadly defined as "parenteral antibiotics." To transform this reference into a specific, measurable variable, we deferred to WHO's 3-step sequence of obstetric antibiotic therapy escalation to define readiness (Ampicillin, Gentamicin, and Metronidazole)[5,26,27]. In other instances, data on both tracers and potential proxies were missing. This was true for most items from the assisted vaginal delivery cascade. Consequently, we were unable to measure assisted vaginal delivery readiness using the signal functions or clinical cascades. See **Table S1** in the **Online Supplementary Document** for a list of all items used to define the signal functions and clinical cascades.

Analysis

In describing the availability of key items, we reported percentages and frequencies for categorical variables and medians with interquartile ranges (IQR) for continuous variables because variables were not normally distributed (e.g., delivery volume). Aggregate readiness across multiple emergencies or multiple stages was reported as the overall mean. Drop-offs in readiness (i.e., the percentage of facilities prepared to handle the five emergencies) between each stage and across each cascade were reported as percentages, with accompanying measures of spread (i.e., standard deviation). We reported estimates of overall emergency readiness as means for several reasons: 1) the SRI methodology, upon which this study is based, reports means; (2) reporting means is in line with the previously published literature; and (3) with so few observations, the median may not effectively capture central tendency.

For the purposes of comparing the clinical cascades and signal functions, we reported: (1) mean overall readiness across all signal functions/clinical cascades; (2) mean readiness by type of signal function (medical treatment and manual procedure); (3) mean readiness for each signal function/clinical cascade; and (4) overestimated readiness (signal function mean readiness minus clinical cascade mean readiness).

For peer review only

Mean overall readiness was calculated by averaging the individual readiness estimates for each signal function/clinical cascade. All emergency readiness estimates were reported for all facilities. We stratified the findings by reported C-section capability, ownership (public/government- versus private/mission owned), and country (Kenya vs. Uganda). Reported C-section service delivery at a facility was used as a proxy for identifying CEmOC facilities as BEmOC facilities do not have this capability.

For the purpose of calculating the drop off in readiness according to the clinical cascade model, we reported the percentage decrease in facilities ready to handle the five emergencies by stage of the clinical cascade, by emergency, and overall across the three stages and five emergencies. To determine readiness drop off by stage, we subtracted readiness (i.e., percentage of facilities ready to handle the five emergencies) at the end of a stage from readiness at the end of the preceding stage. For example, to calculate readiness drop off during stage 1 (identify), we subtracted readiness at the end of the stage from 100% and then calculated the mean and SD across the five clinical cascades for each stage to generate the mean readiness drop off from stage-to-stage (identify, treat, monitor-modify). From these estimates, we calculated drop off by clinical cascade by averaging across the three stages for each clinical cascade (i.e., emergency) and producing five estimates of mean drop off in readiness. The mean across the five cascades determined the overall drop off in readiness across emergencies and stages. Estimates were stratified by (1) reported C-section capability; (2) facility ownership; and (3) country. For these sub-analyses, we used Fisher's Exact Test to compare readiness estimates for significance. We compared readiness estimates across these three strata (C-section capacity, public/private ownership, and Kenya vs. Uganda) at both stage 2 and stage 3 of the clinical cascade of care for each emergency.

Patient and Public Involvement

As this was a nested analysis of previously collected facility-level data, patients and the public were not involved in the design, conduct, reporting, or dissemination plans of this research. In the parent study, patients were not involved in the design or the conduct of the study. The research question and outcome measures were shaped by global priorities and the experiences of women. The parent study results were shared with health providers at the study facilities, but not with individual patients. As the parent trial was cluster-randomized at the facility level, and all interventions were directed at health workers, women did not directly assess intervention burden.

Ethics

The Kenya Medical Research Institute (KEMRI/SERU/CCR/0034/3251), Makerere University School of Public Health (MUSPH HDREC 395), and the UCSF (16-19162) Institutional Review Boards (IRBs) approved the trial prior to primary data collection. Emory University's IRB determined the nested analysis of these de-identified data was not human subjects research (STUDY 00001202).

RESULTS

Facility Characteristics

In **Tables S2-S3,** we present demographic results for the study sample. Of the 23 facilities, 82.61% were government-owned and 17.39% were mission (privately-owned) hospitals. All Ugandan facilities reported C-section capability and are, therefore, designed to offer CEmOC. By contrast, only 23.53% of the Kenyan facilities were CEmOC facilities.

Signal Function Estimates of Emergency Readiness

Across the three medical treatments and two manual procedures, the signal functions estimated a mean overall emergency readiness of 69.57% (**Table 1**). Readiness estimates were fairly consistent across the five maternal signal functions, ranging from 60.87% (oxytocic) to 78.26% (anticonvulsant), and were nearly identical for both medical and manual signal functions (69.57%).

For peer review only

As expected, signal function estimates differed by the facility's level of care (i.e., reported C-section capability versus no C-section capability), facility ownership, and country. Overall, BEmOC readiness estimates were higher among facilities with C-section capability (CEmOC facilities), those that were privately-owned (mission facilities), and those in Uganda. Specifically, mean overall readiness across the five signal functions was 80.00% among facilities with C-section capability, compared to 61.54% among facilities without C-section capability (**Table S4**). Mean overall readiness across the five signal functions was 65.26% among government facilities, compared to 95.00% among mission facilities (**Table S5**). When stratified by country, signal function estimates of readiness were slightly higher among Ugandan compared to Kenyan facilities (mean readiness 73.33% versus 68.24%, **Table S6**).

For peer review only

Table 1. Comparison of Emergency Readiness Using Clinical Cascades and Signal Functions, Full Sample [1]

Clinical Cascade	Signal Functions	Clinical Cascades	Overestimated Readiness
(Signal Function)	**% Readiness, Tracer Items**	**% Readiness, Stage 2**	**[Signal Functions (-) Cascade]**
**Medical Treatments**			
**Manage Sepsis - Infection** *(Antibiotic)*	69.57%	47.83%	21.74%
**Manage Hemorrhage** *(Oxytocic)*	60.87%	60.87%	0.00%
**Manage Hypertensive Emergency** *(Anticonvulsant)*	78.26%	26.09%	52.17%
Mean Medical Readiness	69.57%	44.93%	24.64%
**Manual Procedures**			
**Manage Retained Placenta** *(Manual removal of retained placenta)*	69.57%	43.48%	26.09%
**Manage Incomplete Abortion** *(Manual removal of retained products of conception)*	69.57%	56.52%	13.05%
Mean Manual Readiness	69.57%	50.00%	19.57%
**Mean Overall Readiness:**	**69.57%**	**46.96%**	**22.61%**
	Signal Function Estimate	*Cascade Estimate*	*% Overestimated by Signal Functions*

[1] n = 23 facilities

For peer review only

Clinical Cascades

Overall Readiness

Across all five emergencies, the signal function model overestimated obstetric emergency readiness by 22.61% (**Table 1, Figure 1**). The mean signal function estimate of readiness was 69.57%; however, readiness measured by stage 2 of the cascade model was substantially lower at 46.96%. Notably, there was wide variability of overestimation by signal function, ranging from 0.00% (oxytocic) to 52.17% (anticonvulsant).

The 23% overestimation seen in the full sample remained largely consistent after stratifying by level of care, ownership, and country (**Table 2**). Among facilities with C-section capability, there was a 22.00% overestimation, compared to 23.08% among those without C-section capability. Likewise, there was a 22.11% overestimation among government facilities and a 20.00% overestimation among mission facilities. Similarly, there were little differences by country (22.35% overestimation in Kenya and 23.33% in Uganda).

Readiness by Emergency

When examining clinical cascade readiness estimates by disorder at the individual resource level, we saw a consistent pattern of readiness drop-off along the clinical cascade of care (from stage 1 to stage 3). The amount of readiness drop off at each stage and the specific resources driving the drop off differed by emergency (**Figures S1-S5**).

For peer review only

Table 2. Overestimation of Emergency Readiness Using Clinical Cascades and Signal Functions by Facility Characteristics

**Clinical Cascade** *(Signal Function)*	**Overestimated Readiness**						
	**[Signal Functions (-) Cascade]**						
		**C-Section Capacity**		**Ownership**		**Country**	
	**All**[1]	**C-section**[2]	**No C-section**[3]	**Government**[4]	**Mission**[5]	**Kenya**[6]	**Uganda**[7]
**Manage Sepsis - Infection** *(Antibiotic)*	21.74%	40.00%	7.70%	21.05%	25.00%	11.76%	50.00%
**Manage Hemorrhage** *(Oxytocic)*	0.00%	0.00%	0.00%	0.00%	0.00%	0.00%	0.00%
**Manage Hypertensive Emergency** *(Anticonvulsant)*	52.17%	60.00%	46.15%	47.37%	75.00%	52.94%	50.00%
**Manage Retained Placenta** *(Manual removal of retained placenta)*	26.09%	0.00%	46.15%	31.58%	0.00%	35.29%	0.00%
**Manage Incomplete Abortion** *(Manual removal of retained products of conception)*	13.05%	10.00%	15.39%	10.53%	0.00%	11.76%	16.67%
**Mean % Overestimated by Signal Functions**	**22.61%**	**22.00%**	**23.08%**	**22.11%**	**20.00%**	**22.35%**	**23.33%**

(1) n = 23 facilities
(2) n = 10 facilities
(3) n = 13 facilities
(4) n = 19 facilities
(5) n = 4 facilities
(6) n = 17 facilities
(7) n = 6 facilities

For peer review only

Readiness by Cascade

There were differences in readiness drop off along the stages of care from identification of the disorder through monitoring and modifying treatment (**Table 3**). These differences varied least for the sepsis cascade (SD = 5.02) and most for the hypertension cascade (SD = 35.41). There was also substantial variability in *when* the drop off in readiness was largest along the care cascade. For hypertensive emergency, the largest drop off occurred when identifying the emergency (69.57%). In contrast, there was no drop off in readiness in this stage for the hemorrhage, retained placenta, or incomplete abortion cascades. Of note, stage 1 of the hemorrhage and retained placenta cascades rely on staff skill alone, which was assumed to be 100% since it was not measured in this commodity-based study. Consequently, the 100% estimated skill for identification for these emergencies may overestimate actual readiness[21].

When examining drop off in readiness by level of care, ownership, and country, there were subtle differences. At facilities *with* C-section capability, the percentage of drop off across the stages of care varied least for sepsis (SD = 17.32) and most for hemorrhage (SD = 30.00, **Table S7**). Alternatively, among facilities *without* C-section capability, the drop off pattern was the same seen in the full sample – the percentage of drop off varied least for sepsis (SD = 11.75) and most for hypertensive emergencies (SD = 42.37).

When examining drop off by facility ownership, the drop off across stages varied least for sepsis across both types of facilities (SD = 5.26 and 14.43, respectively, **Table S8**). Drop off varied the most for hypertensive emergencies (SD = 35.04) among government facilities and hemorrhage (SD = 57.74) among mission facilities.

Across stages of care at Kenyan facilities, the percentage of drop off across stages varied least for sepsis (SD = 6.79) and most for hypertensive emergencies (SD = 42.55, **Table S9**). Across stages in Uganda, the percentage of drop off varied least for hypertensive emergencies (SD = 16.67) and most for hemorrhage (SD = 33.33).

For peer review only

Table 3. Mean Drop Off in Readiness by Cascade and Stage among All Facilities[1]

Readiness Drop Off by Stage				Readiness Drop Off by Cascade	
**Drop Off by Clinical Cascade**	**1**	**2**	**3**	***Mean Drop Off Across 3 Cascade Stages***	***SD***
	**Identify**	**Treat**	**Monitor-Modify**		
	--	--	--	**28.41%** [2]	**24.17** [3]
**Sepsis-Infection**	26.09%	26.09%	17.39%	23.19%	5.02
**Hemorrhage**	0.00%	39.13%	47.83%	28.99%	25.48
**Hypertensive Emergency**	69.57%	4.35%	13.04%	28.99%	35.41
**Retained Placenta**	0.00%	56.52%	34.78%	30.43%	28.51
**Incomplete Abortion**	0.00%	43.48%	47.83%	30.43%	26.45
**Overall Drop Off by Stage**					
Mean Drop Off Across Stage	**19.13%**	**33.91%**	**32.17%**		
SD	**30.37**	**19.78**	**16.44**	**3.01** [4]	

[1] n = 23 facilities
[2] Mean readiness drop off across 3 clinical cascade stages and 5 emergency cascades
[3] Mean of the standard deviations
[4] Standard deviation across 3 stages and 5 emergency cascades

For peer review only

Readiness Loss by Stage

This study revealed a consistent pattern of 28.41% drop off in readiness across emergencies and stages (SD = 3.01) despite moderate variability in where the drop off occurred across these stages (average SD across stages = 24.17, **Table 3**). Overall, the largest drop off took place in *treating* the disorder. The mean drop off across all five cascades was 19.13% for identification (stage 1), 33.91% for treatment (stage 2), and 32.17% for monitor and modify treatment (stage 3). **Figure S6** depicts the pattern of readiness drop off across clinical cascade stages and emergencies.

A similar pattern of drop off in readiness across level of care, facility ownership, and country emerged as well (**Table 4**). We found a consistent 25.67% drop off in readiness across emergencies and stages among facilities with C-sections (SD = 4.08) and 29.74% among those without (SD = 3.44). Consistent with the unstratified results, the largest drop off in readiness among facilities without C-section capability occurred in treating the disorder (43.08%) (**Table S7**). However, among facilities with C-section capability, the largest drop off occurred in the final stage of the cascade—monitoring and modifying therapy (38.00%).

By facility ownership, there was a consistent drop off in readiness of 29.12% across emergencies and stages for government hospitals (SD = 2.35). The drop-off was similar among mission hospitals (25.00%) but more variable (SD=10.21, **Table 4**). As in the full sample, the largest drop off among government facilities occurred in treating the disorder (37.89%). However, among the small sample of mission facilities, the largest drop off occurred in the final stage of the cascade—monitoring and modifying therapy (50.00%, **Table S8**).

By country, there was a consistent drop off in readiness of 27.84% across emergencies and stages among Kenyan facilities (SD = 4.02) and 30.00% among Ugandan facilities (SD = 3.04, **Table 4**). The largest drop off in readiness took place in *treating* the disorder among Kenyan facilities (35.29%) and in *monitoring and modifying* therapy among Ugandan facilities (40.00%, **Table S9**). However, these differences may be driven by the higher proportion of CEmOC facilities from Uganda in the sample.

Clinical Cascade Estimates of Readiness by Co-Factors

Although the overall patterns of readiness loss were consistent, two statistically significant differences in clinical cascade readiness were identified using stratified analyses ($p<0.05$). We found higher proportions of stage 2 readiness for the retained placenta cascade among facilities with reported C-section capability compared to those without (70.00% versus 23.08%, $p = 0.04$) and private/mission facilities compared to government facilities (100.00% versus 36.84%, $p = 0.02$).

For peer review only

14

Table 4. Mean Drop Off in Readiness by Facility Characteristics

Clinical Cascade	Mean Drop Off Across 3 Cascade Stages						
		C-section Capacity		Ownership		Country	
	All[1]	C-section[2]	No C-section[3]	Government[4]	Mission[5]	Kenya[6]	Uganda[7]
**Sepsis-Infection**	23.19%	20.00%	25.64%	26.32%	8.33%	21.57%	27.78%
**Hemorrhage**	28.99%	30.00%	28.21%	28.07%	33.33%	27.45%	33.33%
**Hypertensive Emergency**	28.99%	30.00%	28.21%	28.07%	33.33%	27.45%	33.33%
**Retained Placenta**	30.43%	26.67%	33.33%	31.58%	25.00%	31.37%	27.78%
**Incomplete Abortion**	30.43%	26.67%	33.33%	31.58%	25.00%	31.37%	27.78%
**Overall Drop Off**							
Mean[7]	**28.41%**	**26.67%**	**29.74%**	**29.12%**	**25.00%**	**27.84%**	**30.00%**
SD[8]	**3.01**	**4.08**	**3.44**	**2.35**	**10.21**	**4.02**	**3.04**

(1) n = 23 facilities
(2) n = 10 facilities
(3) n = 13 facilities
(4) n = 19 facilities
(5) n = 4 facilities
(6) n = 17 facilities
(7) n = 6 facilities
(7) Mean readiness drop off across 3 clinical cascade stages and 5 emergency cascades
(8) Standard deviation across 3 clinical cascade stages and 5 emergency cascades

For peer review only

DISCUSSION

This study expands the global scholarship on basic obstetric emergency readiness by confirming previously published patterns in overestimation by the signal functions and consistent patterns of resource drop off across the stages of care. It expands upon previous scholarship by highlighting emergency readiness estimates by level of care, facility ownership, and country. Results from this study aligned with those found in the single published comparison of the two methods: overestimations of obstetric emergency readiness, as calculated using signal functions, consistently occurs. Our mean overestimation across emergencies (22.61%) was about half of that reported in the formative study (54.48%)[21]. However, the formative study only included BEmOC facilities whereas this analysis included 43.48% CEmOC facilities. Despite some operational (e.g., variable inclusion/definitions) and contextual (e.g., country/region) differences, any overestimation of practical emergency readiness may be profoundly clinically relevant—particularly since elevated maternal mortality persists globally despite increased rates of facility-based deliveries. The 22.61% overestimation of readiness in this study—particularly since it includes CEmOC facilities—is profoundly concerning for advancing global maternal survival.

As was also the case in the two previous studies that examined clinical cascades as a measure of emergency readiness, we found a consistent drop off in readiness across the stages of care. Across stages and emergencies, a consistent drop off pattern of 28.41% (SD = 3.01) emerged in this study. The published study of 44 BEmOC facilities in Kenya found a consistent drop off pattern of 33.03% across the five emergencies and three stages of care[21]. As with our study, the formative study found little variation in this pattern (SD=0.41). Moreover, in a study of the clinical cascades as applied to neonatal emergencies, the mean drop off in readiness was 30.00% across emergencies and stages (based on 2016 data)[24]. Given that the 30% aggregate drop off for neonatal emergencies comes from the same facilities used in this maternal study, the mean readiness drop off indicator may be a system-level indicator of emergency readiness as suggested previously[21]. Together, these three studies suggest a consistent drop off in readiness of approximately 30% across emergencies and stages in both maternal and neonatal contexts across country contexts and levels of care. Together with the stage and condition-specific indicators, the aggregate drop off may be utilized to guide resource allocation and interventions and as a means of monitoring and comparing readiness across facilities, health systems, countries, or geographic regions. There is growing evidence that global multinational agencies, program planners, and policy makers should consider replacing the current SRI methods and the signal function estimates of readiness with the clinical cascades.

This is the first known study to measure how country, facility ownership, and level of care impact clinical cascade readiness. Overall estimates of readiness (according to both signal functions and clinical cascades) were higher among Ugandan than Kenyan facilities (73.33% vs. 68.24% according to the signal functions and 50.00% vs. 45.88% according to the clinical cascades). These results were expected given the level of care offered at the health facilities included in each country sample. Ugandan facilities were larger district or regional hospitals, while the Kenyan facilities were primarily smaller, subcounty hospitals and health centers. Likewise, estimates of readiness (based on both signal functions and cascades) were higher among mission facilities than government facilities, with nearly all mission facilities prepared to handle basic obstetric emergencies according to the signal functions (95.00%) and the majority prepared according to the clinical cascades (75.00%). Differences in readiness by level of care (among CEmOC versus BEmOC facilities) were evident as well. By definition, all facilities with C-section capability are CEmOC facilities that should be prepared to manage all BEmOC signal functions in addition to performing C-sections and blood transfusions. However, this study demonstrated that even CEmOC-designated facilities were not ready to perform all BEmOC functions. This gap was present when emergency readiness at CEmOC facilities was estimated using both the signal functions (80.00%) and clinical cascades (58.00%). This gap suggests that facilities offering CEmOC were not equipped to handle the complexity of obstetric emergencies as suggested by their designation. If seen across a larger

sample, the results could suggest the need for additional investment to equip CEmOC facilities with the supplies needed to handle the full range of obstetric emergencies.

This study has six primary limitations. First, there was a slight variation in the resources used to construct the signal functions and clinical cascades between this study and the two published studies, with changes made based on updated WHO guidelines at the time of this study and minor differences in operational definitions which varied by country context. Second, we used data from a single point in time. Given the unpredictable availability of consumable resources and drugs, estimating emergency readiness using a single time point may be insufficient to capture a facility or system's longitudinal readiness[18,28]. Collecting data from the same set of facilities at more frequent intervals could provide more accurate readiness data and reveal patterns in availability based on supply procurement. Third, due to the absence of select variables, we used proxies for missing tracer items. These proxies may not capture the nuances of actual resource availability. For instance, facilities without electricity often use kerosene-powered refrigerators. With information on neither refrigeration nor kerosene power availability, we used electricity alone, as a proxy for refrigeration needed to store select drugs. Facilities without electricity and using kerosene-powered refrigerators would be unintentionally reported as lacking refrigeration in our analyses. Fourth, more broadly, measuring the availability of resources alone does not take into account the quality of the resources nor clinicians' ability to use the resources effectively in clinical care[24,29]. Information on resource functionality (e.g., drug expiration dates), facility staffing levels, and clinician knowledge and skill would allow for a more precise estimates of practical emergency readiness at the facility level. However, many of these operational measures are difficult and costly to measure. Fifth, although resources were reported as present in the facilities, we could not determine how accessible resources were during emergencies. Given the importance of timing in an emergency, resource location and availability are critical and should be explored in future research. Sixth, future research should link cascade estimates of readiness to facilities' adverse clinical outcomes (e.g., maternal mortality ratio, maternal near miss, severe maternal outcomes, or prevalence of specific emergencies) to determine the empiric relationship between clinical cascade estimates of readiness and obstetric clinical outcomes.

Despite these limitations, this study offers important contributions to the emerging evidence on the use of clinical cascades as a more precise and targeted alternative to signal function estimates of emergency readiness. This conclusion adds to mounting concerns regarding the accuracy of the signal functions as an indicator of practical obstetric emergency readiness in the real world. By defining the resources necessary to identify and treat emergencies and monitor and modify treatment as clinically indicated, the cascades provide a detailed, step-wise analysis of resource availability and a novel set of readiness indicators. First, the cascades show precisely where the drop off in readiness occurs. Second, the drop off can be estimated at the stage of care, emergency, and individual resource level. Third, there is growing—albeit preliminary—evidence that the variability in drop off across stages and cascades may provide an indicator of emergency readiness at the system level. Results from this study may inform strategies that optimize emergency commodity provision for Kenyan and Ugandan facilities and strengthen system-level strategies for improving emergency-specific maternal survival globally. While measuring readiness according to the clinical cascades model requires a marginal increase in effort during data collection, this approach provides a more nuanced picture of capacity to manage obstetric emergencies. Further investigation into diverse contexts (e.g., additional countries, regions, levels of care) is warranted and could further support existing calls to shift away from standard readiness metrics (such as the signal functions and SRI) toward the more nuanced, precise, and practical estimates offered by the clinical cascades.

CONCLUSIONS

In conclusion, emergency care is critical in managing the obstetric complications driving elevated maternal mortality ratios in Sub-Saharan Africa. The prominent role emergency care plays in the obstetric

For peer review only

For peer review only - http://bmjopen.bmj.com/site/about/guidelines.xhtml

experience mandates an accurate measurement of readiness to handle such complications. This study suggests the need to reconsider the signal functions as the preferred method of measuring readiness, which aligns with the conclusions made in published research. The clinical cascades provide a more nuanced picture of clinical care that can be used to optimize emergency commodity provision and strengthen system-level strategies for improving emergency-specific maternal survival.

Contributors: All authors listed are responsible for this study and have participated in the concept and design, analysis, and interpretation of data, drafting and revising of the manuscript. DW, EB, AW, and PW conceptualized, secured funding, and oversaw data collection for the parent study. BW and JNC conceptualized the nested analysis. BW analyzed the data and wrote the first draft of the submitted manuscript, with assistance from JNC and JMS. All authors provided feedback and commented on the manuscript.

Acknowledgements:
We thank Damien Scogin for his graphic design and Cecilia Roach for her copy-editing contributions. We appreciate all the facility healthcare workers, leadership and administration who made this work possible. We thank all members of the Preterm Birth Initiative Kenya & Uganda Implementation Research Collaborative, particularly the data teams who helped strengthen and collect data from facility maternity registers for the parent PTBi study. We are grateful to the facility clinicians and study participants for their valuable contribution to the parent PTBi study and by extension this nested analysis. Furthermore, we thank the research teams at UCSF, Makerere University, Kenya Medical Research Institute, and Emory University for their hard work, collaboration, and ongoing commitment to maternal survival.

For peer review only

REFERENCES

1 World Health Organization. Maternal mortality, 2019. Available: https://www.who.int/news-room/fact-sheets/detail/maternal-mortality [Accessed Mar 21 2021].

2 World Health Organization. Trends in maternal mortality 2000 to 2017: estimates by WHO, UNICEF, UNFPA, World Bank Group, and the United Nations Population Division. Geneva: Wrold Health Organization; 2019.

3 Say L, Chou D, Gemmill A, et al. Global causes of maternal death: a WHO systematic analysis. *Lancet Glob Health*. 2014;2:e323-33. doi:10.1016/S2214-109X(14)70227-X

4 World Health Organization. Mother-baby package: implementing safe motherhood in countries. Geneva, Switzerland: Maternal Health and Safe Motherhood Programme, Division of Family Health, 1996.

5 World Health Organization. Monitoring emergency obstetric care: a handbook. Geneva, Switzerland: Department of Reproductive Health and Research, 2009.

6 Nesbitt RC, Lohela TJ, Manu A, et al. Correction: quality along the continuum: a health facility assessment of intrapartum and postnatal care in Ghana. *PLoS One*. 2015;10:e0141517. doi:10.1371/journal.pone.0141517

7 Paxton A, Bailey P, Lobis S. The United Nations process indicators for emergency obstetric care: reflections based on a decade of experience. *Obstet Gynecol Int J*. 2006;95:192-208. doi:10.1016/j.ijgo.2006.08.009

8 World Health Organization. Monitoring the building blocks of health systems: a handbook of indicators and their measurement strategies. Geneva, Switzerland: World Health Organization, 2010.

9 Gabrysch S, Civitelli G, Edmond KM, et al. New signal functions to measure the ability of health facilities to provide routine and emergency newborn care. *PLoS Med*. 2012;9:e1001340. doi:10.1371/journal.pmed.1001340

10 Nickerson JW, Adams O, Attaran A, et al. Monitoring the ability to deliver care in low- and middle-income countries: a systematic review of health facility assessment tools. *Health Policy Plan*. 2015;30:675-86. doi:10.1093/heapol/czu043

11 Gill Z, Bailey P, Waxman R, et al. A tool for assessing 'readiness' in emergency obstetric care: The room-by-room 'walk-through'. *Obstet Gynecol Int J*. 2005;89:191-9. doi:10.1016/j.ijgo.2004.12.043

12 Government of Sierra Leone. Sierra Leone service availability and readiness assessment 2011 report. Ministry of Health and Sanitation, 2011.

13 Government of Kenya. Kenya service availability and assessment mapping (SARAM) report 2013. Nairobi, Kenya: Ministry of Health, 2013.

14 O'Neill K, Takane M, Sheffel A, et al. Monitoring service delivery for universal health coverage: the Service Availability and Readiness Assessment. *Bull World Health Organ*. 2013;91:923-31. doi:10.2471/BLT.12.116798

15 World Health Organization. Service availability and readiness assessment (SARA): an annual monitoring system for service delivery. Geneva, Switzerland: World Health Organization, 2015.

16 Spangler SA. Assessing skilled birth attendants and emergency obstetric care in rural Tanzania: the inadequacy of using global standards and indicators to measure local realities. *Reprod Health Matters*. 2012;20:133-41. doi:10.1016/S0968-8080(12)39603-4

17 Brenner S, De Allegri M, Gabrysch S, et al. The quality of clinical maternal and neonatal healthcare – A strategy for identifying 'routine care signal functions'. *PLoS One*. 2015;10:e0123968. doi:10.1371/journal.pone.0123968

18 Bailey P, Paxton A, Lobis S, et al. The availability of life-saving obstetric services in developing countries: An in-depth look at the signal functions for emergency obstetric care. *Obstet Gynecol Int*. 2006;93:285-91. doi:10.1016/j.ijgo.2006.01.028

For peer review only

19 Collender G, Gabrysch S, Campbell OM. Reducing maternal mortality: better monitoring, indicators and benchmarks needed to improve emergency obstetric care. Research summary for policymakers. *Trop Med Int Health*. 2012;17:694-6. doi:10.1111/j.1365-3156.2012.02983.x

20 Gabrysch S, Zanger P, Seneviratne HR, et al. Tracking progress towards safe motherhood: meeting the benchmark yet missing the goal? An appeal for better use of health-system output indicators with evidence from Zambia and Sri Lanka. *Trop Med Int Health*. 2011;16:627-39. doi:10.1111/j.1365-3156.2011.02741.x

21 Cranmer JN, Dettinger J, Calkins K, et al. Beyond signal functions in global obstetric care: Using a clinical cascade to measure emergency obstetric readiness. PLoS One. 2018;13:e0184252. doi:10.1371/journal.pone.0184252

22 Potter C, Brough R. Systemic capacity building: a hierarchy of needs. *Health Policy Plan*. 2004;19:336-45. doi:10.1093/heapol/czh038

23 Gardner EM, McLees MP, Steiner JF, et al. The spectrum of engagement in HIV care and its relevance to test-and-treat strategies for prevention of HIV infection. *Clin Infect Dis*. 2011;52:793-800. doi:10.1093/cid/ciq243

24 Morgan MC, Spindler H, Nambuya H, et al. Clinical cascades as a novel way to assess physical readiness of facilities for the care of small and sick neonates in Kenya and Uganda. *PLoS One*. 2018;13:e0207156. doi:10.1371/journal.pone.0207156

25 Otieno P, Waiswa P, Butrick E, et al. Strengthening intrapartum and immediate newborn care to reduce morbidity and mortality of preterm infants born in health facilities in Migori County, Kenya and Busoga Region, Uganda: a study protocol for a randomized controlled trial. *Trials*. 2018;19:313. doi:10.1186/s13063-018-2696-2

26 The Partnership for Maternal, Newborn & Child Health.. Essential interventions, commodities and guidelines for reproductive, maternal, newborn and child health. Geneva, Switzerland: The Partnership for Maternal, Newborn & Child Health.. Essential interventions, commodities and guidelines for reproductive, maternal, newborn and child health., 2011.

27 World Health Organization. Pregnancy, childbirth, postpartum and newborn care: A guide for essential practice. 3rd ed. Geneva, Switzerland: World Health Organization, 2015.

28 Paxton A, Bailey P, Lobis S, et al. Global patterns in availability of emergency obstetric care. *Int J Gynaecol Obstet*. 2006;93:300-7. doi:10.1016/j.ijgo.2006.01.030

29 Carter R, Xiong X, Lusamba-Dikassa PS, et al. Facility conditions, obstetric and neonatal care practices, and availability of emergency obstetric and neonatal care in 72 rural health facilities in the Democratic Republic of the Congo: A cross-sectional study. *Gates Open Res*. 2019;3:13. doi:10.12688/gatesopenres.12905.2

30 World Health Organization. Health statistics and information systems: maternal mortality ratio (per 100,000 live births), 2020. Available: https://www.who.int/healthinfo/statistics/indmaternalmortality/en/.

31 Harvard School of Medicine. Abortion (Termination of Pregnancy), 2019. Available: https://www.health.harvard.edu/medical-tests-and-procedures/abortion-termination-of-pregnancy-a-to-z.

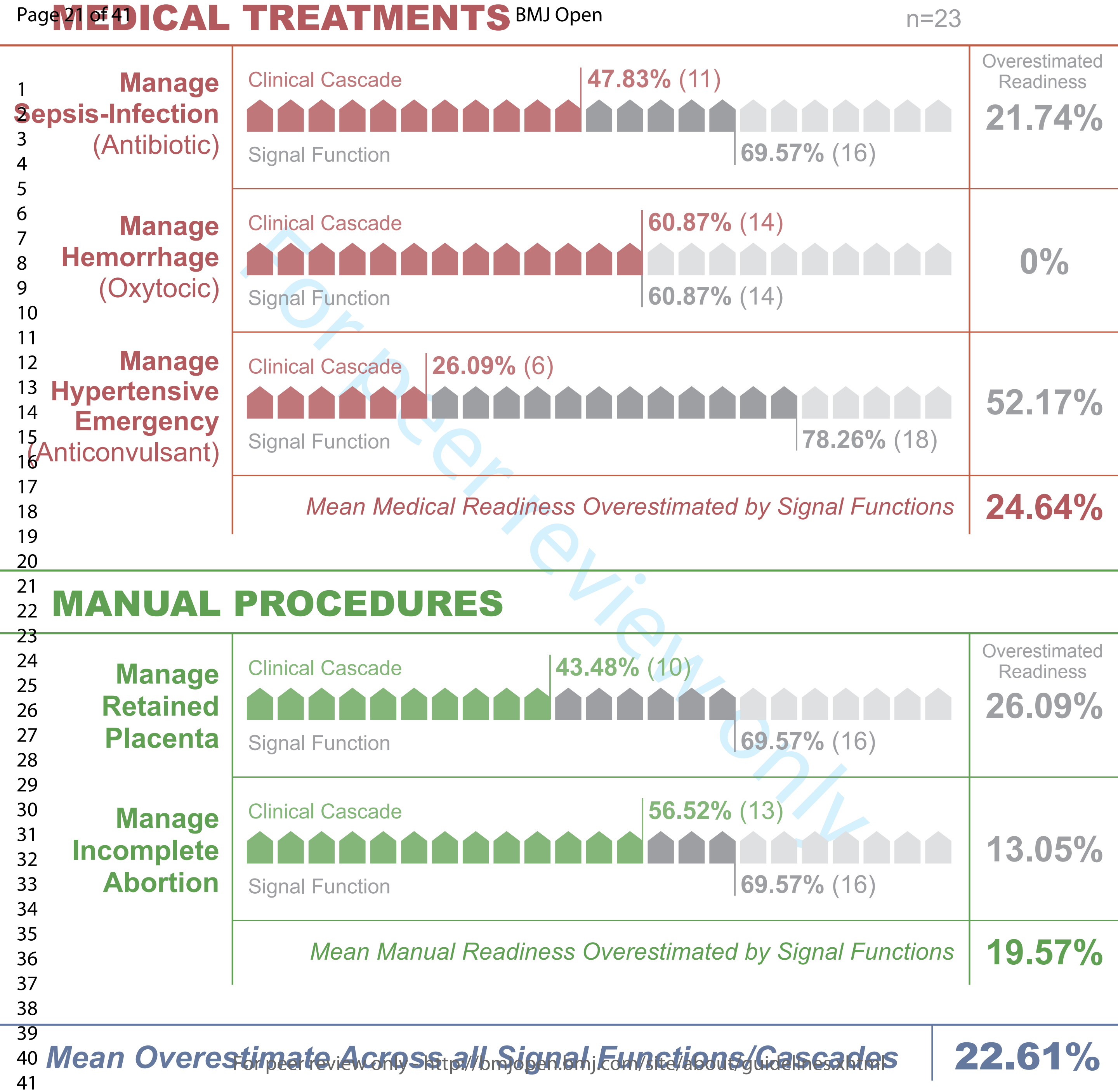
MEDICAL TREATMENTS
n=23
Manage Sepsis-Infection (Antibiotic)
Clinical Cascade
47.83% (11)
Signal Function
69.57% (16)
Overestimated Readiness
21.74%
Manage Hemorrhage (Oxytocic)
Clinical Cascade
60.87% (14)
Signal Function
60.87% (14)
0%
Manage Hypertensive Emergency (Anticonvulsant)
Clinical Cascade
26.09% (6)
Signal Function
78.26% (18)
52.17%
Mean Medical Readiness Overestimated by Signal Functions
24.64%
MANUAL PROCEDURES
Manage Retained Placenta
Clinical Cascade
43.48% (10)
Signal Function
69.57% (16)
Overestimated Readiness
26.09%
Manage Incomplete Abortion
Clinical Cascade
56.52% (13)
Signal Function
69.57% (16)
13.05%
Mean Manual Readiness Overestimated by Signal Functions
19.57%
Mean Overestimate Across all Signal Functions/Cascades
22.61%

SUPPLEMENTAL MATERIAL

Definition of Terms

- **Maternal deaths:** The death of a woman during pregnancy or within 42 days of termination of the pregnancy, regardless of the duration and site of the pregnancy, from any cause related to or exacerbated by the pregnancy or its management, excluding accidental or incidental causes[30]
- **Live birth:** the complete expulsion or extraction of a product of conception from its mother, irrespective of pregnancy duration, which, after separation, breathes or shows other signs of life[30]
- **Maternal Mortality Ratio (MMR):** Maternal deaths per 100,000 live births[30]
- **Hemorrhage:** Severe bleeding before (antepartum hemorrhage) or after (postpartum hemorrhage) labor[5]
- **Sepsis:** A complication caused by infection, resulting in high temperatures (38° C or higher) and one or more of the following symptoms: lower abdominal pain, abnormal vaginal discharge, and tender or poorly contracted uterus[5]
- **Preeclampsia:** A sudden spike in maternal blood pressure accompanied by protein in the urine, which can progress to a more serious eclampsia[5]
- **Eclampsia:** A blood-pressure related disorder that can result in maternal seizures which, untreated, can progress to coma or death[5]
- **Prolonged or obstructed labor:** Occurs when the first stage of labor lasts longer than 12 hours, the second stage of labor lasts longer than one hour, or labor is obstructed by uterine scarring or fetal malpresentation (fetus side (transverse), brow, or face presenting in the uterine isthmus instead of the head)[5].
- **Abortion:** The termination of a pregnancy through the removal of pregnancy tissue, products of conception, or the fetus and placenta from the uterus[31]. Complications of a spontaneous or induced abortion, especially an induced abortion performed in non-medical/unsterile settings, can result in hemorrhage, sepsis, or other complications[5]
- **Signal functions:** Six clinical actions, three medical and three manual procedures, used during obstetric emergencies[5]
- **Clinical cascades:** A clinically-oriented approach to measuring facility readiness using a step-wise cascading relationship between emergency resources[21,24]
- **Emergency Obstetric Care (EmOC):** Clinical processes needed to rapidly manage or stabilize a patient whose life is in imminent danger during pregnancy[17]
- **Basic Emergency Obstetric Care (BEmOC):** The ability to perform six maternal signal functions: (1) administer parenteral antibiotics, (2) administer uterotonic drugs, (3) administer parenteral anticonvulsants for pre-eclampsia and eclampsia, (4) manually remove the placenta, (5) remove retained products of conception, and (6) perform assisted vaginal delivery[5]
- **Comprehensive Emergency Obstetric Care (CEmOC):** The ability to perform the six basic signal functions as well as surgery and blood transfusion[5]
- **Drop off in readiness:** The decrease in the percentage of facilities with the resources necessary to manage basic obstetric emergencies.
- **Mission facilities:** Facilities across both Kenya and Uganda that are neither owned nor operated by the government.

For peer review only

Table S1: Items that Define Signal Functions and Clinical Cascades

Obstetric Emergency	Signal Function	Signal Function Tracer Item	Clinical Cascade Items by Stage		
			Stage 1: Identify	Stage 2: Treat	Stage 3: Monitor-Modify
Manage Sepsis-Infection	Administer Parenteral Antibiotics	IV Fluid (1)<br>IV Cannula/Catheter<br>IV Tubing<br>Ampicillin or Penicillin<br>Gentamicin or Cefotaxime or Ceftriaxone	Thermometer<br>Stethoscope<br>Sphygmomanometer	IV Tubing<br>IV Cannula/Catheter<br>IV Fluid (1)<br>IV Pole<br>Ampicillin or Penicillin<br>Gentamicin or Cefotaxime or Ceftriaxone	Protocol: Sepsis (2)
Manage Hemorrhage	Administer Parenteral Oxytocics	Gloves (3)<br>IV Fluid (1)<br>IV Cannula/Catheter<br>IV Tubing<br>Oxytocin or Misoprostol or Ergometrine	Staff skill (4)	Gloves (3)<br>IV Tubing<br>IV Cannula/Catheter<br>IV Fluid (1)<br>IV Pole<br>Refrigeration (5)<br>Oxytocin	Sphygmomanometer<br>Stethoscope<br>Misoprostol or Ergometrine<br>Urinary Catheter<br>Oxygen Source (6)<br>Oxygen Tubing<br>Oxygen Delivery (7)<br>Protocol: Hemorrhage
Manage Hypertensive Emergencies	Administer Parenteral Anticonvulsant	IV Fluid (1)<br>IV Cannula/Catheter<br>IV Tubing	Sphygmomanometer<br>Stethoscope<br>Urine Cup	IV Tubing<br>IV Cannula/Catheter<br>IV Fluid (1)	Urinary Catheter<br>Calcium Gluconate<br>Oxygen Source (6)

For peer review only

		Magnesium Sulfate	Urine Dipstick	IV Pole<br>Magnesium Sulfate<br>Hydralazine or Labetalol or Nifedipine or Methyldopa	Oxygen Tubing<br>Oxygen Delivery [7]<br>Protocol: Eclampsia
Manage Retained Placenta	Perform Manual Removal of Retained Placenta	Flashlight or power<br>IV Fluid [1]<br>IV Cannula/Catheter<br>IV Tubing<br>Oxytocin<br>Ampicillin or Penicillin or Gentamicin or Cefotaxime or Ceftriaxone	Staff Skill [4]<br>Light Source [8]	Gloves [3]<br>IV Tubing<br>IV Cannula/Catheter<br>IV Fluid [1]<br>IV Pole<br>Refrigeration [5]<br>Oxytocin<br>Diazepam<br>Ampicillin or Penicillin	Sphygmomanometer<br>Stethoscope<br>Misoprostol or Ergometrine<br>Gentamicin or Cefotaxime or Ceftriaxone<br>Urinary Catheter<br>Protocol(s) [9]
Manage Incomplete Abortion	Perform Manual Removal of Retained Products of Conception	Flashlight or power<br>MVA Kit<br>IV Fluid [1]<br>IV Cannula/Catheter<br>IV Tubing	Speculum<br>Light Source [8]	Gloves [3]<br>IV Tubing<br>IV Cannula/Catheter<br>IV Fluid [1]<br>Manual Vacuum Aspirator Kit [10]	Sphygmomanometer<br>Stethoscope<br>Refrigeration [5]<br>Misoprostol or Ergometrine<br>Gentamicin or Cefotaxime or Ceftriaxone

For peer review only

		Ampicillin or Penicillin or Gentamicin or Cefotaxime or Ceftriaxone		IV Pole<br><br>Lidocaine<br>Ampicillin or Penicillin	Urinary Catheter<br><br>Protocol(s) [(11)]

[(1)] Either normal saline (NS) or lactated ringer's (LR)

[(2)] Puerperal sepsis, infection, or antibiotic administration Protocol

[(3)] Sterile gloves or clean disposable latex gloves (represented as a single variable in the original dataset)

[(4)] 100% staff skill for identifying the emergency was assumed

[(5)] Power used as proxy for refrigeration

[(6)] Oxygen concentrator and power or oxygen cylinder and oxygen head

[(7)] Oxygen mask or nasal cannula

[(8)] Flashlight or power (as proxy for electric lights)

[(9)] Retained placenta protocol or hemorrhage protocol or infection protocol

[(10)] Manual vacuum device and cannula

[(11)] Incomplete abortion protocol or hemorrhage protocol or infection protocol

For peer review only

Table S2. Facility Demographics

	**%**	**n** [1]
**Country**		
Kenya	73.91%	17
Uganda	26.09%	6
**Level (ownership)**		
Mission	17.39%	4
Government	82.61%	19
Regional Referral Hospital	*4.35%*	*1*
District Hospital	*13.04%*	*3*
County Referral Hospital	*4.35%*	*1*
Sub-County Hospital	*43.48%*	*10*
Health Center	*17.39%*	*4*
**C-Section Capability**		
Yes	43.48%	10
No	56.52%	13

(1) n = 23

For peer review only

Table S3. Annual Delivery Volume

	Annual Total [1]	Median [2]	25th	75th	IQR
**Kenya** [3]	15,858.49	598.91	495.27	999.27	504.00
**Uganda** [4]	21,591.27	2,662.50	2,059.25	5,395.95	3,336.70
Total [5]	*37,449.76*	*994.50*	*537.27*	*1,808.64*	*1,271.36*

[1] Data was provided for up to 11 months in Kenya and up to 12 months in Uganda. To calculate the total annual delivery volume, we calculate the monthly average using the number of reported months for each individual facility and then multiplied that number by 12.
[2] The median and IQR are reported instead of the mean and standard deviation as the data are not normally distributed.
[3] n = 17
[4] n = 6
[5] n = 23

For peer review only

Table S4. Comparison of Emergency Readiness Using Clinical Cascades and Signal Functions by Reported C-Section Capability

	**Signal Functions**		**Clinical Cascades**		**Overestimated Readiness**	
	**% Readiness, Tracer Items**		**% Readiness, Stage 2**		**[Signal Functions (-) Cascade]**	
**Clinical Cascade** *(Signal Function)*	**C-section** [(1)]	**No C-section** [(2)]	**C-section**	**No C-section**	**C-section**	**No C-section**
**Medical Treatments**						
**Manage Sepsis - Infection** *(Antibiotic)*	90.00%	53.85%	50.00%	46.15%	40.00%	7.70%
**Manage Hemorrhage** *(Oxytocic)*	70.00%	53.85%	70.00%	53.85%	0.00%	0.00%
**Manage Hypertensive Emergency** *(Anticonvulsant)*	90.00%	69.23%	30.00%	23.08%	60.00%	46.15%
Mean Medical Readiness	83.33%	58.98%	50.00%	41.03%	33.33%	17.95%
**Manual Procedures**						
**Manage Retained Placenta** *(Manual removal of retained placenta)*	70.00%	69.23%	70.00%	23.08%	0.00%	46.15%
**Manage Incomplete Abortion** *(Manual removal of retained products of conception)*	80.00%	61.54%	70.00%	46.15%	10.00%	15.39%
Mean Manual Readiness	75.00%	65.39%	70.00%	34.62%	5.00%	30.77%
**Mean Overall Readiness:**	**80.00%**	**61.54%**	**58.00%**	**38.46%**	**22.00%**	**23.08%**
	Signal Function Estimate		*Cascade Estimate*		*% Overestimated by Signal Functions*	

(1) n = 10 facilities
(2) n = 13 facilities

For peer review only

Table S5. Comparison of Emergency Readiness Using Clinical Cascades and Signal Functions by Ownership

Readiness Estimates by Model and Ownership						
	**Signal Functions**		**Clinical Cascades**		**Overestimated Readiness**	
	**% Readiness, Tracer Items**		**% Readiness, Stage 2**		**[Signal Functions (-) Cascade]**	
**Clinical Cascade** *(Signal Function)*	**Gov.** [(1)]	**Mission** [(2)]	**Gov.**	**Mission**	**Gov.**	**Mission**
**Medical Treatments**						
**Manage Sepsis - Infection** *(Antibiotic)*	63.16%	100.00%	42.11%	75.00%	21.05%	25.00%
**Manage Hemorrhage** *(Oxytocic)*	57.89%	100.00%	57.89%	100.00%	0.00%	0.00%
**Manage Hypertensive Emergency** *(Anticonvulsant)*	73.68%	100.00%	26.32%	25.00%	47.37%	75.00%
Mean Medical Readiness	64.91%	100.00%	42.11%	66.67%	22.81%	33.33%
**Manual Procedures**						
**Manage Retained Placenta** *(Manual removal of retained placenta)*	68.42%	100.00%	36.84%	100.00%	31.58%	0.00%
**Manage Incomplete Abortion** *(Manual removal of retained products of conception)*	63.16%	75.00%	52.63%	75.00%	10.53%	0.00%
Mean Manual Readiness	65.79%	87.50%	44.74%	87.50%	21.05%	0.00%
**Mean Overall Readiness:**	**65.26%**	**95.00%**	**43.16%**	**75.00%**	**22.11%**	**20.00%**
	Signal Function Estimate		*Cascade Estimate*		*% Overestimated by Signal Functions*	

[(1)] n = 19 facilities
[(2)] n = 4 facilities

For peer review only

Table S6. Comparison of Emergency Readiness Using Clinical Cascades and Signal Functions by Country

	**Signal Functions**		**Clinical Cascades**		**Overestimated Readiness**	
	**% Readiness, Tracer Items**		**% Readiness, Stage 2**		**[Signal Functions (-) Cascade]**	
**Clinical Cascade** *(Signal Function)*	**Kenya**[(1)]	**Uganda**[(2)]	**Kenya**	**Uganda**	**Kenya**	**Uganda**
**Medical Treatments**						
**Manage Sepsis – Infection** *(Antibiotic)*	64.71%	83.33%	52.94%	33.33%	11.76%	50.00%
**Manage Hemorrhage** *(Oxytocic)*	58.82%	66.67%	58.82%	66.67%	0.00%	0.00%
**Manage Hypertensive Emergency** *(Anticonvulsant)*	76.47%	83.33%	23.53%	33.33%	52.94%	50.00%
Mean Medical Readiness	66.67%	77.78%	45.10%	44.44%	21.57%	33.33%
**Manual Procedures**						
**Manage Retained Placenta** *(Manual removal of retained placenta)*	70.59%	66.67%	35.29%	66.67%	35.29%	0.00%
**Manage Incomplete Abortion** *(Manual removal of retained products of conception)*	70.59%	66.67%	58.82%	50.00%	11.76%	16.67%
Mean Manual Readiness	70.59%	66.67%	47.06%	58.33%	23.53%	8.33%
**Mean Overall Readiness:**	**68.24%**	**73.33%**	**45.88%**	**50.00%**	**22.35%**	**23.33%**
	Signal Function Estimate		*Cascade Estimate*		*% Overestimated by Signal Functions*	

[(1)] n = 17 facilities
[(2)] n = 6 facilities

For peer review only

Table S7. Mean Readiness Loss by Cascade and Stage by Reported C-Section Capability

**Readiness Loss by Stage**							**Readiness Loss by Cascade**			
**Loss by Clinical Cascade**	**1**		**2**		**3**		***Mean Loss Across 3 Cascade Stages***		***SD***	
	**Identify**		**Treat**		**Monitor-Modify**					
**Reported C-Section Capability**	**Yes**(1)	**No**(2)	**Yes**	**No**	**Yes**	**No**	**Yes**	**No**	**Yes**	**No**
	--		--		--		**26.67%**(3)	**29.74%**	24.82(4)	29.49
**Sepsis-Infection**	40.00%	15.38%	10.00%	38.46%	10.00%	23.08%	20.00%	25.64%	17.32	11.75
**Hemorrhage**	0.00%	0.00%	30.00%	46.15%	60.00%	38.46%	30.00%	28.21%	30.00	24.73
**Hypertensive Emergency**	60.00%	76.92%	10.00%	0.00%	20.00%	7.69%	30.00%	28.21%	26.46	42.37
**Retained Placenta**	0.00%	0.00%	30.00%	76.92%	50.00%	23.08%	26.67%	33.33%	25.17	39.47
**Incomplete Abortion**	0.00%	0.00%	30.00%	53.85%	50.00%	46.15%	26.67%	33.33%	25.17	29.12
**Overall Loss by Stage**										
Mean Loss Across Stage	**20.00%**	**18.46%**	**22.00%**	**43.08%**	**38.00%**	**27.69%**				
SD	28.28	33.35	10.95	28.05	21.68	15.00	4.08(5)	3.44		

(1) n = 10 facilities
(2) n = 13 facilities
(3) Mean readiness loss across 3 clinical cascade stages and 5 emergency cascades
(4) Mean of the standard deviations
(5) Standard deviation across 3 stages and 5 emergency cascades

For peer review only

Table S8. Mean Readiness Loss by Cascade and Stage by Ownership

Readiness Loss by Stage							Readiness Loss by Cascade			
**Loss by Clinical Cascade**	**1**		**2**		**3**		***Mean Loss Across 3 Cascade Stages***		***SD***	
	**Identify**		**Treat**		**Monitor-Modify**					
**Ownership**	**Gov.** [1]	**Mission** [2]	**Gov.**	**Mission**	**Gov.**	**Mission**	**Gov.**	**Mission**	**Gov.**	**Mission**
	--		--		--		**29.12%**[3]	**25.00%**	**24.71**[4]	**35.73**
**Sepsis-Infection**	26.32%	25.00%	31.58%	0.00%	21.05%	0.00%	26.32%	8.33%	5.26	14.43
**Hemorrhage**	0.00%	0.00%	42.11%	0.00%	42.11%	100.00%	28.07%	33.33%	24.31	57.74
**Hypertensive Emergency**	68.42%	75.00%	5.26%	0.00%	10.53%	25.00%	28.07%	33.33%	35.04	38.19
**Retained Placenta**	0.00%	0.00%	63.16%	0.00%	31.58%	75.00%	31.58%	25.00%	31.58	43.30
**Incomplete Abortion**	0.00%	0.00%	47.37%	25.00%	47.37%	50.00%	31.58%	25.00%	27.35	25.00
**Overall Loss by Stage**										
Mean Loss Across Stage	**18.95%**	**20.00%**	**37.89%**	**5.00%**	**30.53%**	**50.00%**				
SD	**29.91**	**32.60**	**21.51**	**11.18**	**15.07**	**39.53**	**2.35**[5]	**10.21**		

[1] n = 19 facilities

[2] n = 4 facilities

[3] Mean readiness loss across 3 clinical cascade stages and 5 emergency cascades

[4] Mean of the standard deviations

[5] Standard deviation across 3 stages and 5 emergency cascades

For peer review only

Table S9. Mean Readiness Loss by Cascade and Stage by Country

Readiness Loss by Stage							Readiness Loss by Cascade			
**Loss by Clinical Cascade**	**1**		**2**		**3**		***Mean Loss Across 3 Cascade Stages***		***SD***	
	**Identify**		**Treat**		**Monitor-Modify**					
**Country**	**Kenya**[(1)]	**Uganda**[(2)]	**Kenya**	**Uganda**	**Kenya**	**Uganda**	**Kenya**	**Uganda**	**Kenya**	**Uganda**
	--		--		--		**27.84%**[(3)]	**30.00%**	26.66[(4)]	24.03
**Sepsis-Infection**	17.65%	50.00%	29.41%	16.67%	17.65%	16.67%	21.57%	27.78%	6.79	19.25
**Hemorrhage**	0.00%	0.00%	41.18%	33.33%	41.18%	66.67%	27.45%	33.33%	23.77	33.33
**Hypertensive Emergency**	76.47%	50.00%	0.00%	16.67%	5.88%	33.33%	27.45%	33.33%	42.55	16.67
**Retained Placenta**	0.00%	0.00%	64.71%	33.33%	29.41%	50.00%	31.37%	27.78%	32.40	25.46
**Incomplete Abortion**	0.00%	0.00%	41.18%	50.00%	52.94%	33.33%	31.37%	27.78%	27.80	25.46
**Overall Loss by Stage**										
Mean Loss Across Stage	**18.82%**	**20.00%**	**35.29%**	**30.00%**	**29.41%**	**40.00%**				
SD	**33.12**	**27.39**	**23.53**	**13.94**	**18.60**	**19.00**	**4.02**[(5)]	**3.04**		

(1) n = 17 facilities
(2) n = 6 facilities
(3) Mean readiness loss across 3 clinical cascade stages and 5 emergency cascades
(4) Mean of the standard deviations
(5) Standard deviation across 3 stages and 5 emergency cascades

For peer review only

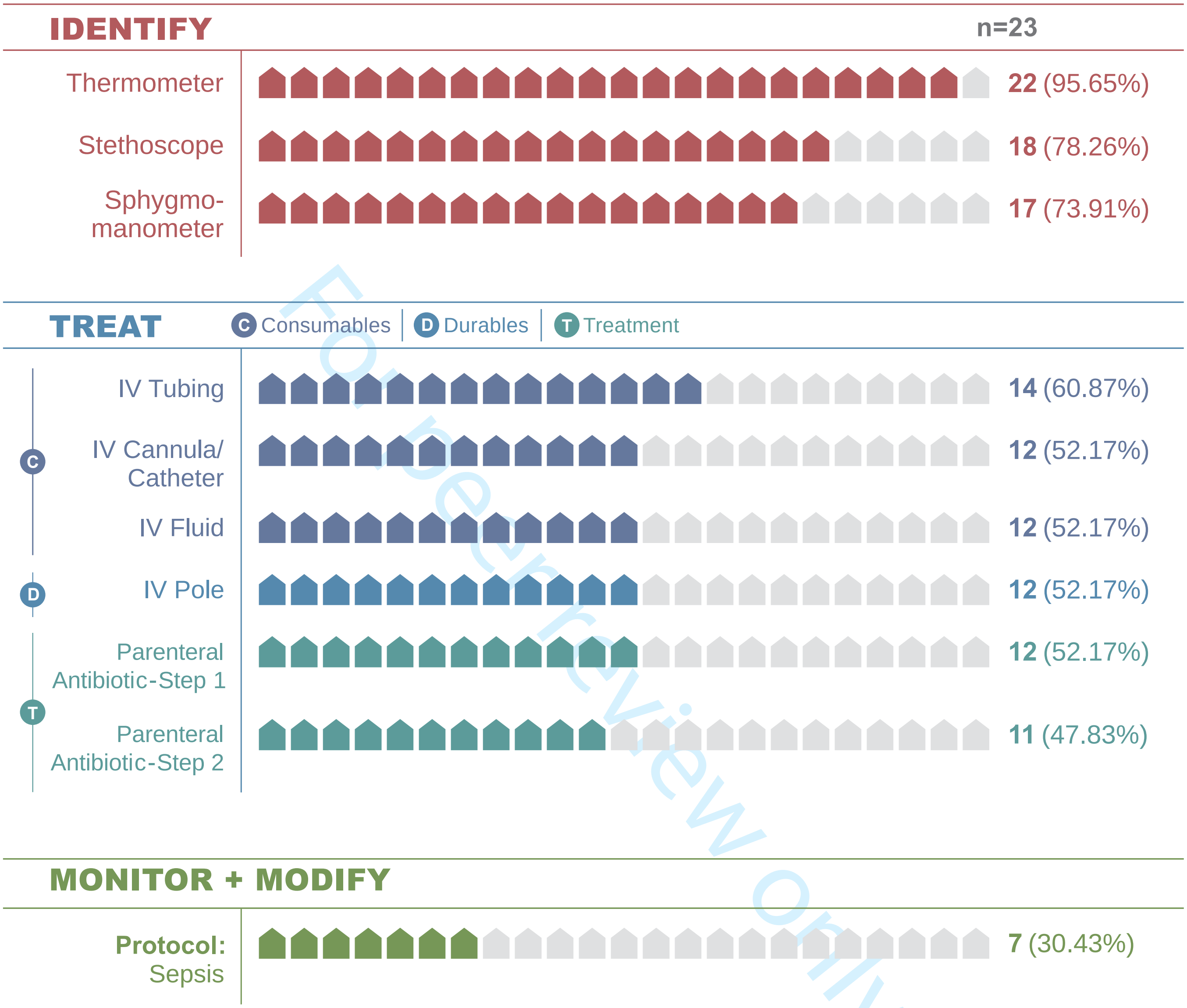

Figure S1. Sepsis Clinical Cascade

For peer review only

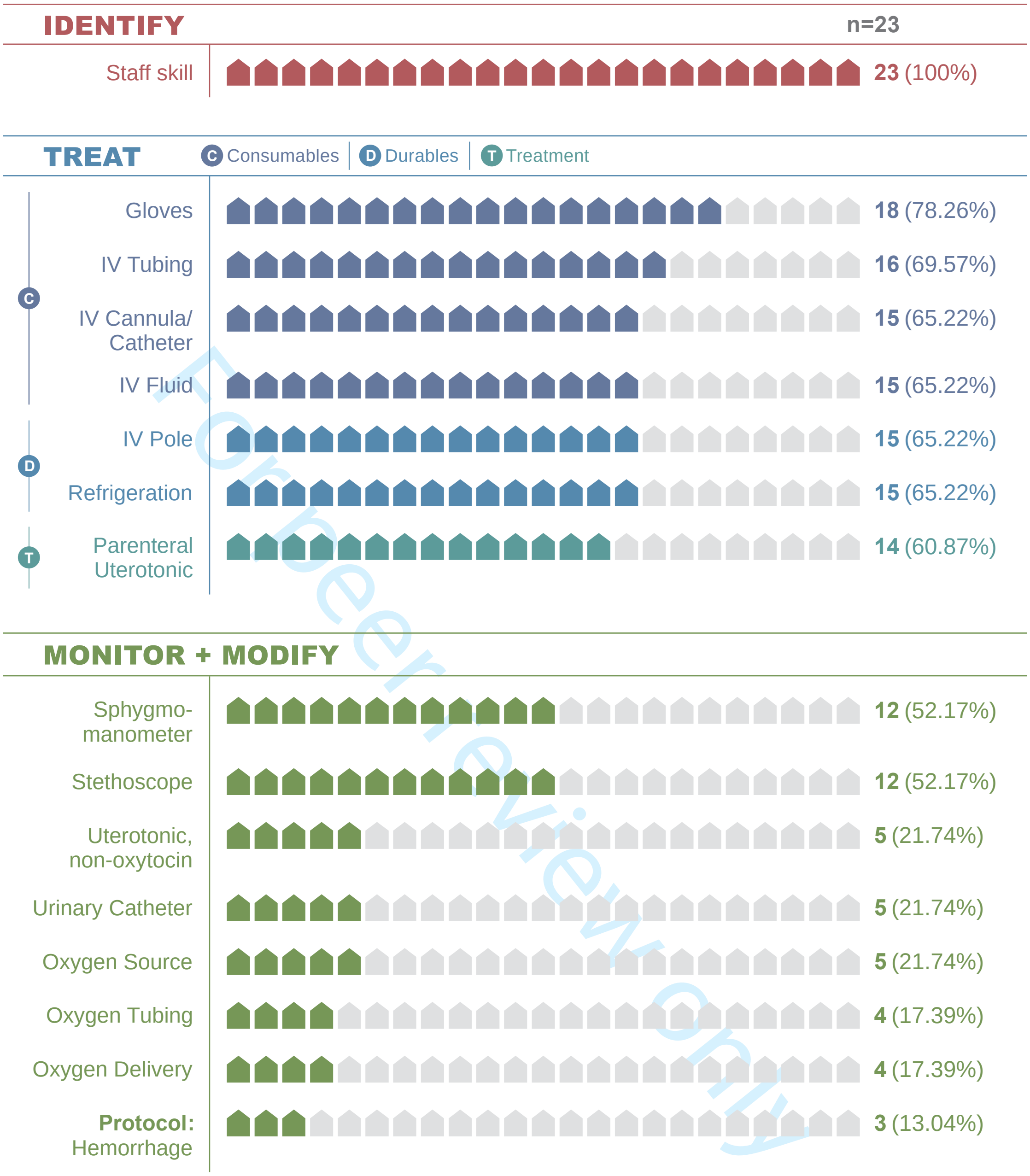

Figure S2. Hemorrhage Clinical Cascade

For peer review only

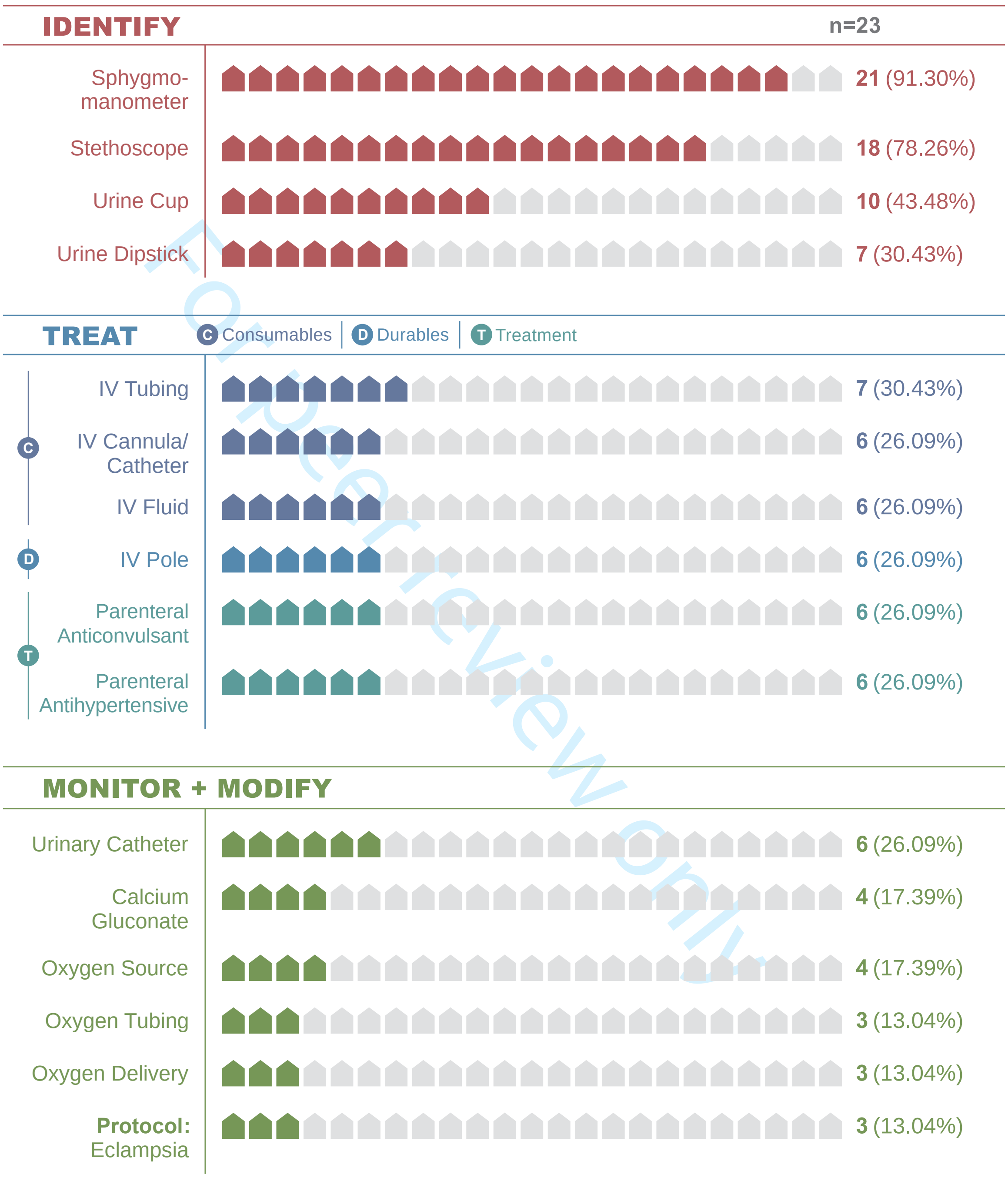

Figure S3. Hypertensive Emergency Clinical Cascade

For peer review only

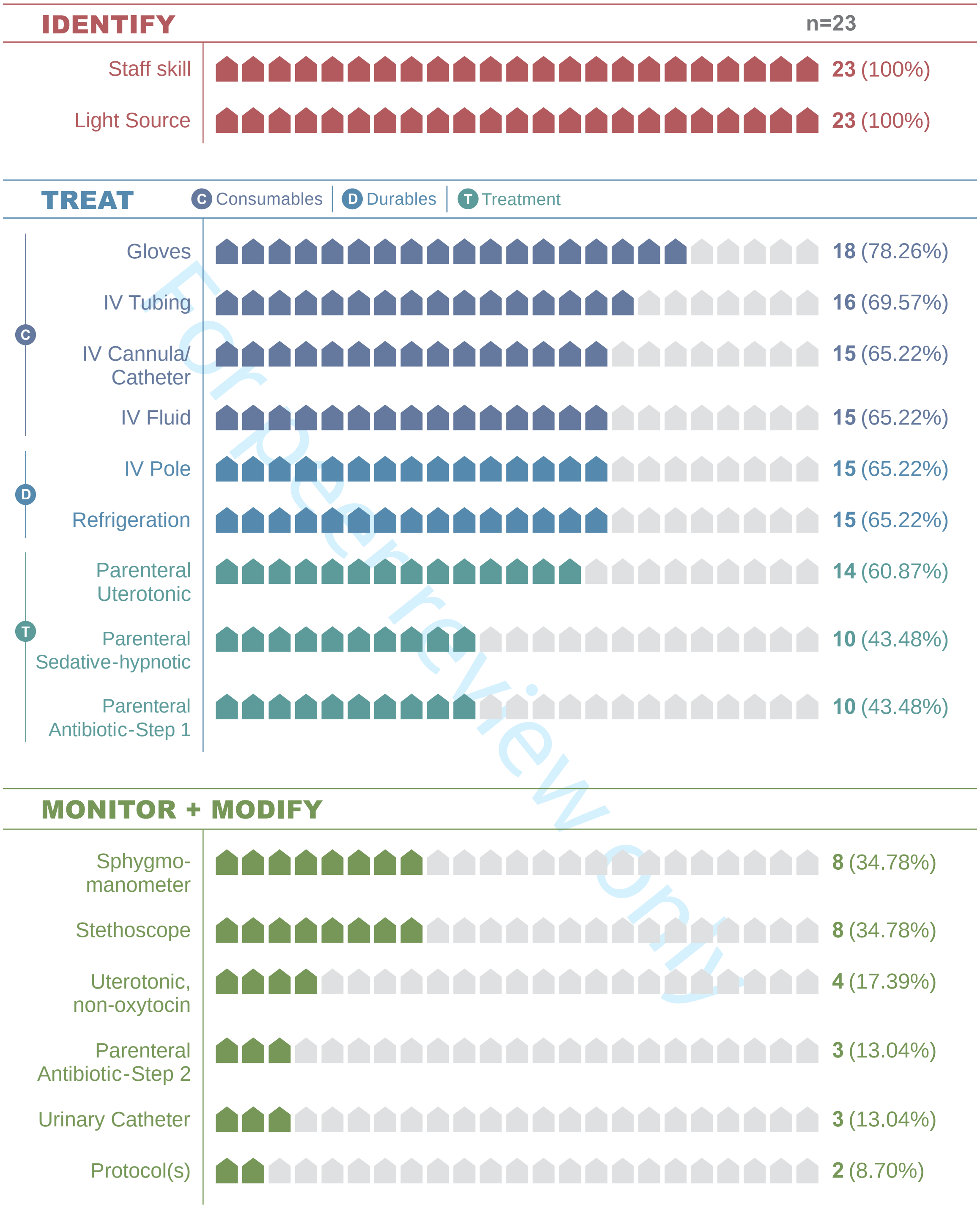

Figure S4. Retained Placenta Clinical Cascade

For peer review only

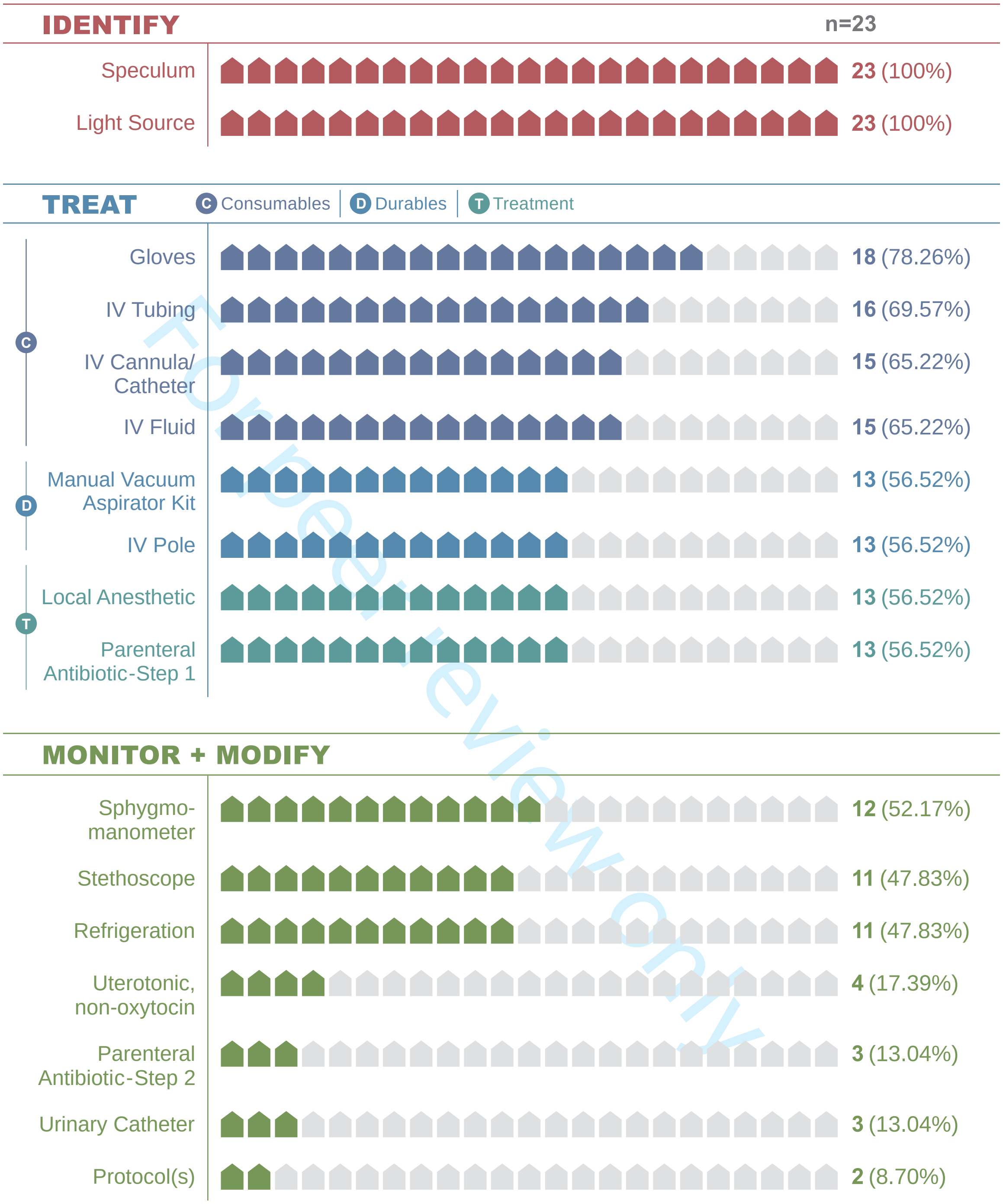

Figure S5. Incomplete Abortion Clinical Cascade

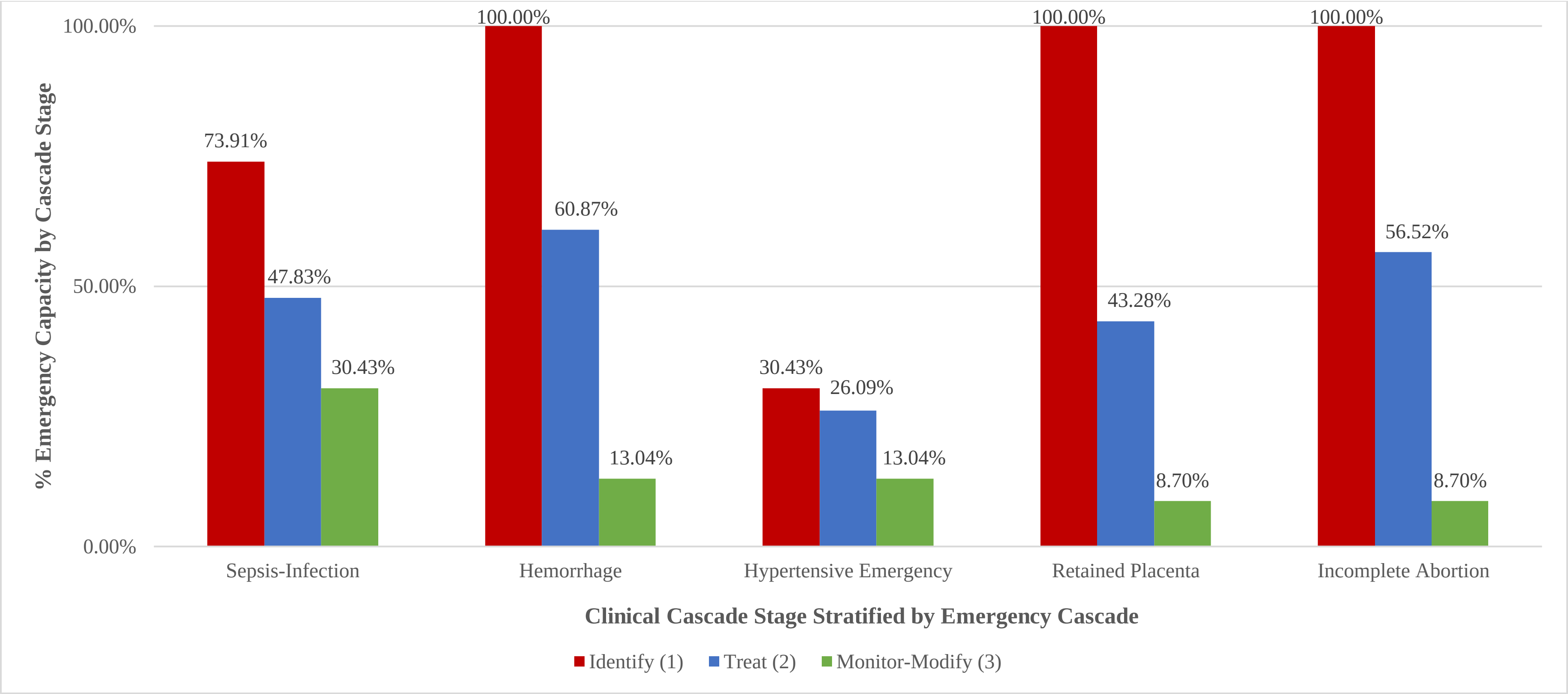

Figure S6. Emergency Readiness Estimates by Emergency Cascade and Stage

For peer review only

STROBE Statement—Checklist of items that should be included in reports of ***cross-sectional studies***

	Item No	Recommendation	Page No
**Title and abstract**	1	(*a*) Indicate the study's design with a commonly used term in the title or the abstract	1
		(*b*) Provide in the abstract an informative and balanced summary of what was done and what was found	2
**Introduction**			
Background/rationale	2	Explain the scientific background and rationale for the investigation being reported	3-4
Objectives	3	State specific objectives, including any prespecified hypotheses	4
**Methods**			
Study design	4	Present key elements of study design early in the paper	4
Setting	5	Describe the setting, locations, and relevant dates, including periods of recruitment, exposure, follow-up, and data collection	4
Participants	6	(*a*) Give the eligibility criteria, and the sources and methods of selection of participants	4
Variables	7	Clearly define all outcomes, exposures, predictors, potential confounders, and effect modifiers. Give diagnostic criteria, if applicable	5
Data sources/ measurement	8*	For each variable of interest, give sources of data and details of methods of assessment (measurement). Describe comparability of assessment methods if there is more than one group	4-5
Bias	9	Describe any efforts to address potential sources of bias	NA
Study size	10	Explain how the study size was arrived at	4
Quantitative variables	11	Explain how quantitative variables were handled in the analyses. If applicable, describe which groupings were chosen and why	5-6
Statistical methods	12	(*a*) Describe all statistical methods, including those used to control for confounding	5-6
		(*b*) Describe any methods used to examine subgroups and interactions	5-6
		(*c*) Explain how missing data were addressed	5
		(*d*) If applicable, describe analytical methods taking account of sampling strategy	NA
		(*e*) Describe any sensitivity analyses	6
**Results**			
Participants	13*	(a) Report numbers of individuals at each stage of study—eg numbers potentially eligible, examined for eligibility, confirmed eligible, included in the study, completing follow-up, and analysed	6
		(b) Give reasons for non-participation at each stage	N/A
		(c) Consider use of a flow diagram	N/A
Descriptive data	14*	(a) Give characteristics of study participants (eg demographic, clinical, social) and information on exposures and potential confounders	6
		(b) Indicate number of participants with missing data for each variable of interest	N/A
Outcome data	15*	Report numbers of outcome events or summary measures	6-14
Main results	16	(*a*) Give unadjusted estimates and, if applicable, confounder-adjusted estimates and their precision (eg, 95% confidence interval). Make clear which confounders were adjusted for and why they were included	6-14

For peer review only

		(*b*) Report category boundaries when continuous variables were categorized	NA
		(*c*) If relevant, consider translating estimates of relative risk into absolute risk for a meaningful time period	NA
Other analyses	17	Report other analyses done—eg analyses of subgroups and interactions, and sensitivity analyses	13
**Discussion**			
Key results	18	Summarise key results with reference to study objectives	15
Limitations	19	Discuss limitations of the study, taking into account sources of potential bias or imprecision. Discuss both direction and magnitude of any potential bias	16
Interpretation	20	Give a cautious overall interpretation of results considering objectives, limitations, multiplicity of analyses, results from similar studies, and other relevant evidence	15-17
Generalisability	21	Discuss the generalisability (external validity) of the study results	15-17
**Other information**			
Funding	22	Give the source of funding and the role of the funders for the present study and, if applicable, for the original study on which the present article is based	17

*Give information separately for exposed and unexposed groups.

Note: An Explanation and Elaboration article discusses each checklist item and gives methodological background and published examples of transparent reporting. The STROBE checklist is best used in conjunction with this article (freely available on the Web sites of PLoS Medicine at http://www.plosmedicine.org/, Annals of Internal Medicine at http://www.annals.org/, and Epidemiology at http://www.epidem.com/). Information on the STROBE Initiative is available at www.strobe-statement.org.

For peer review only

For peer review only - http://bmjopen.bmj.com/site/about/guidelines.xhtml

BMJ Open

Using clinical cascades to measure health facilities' obstetric emergency readiness: testing the cascades model using cross-sectional facility data in East Africa

Journal:	*BMJ Open*
Manuscript ID	bmjopen-2021-057954.R1
Article Type:	Original research
Date Submitted by the Author:	03-Mar-2022
Complete List of Authors:	Whaley, Bridget; Emory University, Behavioral, Social, and Health Education Sciences<br>Butrick, Elizabeth ; University of California San Francisco<br>Sales, Jessica; Emory University, Behavioral, Social, and Health Education Sciences<br>Wanyoro, Anthony; Kenyatta University, Department of OB/GYN<br>Waiswa, Peter; Makerere University, School of Public Health<br>Walker, Dilys; University of California San Francisco Department of Obstetrics Gynecology and Reproductive Sciences, ; University of California San Francisco Global Health Sciences,<br>Cranmer, John; Emory University Woodruff Health Sciences Center,
**Primary Subject Heading**:	Obstetrics and gynaecology
Secondary Subject Heading:	Emergency medicine, Global health, Health services research
Keywords:	OBSTETRICS, Maternal medicine < OBSTETRICS, International health services < HEALTH SERVICES ADMINISTRATION & MANAGEMENT, Quality in health care < HEALTH SERVICES ADMINISTRATION & MANAGEMENT

SCHOLARONE™
Manuscripts

I, the Submitting Author has the right to grant and does grant on behalf of all authors of the Work (as defined in the below author licence), an exclusive licence and/or a non-exclusive licence for contributions from authors who are: i) UK Crown employees; ii) where BMJ has agreed a CC-BY licence shall apply, and/or iii) in accordance with the terms applicable for US Federal Government officers or employees acting as part of their official duties; on a worldwide, perpetual, irrevocable, royalty-free basis to BMJ Publishing Group Ltd ("BMJ") its licensees and where the relevant Journal is co-owned by BMJ to the co-owners of the Journal, to publish the Work in this journal and any other BMJ products and to exploit all rights, as set out in our licence.

The Submitting Author accepts and understands that any supply made under these terms is made by BMJ to the Submitting Author unless you are acting as an employee on behalf of your employer or a postgraduate student of an affiliated institution which is paying any applicable article publishing charge ("APC") for Open Access articles. Where the Submitting Author wishes to make the Work available on an Open Access basis (and intends to pay the relevant APC), the terms of reuse of such Open Access shall be governed by a Creative Commons licence – details of these licences and which Creative Commons licence will apply to this Work are set out in our licence referred to above.

Other than as permitted in any relevant BMJ Author's Self Archiving Policies, I confirm this Work has not been accepted for publication elsewhere, is not being considered for publication elsewhere and does not duplicate material already published. I confirm all authors consent to publication of this Work and authorise the granting of this licence.

For peer review only

Using clinical cascades to measure health facilities' obstetric emergency readiness: testing the cascades model using cross-sectional facility data in East Africa

Corresponding Author:
Bridget Whaley
1520 Clifton Road NE
Atlanta, GA, 30322
whaley.bridget@gmail.com

Author Information:
Bridget Whaley[1], Elizabeth Butrick[2], Jessica M. Sales[1], Anthony Wanyoro[3], Peter Waiswa[4], Dilys Walker[2], John N. Cranmer[5]

1 Rollins School of Public Health, Emory University, Atlanta, Georgia, United States of America
2 Department of Obstetrics, Gynecology, and Reproductive Sciences, University of California San Francisco, San Francisco, California, United States of America
3 Department of Obstetrics and Gynecology, Kenyatta National Hospital, University of Nairobi, Nairobi, Kenya
4 School of Public Health, Makerere University, Kampala, Uganda
5 Emory University, Atlanta, Georgia, United States of America

Abstract Word Count: 274

Manuscript Word Count: 4,948

Number of Tables and Figures (manuscript): 5

Number of Tables and Figures (Online Supplementary Document): 16

For peer review only

Abstract

Objectives: Globally, hundreds of women die daily from preventable pregnancy-related causes, with the greatest burden in Sub-Saharan Africa. Five key emergencies– bleeding, infections, high blood pressure, delivery complications, and unsafe abortions– account for nearly 75% of these deaths. Skilled clinicians with strategic supplies could prevent most deaths. In this study, we (1) measured facility readiness to manage common obstetric emergencies using the clinical cascades and signal function tracers; (2) compared these readiness estimates by facility characteristics; and (3) measured cascading drop-offs in resources.

Design: A facility-based cross-sectional analysis of resources for common obstetric emergencies.

Setting: Data were collected in 2016 from 23 hospitals (10 designated comprehensive emergency obstetric care (CEmOC) facilities) in Migori County, western Kenya, and Busoga Region, eastern Uganda, in the Preterm Birth Initiative (PTBi) study in East Africa. Baseline data were used to estimate a facility's readiness to manage common obstetric emergencies using signal function tracers and the clinical cascades model. We compared emergency readiness using the proportion of facilities with tracers (signal functions) and the proportion with resources for identifying and treating the emergency (cascade Stages 1-2).

Results: The signal functions overestimated practical emergency readiness by 23 percentage points across five emergencies. 42% of CEmOC-designated facilities could perform basic emergency obstetric care (BEmOC). Across the three stages of care (*identify*, *treat*, and *monitor-modify*) for five emergencies, there was a 28% pooled mean drop-off in readiness. Across emergencies, the largest drop-off occurred in the treatment stage. Patterns of drop-off remained largely consistent across facility characteristics.

Conclusions: Accurate measurement of obstetric emergency readiness is a prerequisite for strengthening facilities' capacity to manage common emergencies. The cascades offer stepwise, emergency-specific readiness estimates designed to guide targeted maternal survival policies and programs.

Keywords: Obstetrics, maternal medicine, international health services, quality in health care

Strengths and limitations of this study

- Measuring emergency obstetric readiness according to the clinical cascades provides a more nuanced picture of where readiness is lost for emergency care; cascade estimates support targeted strategies for closing gaps in critical emergency obstetric supplies.
- To the authors' knowledge, this is the first study to compare signal functions and clinical cascade estimates of obstetric emergency readiness across countries.
- This study expands previous scholarship by measuring emergency readiness by level of care (C-section capability), facility ownership (government vs. private hospital), and country (Kenya vs. Uganda).
- We did not have tracer item data to estimate readiness for assisted vaginal deliveries (AVD) - one of six signal functions and clinical cascades.
- Resource availability alone does not comprehensively describe emergency readiness and a clinician's ability to use the resources and perform targeted interventions is a critical aspect; however, skill and knowledge are not easily measured and not included in either the signal function tracers or clinical cascade methodology.

For peer review only

INTRODUCTION

In 2017, more than 800 women died each day from preventable pregnancy- and childbirth-related causes, resulting in nearly 300,000 annual deaths[1,2]. A few major obstetric emergencies account for nearly 75% of these deaths: severe bleeding (hemorrhage), infections (sepsis), high blood pressure (pre-eclampsia and eclampsia), delivery complications (prolonged or obstructed labor), and unsafe abortions[3]. Common obstetric emergency deaths can be effectively prevented when skilled healthcare providers have the supplies necessary to identify and manage emergencies and can transfer patients to higher levels of care when an emergency exceeds the facility's scope of care[3]. Labor-related complications require immediate and easy access to high-quality emergency obstetric care (EmOC). Basic Emergency Obstetric Care (BEmOC) is the first level of emergency management for the most common causes of death. BEmOC requires essential supplies, durable equipment, and emergency-specific drugs. When obstetric emergencies are beyond the capacity of BEmOC facilities, patients can be referred to Comprehensive Emergency Obstetric Care (CEmOC) facilities. CEmOC includes all BEmOC functions and adds resources for blood transfusion and surgery (such as cesarean-section, C-section)[3]. Mobilizing and dispensing the clinical resources required to manage basic obstetric emergencies may be a critical step for reducing persistently elevated Maternal Mortality Ratios (MMRs) in Sub-Saharan African contexts such as Kenya and Uganda. Therefore, accurate measurement of a facility's readiness to manage the common emergencies that drive maternal deaths is urgently needed.

The World Health Organization (WHO) first identified the resources necessary to manage common obstetric emergencies in the 1990s[4]. This recommended approach evolved into the "signal functions," consisting of six clinical actions (three medical treatments and three manual procedures) for managing common obstetric emergencies. BEmOC includes three medical treatments and three manual procedures. The three medical treatments include administering parenteral (1) antibiotics, (2) uterotonics, and (3) anticonvulsants/antihypertensives. The three manual procedures include (4) manually removing retained placentas, (5) removing retained products of conception, and (6) performing assisted vaginal deliveries[5]. CEmOC extends BEmOC care by adding two additional actions: performing (7) obstetric surgeries (such as C-section) and (8) blood transfusions[5]. Thus, all designated CEmOC facilities should be prepared to perform all BEmOC signal functions, in addition to offering surgery and blood transfusion. In the signal function model, specific items (i.e., "tracers") that are considered most critical for performing the clinical actions are used as proxies to measure a facility's capacity to manage obstetric emergencies[5-11].

Signal function estimates of emergency readiness have been the dominant method for assessing obstetric readiness at facilities worldwide[12-15]. BEmOC signal functions indicators have been used to estimate a facility's overall BEmOC emergency readiness using the percent of tracer items present at the facility[15]. WHO's Service Readiness Index (SRI) defines a facility's obstetric emergency readiness using the six clinical actions that define BEmOC[5]. In the SRI methodology, the average number of tracer items present on the day of the facility assessment determines a facility's overall obstetric emergency readiness[14,15]. This method utilizes the signal functions framework. Traditionally, signal function readiness estimates are reported as a single indicator, the proportion of facilities with the tracer items for all medical treatments and manual procedures.

In recent years, scientists and practitioners have increasingly called for revised approaches to address weaknesses in the signal functions[7,9,16-21]. One particular weakness is the signal function's inability to predict readiness for *specific* emergencies[16,18]. Further, this approach does not identify strategic resources for managing multiple emergencies nor suggest indicators for system-wide emergency readiness[21]. Also, the signal functions do not distinguish between the resources required to first *identify* the emergency and then the consumable supplies and durable goods required to deliver targeted treatment/intervention for the emergency[21]. Thus, to further reduce delivery-related mortality, a more

For peer review only - http://bmjopen.bmj.com/site/about/guidelines.xhtml

robust set of measurable indicators that are emergency-specific and relevant for multiple levels of the health system are needed[21]. The clinical cascade model is designed to fill these gaps.

The cascade model was designed to measure BEmOC-readiness at global emergency obstetric facilities. It is informed by three models–the original signal functions, the Systemic Capacity Hierarchy of Needs, and the Clinical Care Continuum (Spectrum of Engagement)[22,23]. The cascades were designed as a clinically-oriented, population-relevant family of indicators. The core indicators measure facility readiness to manage the six common obstetric emergencies by reporting the percentage of facilities with emergency resources to identify, treat and monitor-modify therapy[21-23]. Operationally, the cascades define emergency readiness as the proportion of facilities that have the resources (including the drugs, supplies, and equipment) to *identify* an emergency and *treat* it. A facility's ability to *monitor and modify* the initial treatment as clinically indicated based on patient response was not a foundational measure of readiness for the presenting emergency. Rather, it has been proposed as an indicator of care quality[21].

Unlike the wealth of research on signal functions, the clinical cascades have been tested in fewer geographic locations. There are limited studies comparing this emerging metric with the signal functions as methods to assess facilities' readiness to handle basic obstetric emergencies. To date, one published study compared estimates of obstetric readiness based on signal functions and clinical cascades. Two additional studies were conducted in Guatemala and Ethiopia and are pending publication. The published study from BEmOC-designated facilities in Kakamega County, Kenya found a 55% overestimate of practical emergency management readiness using the signal function tracers compared to the clinical cascades[21]. Moreover, a 33% pooled mean drop-off in readiness across three stages for all cascades (emergencies) was identified[21]. A similar pattern of emergency readiness loss also occurred for newborn emergencies in a published study from Kenya and Uganda. In those countries, a pooled mean 30% drop-off in readiness was measured across the three stages of care for all newborn emergencies[24]. This study, however, did not compare clinical cascade-based estimates of readiness against signal function estimates since no standard signal function indicators for newborns exist[9]. The findings from these two published clinical cascade studies warrant additional investigation to determine the relevance and transferability of the obstetric clinical cascade model for other global contexts, health systems, and levels of facility-based care. A more comprehensive analysis of a facility's readiness to handle obstetric emergencies, such as the cascade analysis, may be critical to guide emergency-specific or supply-specific interventions to close gaps in the availability of emergency supplies that are critical to maternal survival.

Aims

Building off the wealth of research on the signal functions and formative research on clinical cascades, this cross-sectional facility-based study was designed to assess readiness for pregnancy-related emergencies at designated BEmOC and CEmOC facilities in Migori County, Kenya and the Busoga region of Uganda. The facility data used in this analysis were originally collected as part of a pair-matched, cluster randomized controlled trial evaluating a package of facility-based interventions to improve care for preterm infants[25]. This nested analysis was designed to: (1) estimate facility readiness to manage common obstetric emergencies using clinical cascades and signal functions; (2) compare these estimates based on differences in facility characteristics (e.g., designated level of care, facility ownership, and country); and (3) test if the cascading loss of emergency obstetric resources first identified in Kenya was also present in other global settings. The existing studies on the cascades model suggest that using signal functions to assess maternal emergency readiness may convey a false sense of security by indicating a higher level of emergency preparedness than practically exists in facilities[21,24].

METHODS

Study design and data collection

We conducted a cross-sectional analysis of facility data collected in 2016-2019 by the Preterm Birth Initiative Kenya and Uganda Implementation Research Collaborative (PTBi)[25]. Data from pre-

For peer review only

intervention baseline facility assessments at 23 facilities in the PTBi study were used to create the signal function and clinical cascade estimates of emergency obstetric readiness[25]. Data from all 17 PTBi facilities in Migori County (western Kenya) and 6 in Busoga Region (eastern Uganda) were used. We used the baseline data (2016) to capture facility readiness prior to any PTBi interventions.

Research assistants used standardized forms to visually identify emergency resources during the on-site physical inventory of resources. They captured data about facility characteristics, obstetric drugs, consumable supplies, durable goods, and the presence of emergency guidelines and protocols. Researchers recorded both the presence/absence of the item and its location (i.e., Unit). In this analysis, we used a resource's presence or absence at the facility level to estimate facility-level readiness regardless of the unit in which the items were located.

Emergency readiness

Signal functions

Traditionally, signal function readiness estimates are reported as a single indicator, the proportion of facilities with the tracer items for all medical treatments and manual procedures[21]. However, for the purposes of our analysis, we reported additional indicators from the clinical cascades: (1) total readiness estimates across all SRI/signal functions; (2) readiness estimates by type of signal function (medical treatment vs. manual procedure); and (3) individual readiness estimates for each signal function. This approach allowed for a direct comparison to clinical cascade estimates.

Clinical cascades

Given that the signal functions do not have quality indicators for monitoring and modifying the primary treatment based on a patient's clinical response (Stage 3 of the clinical cascades), we defined mean clinical cascade readiness using Stages 1 and 2 of the clinical cascades (*identify* and *treat*). Although readiness at Stage 3 of the clinical cascade of care is not reported in comparative analyses, it is included in the analyses that examine readiness along the three stages of clinical care (*identify*, *treat*, *monitor-modify*). The cascading drop-off in readiness across these three stages is an independent indicator that is not used to directly compare signal function and clinical cascade estimates. Rather, it has been proposed as a proxy indicator for a facility's readiness to monitor the initial therapy and modify that therapy based on patient response. This proposed quality of care indicator only uses commodities (e.g., protocols, medications, supplies) and does not measure clinician skill. We measured clinical cascade estimates of readiness at the individual resource level (calculating percentages of facilities with each individual resource). This approach allowed us to demonstrate precisely where in the clinical cascade readiness drops off. We estimated overall emergency readiness across sites as the percentage of facilities with all resources necessary to complete the *identify* and *treat* stages of care. Of note, readiness at each of the three stages of clinical care (*identify* the emergency, *treat* it, and *monitor/modify* therapy) is cumulative. For example, readiness at Stage 2 is calculated from the percentage of facilities with all resources required to first identify the emergency (Stage 1).

Operational definitions

In the absence of certain variables, we used proxies for the missing tracer items to not unduly penalize a facility's readiness estimate using the SRI/signal function tracers - this was especially true for consumable supplies and durable goods, such as refrigeration (electrical power as proxy for refrigeration) and light source (electrical power or operational flashlight as proxy for a light source). Moreover, some tracer items, namely drugs, are not concretely defined in the SRI/signal functions model. When drugs were not explicitly defined by the SRI/signal functions, we referred to WHO's first-line recommendations for obstetric care to create operational definitions[5,26,27]. For instance, in the SRI/signal functions, antibiotics are broadly defined as "parenteral antibiotics." To transform this broad drug category into an operational indicator, we used WHO's 3-step sequence of obstetric antibiotic therapy escalation to define

For peer review only - http://bmjopen.bmj.com/site/about/guidelines.xhtml

readiness (Ampicillin, Gentamicin, and Metronidazole)[5,26,27]. In other instances, data on both tracers and potential proxies were missing. This was true for most items from the assisted vaginal delivery cascade (AVD). Consequently, we were unable to measure AVD readiness using the SRI/signal functions or the clinical cascades. **Table S1** in the **Online Supplementary Document** provides a list of the emergency resources used to define the SRI/signal functions and clinical cascades.

Analysis

We first described emergency resource availability for individual resources using percentages. In describing the availability of emergency resources, we reported percentages and frequencies for categorical variables. For continuous variables, we reported the medians with interquartile ranges (IQR) because they were not normally distributed (e.g., delivery volume).

Aggregate readiness across multiple emergencies or multiple stages of care was reported as the overall pooled mean. Drop-offs in readiness (i.e., the percentage of facilities prepared to handle the five emergencies) between each stage of care and across each emergency cascade were reported as percentages with the standard deviation. We reported estimates of overall emergency readiness using the mean for several reasons: 1) the SRI/signal function method reports means, and we wanted this study's findings to be benchmarked against globally reported indicators; (2) means are most frequently used in the published literature; and (3) with few observations, the median is unlikely to effectively represent central tendency.

For the purposes of comparing the clinical cascades and SRI/signal functions, we reported: (1) mean overall (pooled) readiness across all five emergencies for the signal functions and clinical cascades; (2) mean overall (pooled) readiness by type of signal function (medical treatment versus manual procedure); (3) mean overall (pooled) readiness for each emergency (i.e., SRI/signal function and clinical cascade); and (4) mean overestimated readiness (pooled mean for SRI/signal function readiness minus clinical cascade readiness). Mean overall (pooled) readiness was calculated using the mean individual readiness estimate for each emergency (i.e., SRI/signal function and clinical cascade). First, we reported emergency readiness estimates for all facilities. Next, we stratified emergency readiness by facility characteristics (level of care [reported C-section capability], ownership [public/government versus private], and country [Kenya versus Uganda]). Reported C-section service delivery at a facility was used as a proxy for identifying designated CEmOC facilities (since designated BEmOC facilities do not have this capability).

For the purpose of calculating the drop-off in readiness according to the clinical cascade model, we reported the decrease in the number of percentage points in facilities ready to handle the five emergencies by stage of care of the clinical cascade, by emergency, and overall across the three stages of care for five emergencies (pooled mean). To determine readiness drop-off by stage of care, we subtracted readiness (i.e., percentage of facilities ready to manage the five emergencies) at the end of a stage from readiness at the end of the preceding stage. For example, to calculate readiness drop off during Stage 1 (*identify*) for each emergency, we subtracted readiness at the end of Stage 1 from 100% and then calculated the mean and SD across all five emergencies by stage. This provided the pooled mean readiness drop-off across all three stages of care (*identify*, *treat*, *monitor-modify*). From these estimates, we calculated drop-off in emergency readiness by clinical cascade by calculating the mean across the three stages of care for each clinical cascade (i.e., emergency) and producing five estimates of mean drop-off in readiness. The pooled mean across the five cascades determined the overall drop-off in readiness across emergencies and stages of care. Next, estimates were stratified by (1) level of care; (2) facility ownership; and (3) country.

Ethics

The Kenya Medical Research Institute (KEMRI/SERU/CCR/0034/3251), Makerere University School of Public Health (MUSPH HDREC 395), and the UCSF (16-19162) Institutional Review Boards (IRBs)

For peer review only

approved the trial prior to primary data collection. Emory University's IRB determined the nested analysis of these de-identified data was not human subjects research (STUDY 00001202).

Patient and public involvement

The parent study results were shared with health providers at the facilities. The research question and outcome measures for this nested study of previously collected PTBi-data were shaped by global maternal survival research priorities and reported experiences of women of reproductive age. For this nested study, the study findings will be shared directly with the participating PTBi facilities. Patients and the public were not directly involved in the design or conduct of the research.

RESULTS

Facility characteristics

In **Tables S2-S3,** we present facility characteristics. Of the 23 facilities, 82.6% were government-owned and 17.4% were privately-owned hospitals. Thirteen of the 23 facilities did not have reported C-section capabilities (i.e., were designated BEmOC facilities). All Ugandan facilities reported C-section capability and are, therefore, designed to offer CEmOC-facilities. By contrast, 76.5% of the Kenyan facilities were designated BEmOC hospitals.

Signal function estimates of emergency readiness

Overall readiness

The overall mean estimate of readiness as defined by the availability of tracer items for the five SRI/signal functions was 69.6% (**Table 1)**. There was little variability in readiness across the five signal functions (ranging from 60.9% for uterotonics) to 78.3% for anticonvulsants). The 69.9% SRI/signal function estimates of readiness did not differ for emergencies requiring manual procedures compared to those requiring medication therapy.

Readiness by facility characteristics

As expected, SRI/signal function readiness estimates differed by level of care (**Table S4**), facility ownership (**Table S5**), and country (**Table S6**). Overall, BEmOC readiness estimates were higher among facilities with C-section capability (designated CEmOC facilities), those that were privately-owned, and those in Uganda.

Clinical cascade readiness

Readiness Against the SRI/Signal Functions

Across all five emergencies, the signal function tracers overestimated obstetric emergency readiness by 22.6 percentage points (**Table 1, Figure S1**). The pooled mean signal function readiness estimate was 69.6%, while readiness as measured by Stage 2 of the cascade model was substantially lower at 47.0%. Notably, there was wide variability in the signal function overestimate, ranging from 0.0 (manage hemorrhage/uterotonics) to 52.2 percentage points (hypertensive emergencies/ anticonvulsant, **Table 1**). The 23-percentage point overestimation seen in the full sample remained largely consistent after stratifying by facility characteristics (**Table 2)**.

Readiness by emergency

When examining clinical cascade estimates of emergency readiness by emergency at the individual resource level, we saw an overall pattern of readiness drop-off across the stages of care (from Stage 1 to Stage 3, **Table S7**, **Figures S2-S6**). The amount of readiness drop-off at each stage and the specific resources driving the drop-off differed by emergency.

For peer review only - http://bmjopen.bmj.com/site/about/guidelines.xhtml

Table 1. Emergency readiness estimates comparing clinical cascades and signal functions, all facilities [(1)]

**Clinical Cascade**	**Signal Functions**	**Clinical Cascades**	**Overestimated Readiness**
(Signal Function)	**% Readiness, Tracer Items**	**% Readiness, Stage 2**	**Percentage Point Difference [Signal Functions (-) Cascade]**
**Medical Treatments**			
**Manage Sepsis - Infection** *(Antibiotic)*	69.6%	47.8%	21.7
**Manage Hemorrhage** *(Uterotonics)*	60.9%	60.9%	0.0
**Manage Hypertensive Emergency** *(Anticonvulsant)*	78.3%	26.1%	52.2
Medical Readiness, Overall Mean (pooled)	69.6%	44.9%	24.6
**Manual Procedures**			
**Manage Retained Placenta** *(Removal of retained placenta)*	69.6%	43.5%	26.1
**Manage Incomplete Abortion** *(Removal of retained products of conception)*	69.6%	56.5%	13.1
Manual Readiness, Overall Mean (pooled)	69.6%	50.0%	19.6
**Overall Mean Readiness (pooled)**	**69.6%** *Signal Function Estimate*	**47.0%** *Cascade Estimate*	**22.6** *Percentage Point Overestimation by Signal Functions*

[(1)] n = 23 facilities

For peer review only

Table 2. Overestimation of emergency readiness by signal function tracers compared to clinical cascades, by facility characteristics

**Clinical Cascade** *(Signal Function)*	**Percentage Point Difference in Readiness [Signal Functions (-) Cascade]**						
		Reported C-Section Capability		*Ownership*		*Country*	
	**All**	**C-section**	**No C-section**	**Government**	**Private**	**Kenya**	**Uganda**
**n**	23	10	13	19	4	17	6
**Manage Sepsis - Infection** *(Antibiotic)*	21.7	40.0	7.7	21.1	25.0	11.8	50.0
**Manage Hemorrhage** *(Uterotonics)*	0.0	0.0	0.0	0.0	0.0	0.0	0.0
**Manage Hypertensive Emergency** *(Anticonvulsant)*	52.2	60.0	46.2	47.4	75.0	52.9	50.0
**Manage Retained Placenta** *(Removal of retained placenta)*	26.1	0.0	46.2	31.6	0.0	35.3	0.0
**Manage Incomplete Abortion** *(Removal of retained products of conception)*	13.1	10.0	15.4	10.5	0.0	11.8	16.7
**Mean Percentage Point Overestimation by Signal Functions (pooled)**	**22.6**	**22.0**	**23.1**	**22.1**	**20.0**	**22.4**	**23.3**

For peer review only

Readiness by cascade

There were differences in readiness drop-off along the stages of care from identification of the obstetric emergency (Stage 1) through monitoring or modifying therapy (Stage 3, **Table 3**). These differences varied least for the sepsis cascade (SD = 5.0) and most for the hypertension cascade (SD = 35.4). There was also substantial variability in *when* the drop-off in readiness occurred. For hypertensive emergencies, the largest drop-off occurred in the identification stage (69.6%). In contrast, there was no readiness drop-off in the identification stage for the hemorrhage, retained placenta, or incomplete abortion cascades. Of note, Stage 1 for the hemorrhage and retained placenta cascades is based on staff skill alone. For this commodity-based study, we assumed staff skill to be 100% for all emergencies, but it was not measured (skill is explicitly shown for the hemorrhage cascade since it does not require any commodities for identification)[21].

When examining drop- off in readiness by facility characteristics, there were subtle differences by level of care (**Table S8**), ownership (**Table S9**), and country (**Table S10**). For instance, at facilities *with* reported C-section capability, the percentage of drop-off across the stages of care varied least for sepsis (SD = 17.3) and most for hemorrhage (SD = 30.0, **Table S8**). Among facilities *without* C-section capability, the drop-off pattern was the same as seen in the full sample - it varied least for sepsis (SD = 11.8) and most for hypertensive emergencies (SD = 42.4).

For peer review only

Table 3. Mean drop-off in readiness by cascade and stage of care among all facilities[(1)]

Readiness Drop-Off by Stage of Care				Readiness Drop-Off by Emergency	
**Clinical Cascade**	**1**	**2**	**3**	***Mean Drop-Off Across 3 Cascade Stages of Care***	***SD***
	**Identify**	**Treat**	**Monitor-Modify**		
	--	--	--	**28.4%** [(2)]	**24.2** [(3)]
**Sepsis-Infection**	26.1%	26.1%	17.4%	23.2%	5.0
**Hemorrhage**	0.0%	39.1%	47.8%	29.0%	25.5
**Hypertensive Emergency**	69.6%	4.4%	13.0%	29.0%	35.4
**Retained Placenta**	0.0%	56.5%	34.8%	30.4%	28.5
**Incomplete Abortion**	0.0%	43.5%	47.8%	30.4%	26.5
**Overall Drop-Off by Stage of Care**					
Drop-Off By Stage of Care (Across All Emergencies), Pooled Mean	**19.1%**	**33.9%**	**32.2%**		
SD	**30.4**	**19.8**	**16.4**	**3.0** [(4)]	

[(1)] n = 23 facilities
[(2)] Pooled mean readiness drop-off across 3 clinical cascade stages of care and 5 emergencies
[(3)] Mean of the standard deviations
[(4)] Standard deviation across 3 stages of care and 5 emergencies

For peer review only

Readiness by stage of care

This study revealed a pattern of 28.4% drop-off in readiness across emergencies and stages of care (SD = 3.0) despite moderate variability in where the drop-off occurred across these stages (average SD across stages = 24.2, **Table 3**). Overall, the largest drop off took place in *treating* the obstetric emergency (Stage 2, 33.9%), and the smallest drop-off took place in identifying the emergency (Stage 1, 19.1%). **Figure 1** depicts the pattern of readiness drop-off across the stages of care and emergencies.

The overall readiness drop-off based on facility characteristics (reported C-section capacity, ownership and country) was similar to that observed in the full facility sample (**Table 4**). For instance, we found similar drop-off and variability based on reported C-section capacity. There was a 26.7% drop-off in readiness across emergencies and stages of care among facilities with C-sections (SD = 4.18) and 29.7% among those without (SD = 3.4). Consistent with the unstratified results, the largest drop-off in readiness among facilities without reported C-section capability occurred in the treatment stage (43.1%) (**Table S8**). However, among facilities with reported C-section capability, the largest drop-off occurred in the final stage of care—monitoring-modifying therapy (38.0%). Similar patterns emerged across facility ownership (**Table 4**, **Table S9**) and country (**Table 4**, **Table S10**).

For peer review only

Table 4. Mean drop-off in readiness by facility characteristics

Clinical Cascade	Mean Drop-Off Across 3 Cascade Stages of Care						
		Reported C-section Capacity		Ownership		Country	
	All	C-section	No C-section	Government	Private	Kenya	Uganda
n	23	10	13	19	4	17	6
Sepsis-Infection	23.2%	20.0%	25.6%	26.3%	8.3%	21.6%	27.8%
Hemorrhage	29.0%	30.0%	28.2%	28.1%	33.3%	27.5%	33.3%
Hypertensive Emergency	29.0%	30.0%	28.2%	28.1%	33.3%	27.5%	33.3%
Retained Placenta	30.4%	26.7%	33.3%	31.6%	25.0%	31.4%	27.8%
Incomplete Abortion	30.4%	26.7%	33.3%	31.6%	25.0%	31.4%	27.8%
**Overall Drop-Off**							
Mean[1]	**28.4%**	**26.7%**	**29.7%**	**29.1%**	**25.0%**	**27.8%**	**30.0%**
SD[2]	**3.0**	**4.1**	**3.4**	**2.4**	**10.2**	**4.0**	**3.0**

(1) Pooled mean readiness drop-off across 3 stages of care and 5 emergencies
(2) Standard deviation of mean drop-off across 3 stages of care and 5 emergencies

For peer review only

DISCUSSION

This study expands the global scholarship on basic obstetric emergency readiness by confirming previously published patterns of 1) resource loss across three stages of emergency care (*identify*, *treat*, *monitor*-modify) and 2) signal function tracer overestimation of emergency readiness. It expands previous scholarship by highlighting how emergency readiness differs by level of care, facility ownership, and country. Results from this study aligned with those found in the published comparison of SRI/signal functions and clinical cascades. The signal functions consistently overestimate obstetric emergency readiness. We found that signal functions overestimated readiness by 22.6 percentage points. This is about half of what was reported in the formative study (54.5 percentage points)[21]. However, the formative study only included designated BEmOC facilities, whereas nearly half (43.5%) of the facilities in this study were designated CEmOC facilities. Despite some operational (e.g., variable inclusion/definitions) and contextual (e.g., country/region) differences between studies, any overestimation of practical emergency readiness may be profoundly clinically relevant—particularly since elevated maternal mortality persists globally despite increased rates of facility-based deliveries. The 22.6 percentage point overestimation of readiness by the signal functions in this study—particularly since it includes designated CEmOC facilities—is profoundly concerning for advancing global maternal survival.

As in previous studies, we found a consistent drop-off in readiness across the stages of care. Across three stages of care for five emergencies, we found a mean 28.4% drop-off in readiness which was profoundly consistent ((SD = 3.0, **Table 3**). The published study of 44 BEmOC facilities in Kenya found a very similar drop-off pattern of 33.0% which also varied little (SD=0.4)[21]. Moreover, in a study of the clinical cascades as applied to neonatal emergencies, the mean drop off in readiness was 30.0% across emergencies and stages of care (based on 2016 data)[24]. Given that the 30% aggregate drop off for neonatal emergencies comes from the same facilities used in this maternal study, the mean readiness drop-off indicator may be an indicator of system-level emergency readiness, as suggested previously[21]. Together, these three studies suggest a drop-off in readiness of approximately 30% across emergencies and stages of care in both maternal and neonatal contexts across country contexts and levels of care. Together with the stages of care and condition-specific indicators, the aggregate drop off may be utilized to guide resource allocation and interventions and as a means of monitoring and comparing readiness across facilities, health systems, countries, or geographic regions. There is growing evidence that global multinational agencies, program planners, and policy makers should consider replacing the current SRI methods and the signal function estimates of readiness with the clinical cascades.

This is the first known study to measure how country, facility ownership, and level of care impact clinical cascade readiness. Overall estimates of readiness (according to both signal functions and clinical cascades) were higher among Ugandan than Kenyan facilities (73.3% vs. 68.2% according to the signal functions and 50.0% vs. 45.9% according to the clinical cascades). These results were expected given the level of care offered at the health facilities included in each country's sample. Ugandan facilities were larger district or regional hospitals, while the Kenyan facilities were primarily smaller, subcounty hospitals and health centers. Likewise, estimates of readiness (based on both signal functions and cascades) were higher among private facilities than government facilities, with nearly all private facilities prepared to handle basic obstetric emergencies according to the signal clinical cascades (75.0%). Differences in readiness by level of care (among CEmOC versus BEmOC-designated facilities) were evident as well (and, as previously mentioned, likely driving differences by facility ownership and country). By definition, all facilities with C-section capability are designated CEmOC facilities that should be prepared to manage all BEmOC emergencies in addition to performing C-sections and blood transfusions. However, this study demonstrated that even designated CEmOC facilities were not ready to perform all BEmOC functions. This gap was present when emergency readiness was estimated using both the signal functions (80.0%) and clinical cascades (58.0%). This gap suggests that facilities designated to offer CEmOC were not practically equipped to handle the complexity of obstetric emergencies required by their designation. If seen across a larger sample, the results could suggest the need for additional

For peer review only

investment to equip designated CEmOC facilities with the supplies needed to handle the full range of obstetric emergencies.

This study has six primary limitations. First, there was a slight variation in the resources used to construct the signal functions and clinical cascades between this study and the two published studies because this paper used updated WHO definitions that were not used in the first two studies. Further, there are minor between-country variations in how some commodities are defined. Second, we used data from a single point in time. Given the unpredictable availability of consumable resources and drugs in some global health systems, estimating emergency readiness using a single time point may be insufficient to capture a facility or system's long-term readiness[18,28]. Collecting data from the same set of facilities at more frequent intervals could provide more accurate readiness data and reveal patterns in resource availability based on supply procurement. Third, due to the absence of select variables, we used proxies for missing tracer items. These proxies may not capture the nuances of actual resource availability. For instance, facilities without electricity often use kerosene-powered refrigerators. With information on neither refrigeration nor kerosene power availability, we used electricity alone as a proxy for refrigeration needed to store select drugs. Consequently, facilities without electricity and using kerosene-powered refrigerators would be unintentionally reported as lacking refrigeration in these analyses. Fourth, more broadly, measuring the availability of resources alone does not take into account the quality of the resource, the number of back-up resources available for sustained care, nor clinicians' ability to use the resources effectively for emergency care[24,29]. Both the signal functions/SRI and clinical cascade estimates of readiness rely on emergency supply availability. Consequently, in commodity-based metrics, it is not possible to estimate actual clinician skill for diagnosing and treating disorders. By extension, although the clinical cascade stages of care are based on clinical action (e.g., identify/diagnose, treat, monitor-modify), it is difficult to reliably and affordably measure these clinical actions at scale in national or regional health systems. When possible to measure, information on resource functionality (e.g., drug expiration dates), facility staffing levels, clinician knowledge and skill, and actual reported performance of the six signal function clinical actions would allow for more precise estimates of practical emergency readiness at the facility level. Fifth, although resources were reported as present in the facilities, we could not determine how accessible resources were during emergencies. Given the importance of timing in an emergency, resource location and availability are critical and should be explored in future research. Sixth, future research could link cascade estimates of readiness to facilities' adverse clinical outcomes (e.g., maternal mortality ratio, maternal near miss, severe maternal outcomes, or prevalence of specific emergencies) to determine the empiric relationship between clinical cascade estimates of readiness and obstetric clinical outcomes.

Despite these limitations, this study offers important contributions to the emerging evidence on the use of clinical cascades as a more precise and targeted alternative to signal function estimates of emergency readiness. This conclusion adds to mounting concerns regarding the accuracy of the signal functions as an indicator of practical obstetric emergency readiness in the real world. By defining the resources necessary to identify and treat emergencies and then monitor-modify treatment as clinically indicated, the cascades provide a detailed, step-wise analysis of resource availability and a novel set of readiness indicators. First, the cascades show precisely where the drop-off in readiness occurs. Second, the drop-off can be estimated at the stage of care, obstetric emergency, and individual resource level. Third, there is growing, preliminary evidence that the variability in drop-off across stages of care and cascades may provide an indicator of emergency readiness at the system level. Results from this study could inform strategies that optimize emergency commodity provision for Kenyan and Ugandan facilities and strengthen system-level strategies for improving emergency-specific maternal survival globally. While measuring readiness according to the clinical cascades model requires a marginal increase in effort during data collection, this approach provides a more nuanced picture of capacity to manage obstetric emergencies. Further investigation into diverse contexts (e.g., additional countries, regions, levels of care) is warranted and could further support existing calls to shift away from standard readiness metrics (such as the signal

For peer review only

functions and SRI) toward the more nuanced, precise, and practical estimates offered by the clinical cascades.

CONCLUSIONS

In conclusion, emergency care is critical in managing the obstetric complications driving elevated maternal mortality ratios globally – and, particularly, in Sub-Saharan Africa. The prominent role emergency care plays in the obstetric experience mandates an accurate measurement of readiness to handle such complications. This study suggests the need to reconsider the signal functions as the preferred method of measuring readiness, which aligns with findings from previous studies. The clinical cascades provide a more nuanced picture of clinical care that can be used to optimize emergency commodity provision and strengthen system-level strategies for improving emergency-specific maternal survival.

Contributors: All authors listed are responsible for this study and have participated in the concept and design, analysis, and interpretation of data, drafting and revising of the manuscript. DW, EB, AW, and PW conceptualized, secured funding, and oversaw data collection for the parent study. BW and JNC conceptualized the nested analysis. BW analyzed the data and wrote the first draft of the submitted manuscript, with assistance from JNC and JMS. All authors provided feedback and commented on the manuscript.

Acknowledgments:
We thank Damien Scogin for his graphic design and Cecilia Roach for her copy-editing contributions. We appreciate all the facility healthcare workers, leadership and administration who made this work possible. We thank all members of the Preterm Birth Initiative Kenya & Uganda Implementation Research Collaborative, particularly the data teams who helped strengthen and collect data from facility maternity registers for the parent PTBi study. We are grateful to the facility clinicians and study participants for their valuable contribution to the parent PTBi study and by extension this nested analysis. Furthermore, we thank the research teams at UCSF, Makerere University, Kenya Medical Research Institute, and Emory University for their hard work, collaboration, and ongoing commitment to maternal survival.

Funding: Bill & Melinda Gates Foundation (Grant # OPP1107312) funded primary data collection and article processing charges. Emory University Rollins School of Public Health's Global Field Experience Financial Award funded effort for the literature review.

Competing interests: AW represents his university on the Sexual, Reproductive, and Child Health Committee for the Bill & Melinda Gates Foundation. JNC has grants from UNICEF and is an Advisory Board Member for Towards Unity in Health and Chair for the Community-Based Primary Health Care working group with the American Public Health Association, International Health Section. DW is on the Board of Director of PRONTO International, a not-for-profit agency. The other authors declare no competing interests.

Data availability statement: Data are available upon reasonable request. The deidentified facility-level data underlying this nested analysis and the results presented in this Article are available to researchers who present ethically sound proposals to DW, the principal investigator of the parent study. Requesters will need to sign a data access agreement that is subject to approval by the parent study principal investigators.

REFERENCES

For peer review only

1 World Health Organization. Maternal mortality, 2019. Available: https://www.who.int/news-room/fact-sheets/detail/maternal-mortality [Accessed Mar 21 2021].

2 World Health Organization. Trends in maternal mortality 2000 to 2017: estimates by WHO, UNICEF, UNFPA, World Bank Group, and the United Nations Population Division. Geneva: Wrold Health Organization; 2019.

3 Say L, Chou D, Gemmill A, et al. Global causes of maternal death: a WHO systematic analysis. *Lancet Glob Health*. 2014;2:e323-33. doi:10.1016/S2214-109X(14)70227-X

4 World Health Organization. Mother-baby package: implementing safe motherhood in countries. Geneva, Switzerland: Maternal Health and Safe Motherhood Programme, Division of Family Health, 1996.

5 World Health Organization. Monitoring emergency obstetric care: a handbook. Geneva, Switzerland: Department of Reproductive Health and Research, 2009.

6 Nesbitt RC, Lohela TJ, Manu A, et al. Correction: quality along the continuum: a health facility assessment of intrapartum and postnatal care in Ghana. *PLoS One*. 2015;10:e0141517. doi:10.1371/journal.pone.0141517

7 Paxton A, Bailey P, Lobis S. The United Nations process indicators for emergency obstetric care: reflections based on a decade of experience. *Obstet Gynecol Int J*. 2006;95:192-208. doi:10.1016/j.ijgo.2006.08.009

8 World Health Organization. Monitoring the building blocks of health systems: a handbook of indicators and their measurement strategies. Geneva, Switzerland: World Health Organization, 2010.

9 Gabrysch S, Civitelli G, Edmond KM, et al. New signal functions to measure the ability of health facilities to provide routine and emergency newborn care. *PLoS Med*. 2012;9:e1001340. doi:10.1371/journal.pmed.1001340

10 Nickerson JW, Adams O, Attaran A, et al. Monitoring the ability to deliver care in low- and middle-income countries: a systematic review of health facility assessment tools. *Health Policy Plan.* 2015;30:675-86. doi:10.1093/heapol/czu043

11 Gill Z, Bailey P, Waxman R, et al. A tool for assessing 'readiness' in emergency obstetric care: The room-by-room 'walk-through'. *Obstet Gynecol Int J*. 2005;89:191-9. doi:10.1016/j.ijgo.2004.12.043

12 Government of Sierra Leone. Sierra Leone service availability and readiness assessment 2011 report. Ministry of Health and Sanitation, 2011.

13 Government of Kenya. Kenya service availability and assessment mapping (SARAM) report 2013. Nairobi, Kenya: Ministry of Health, 2013.

14 O'Neill K, Takane M, Sheffel A, et al. Monitoring service delivery for universal health coverage: the Service Availability and Readiness Assessment. *Bull World Health Organ*. 2013;91:923-31. doi:10.2471/BLT.12.116798

15 World Health Organization. Service availability and readiness assessment (SARA): an annual monitoring system for service delivery. Geneva, Switzerland: World Health Organization, 2015.

16 Spangler SA. Assessing skilled birth attendants and emergency obstetric care in rural Tanzania: the inadequacy of using global standards and indicators to measure local realities. *Reprod Health Matters.* 2012;20:133-41. doi:10.1016/S0968-8080(12)39603-4

17 Brenner S, De Allegri M, Gabrysch S, et al. The quality of clinical maternal and neonatal healthcare – A strategy for identifying 'routine care signal functions'. *PLoS One.* 2015;10:e0123968. doi:10.1371/journal.pone.0123968

18 Bailey P, Paxton A, Lobis S, et al. The availability of life-saving obstetric services in developing countries: An in-depth look at the signal functions for emergency obstetric care. *Obstet Gynecol Int*. 2006;93:285-91. doi:10.1016/j.ijgo.2006.01.028

For peer review only

19 Collender G, Gabrysch S, Campbell OM. Reducing maternal mortality: better monitoring, indicators and benchmarks needed to improve emergency obstetric care. Research summary for policymakers. *Trop Med Int Health*. 2012;17:694-6. doi:10.1111/j.1365-3156.2012.02983.x
20 Gabrysch S, Zanger P, Seneviratne HR, et al. Tracking progress towards safe motherhood: meeting the benchmark yet missing the goal? An appeal for better use of health-system output indicators with evidence from Zambia and Sri Lanka. *Trop Med Int Health*. 2011;16:627-39. doi:10.1111/j.1365-3156.2011.02741.x
21 Cranmer JN, Dettinger J, Calkins K, et al. Beyond signal functions in global obstetric care: Using a clinical cascade to measure emergency obstetric readiness. PLoS One. 2018;13:e0184252. doi:10.1371/journal.pone.0184252
22 Potter C, Brough R. Systemic capacity building: a hierarchy of needs. *Health Policy Plan*. 2004;19:336-45. doi:10.1093/heapol/czh038
23 Gardner EM, McLees MP, Steiner JF, et al. The spectrum of engagement in HIV care and its relevance to test-and-treat strategies for prevention of HIV infection. *Clin Infect Dis*. 2011;52:793-800. doi:10.1093/cid/ciq243
24 Morgan MC, Spindler H, Nambuya H, et al. Clinical cascades as a novel way to assess physical readiness of facilities for the care of small and sick neonates in Kenya and Uganda. *PLoS One*. 2018;13:e0207156. doi:10.1371/journal.pone.0207156
25 Otieno P, Waiswa P, Butrick E, et al. Strengthening intrapartum and immediate newborn care to reduce morbidity and mortality of preterm infants born in health facilities in Migori County, Kenya and Busoga Region, Uganda: a study protocol for a randomized controlled trial. *Trials*. 2018;19:313. doi:10.1186/s13063-018-2696-2
26 The Partnership for Maternal, Newborn & Child Health.. Essential interventions, commodities and guidelines for reproductive, maternal, newborn and child health. Geneva, Switzerland: The Partnership for Maternal, Newborn & Child Health.. Essential interventions, commodities and guidelines for reproductive, maternal, newborn and child health., 2011.
27 World Health Organization. Pregnancy, childbirth, postpartum and newborn care: A guide for essential practice. 3rd ed. Geneva, Switzerland: World Health Organization, 2015.
28 Paxton A, Bailey P, Lobis S, et al. Global patterns in availability of emergency obstetric care. *Int J Gynaecol Obstet*. 2006;93:300-7. doi:10.1016/j.ijgo.2006.01.030
29 Carter R, Xiong X, Lusamba-Dikassa PS, et al. Facility conditions, obstetric and neonatal care practices, and availability of emergency obstetric and neonatal care in 72 rural health facilities in the Democratic Republic of the Congo: A cross-sectional study. *Gates Open Res*. 2019;3:13. doi:10.12688/gatesopenres.12905.2

Figure 1. Emergency readiness estimates by emergency cascade and stage of care

For peer review only

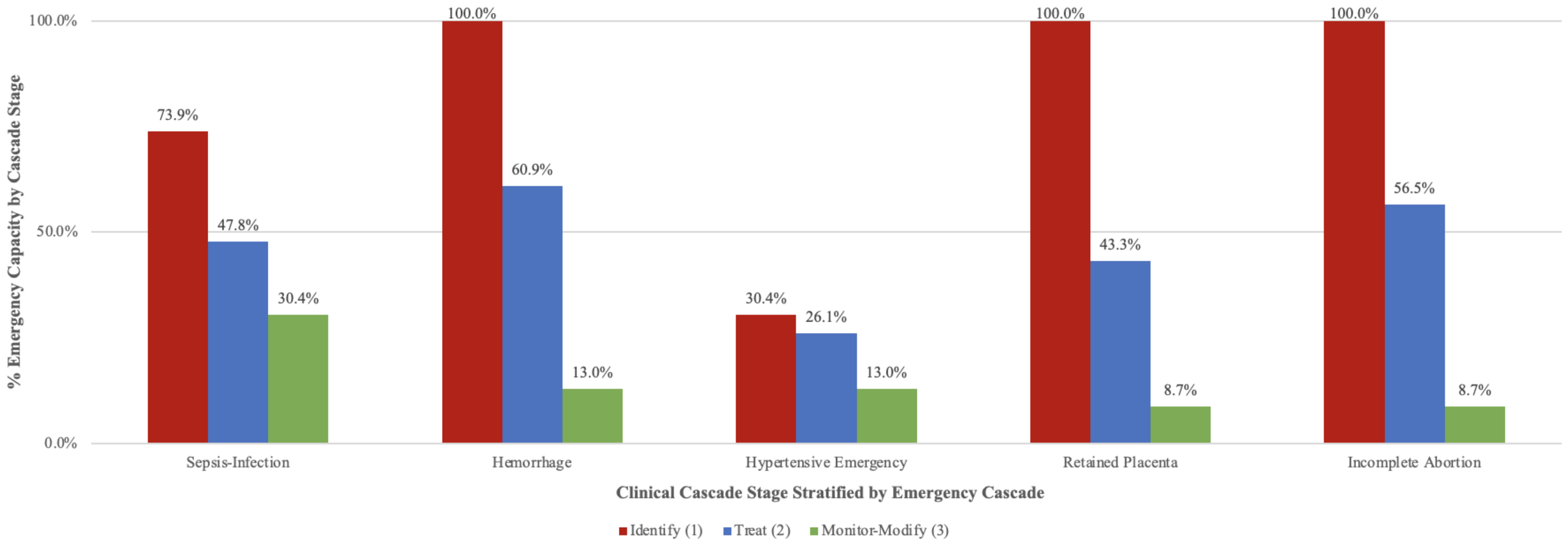
100.0%
50.0%
0.0%
% Emergency Capacity by Cascade Stage
73.9%
47.8%
30.4%
100.0%
60.9%
13.0%
30.4%
26.1%
13.0%
100.0%
43.3%
8.7%
100.0%
56.5%
8.7%
Sepsis-Infection
Hemorrhage
Hypertensive Emergency
Retained Placenta
Incomplete Abortion
Clinical Cascade Stage Stratified by Emergency Cascade
Identify (1)
Treat (2)
Monitor-Modify (3)

SUPPLEMENTAL MATERIAL

Definition of Terms

- **Maternal deaths:** The death of a woman during pregnancy or within 42 days of termination of the pregnancy, regardless of the duration and site of the pregnancy, from any cause related to or exacerbated by the pregnancy or its management, excluding accidental or incidental causes[1]
- **Live birth:** The complete expulsion or extraction of a product of conception from its mother, irrespective of pregnancy duration, which, after separation, breathes or shows other signs of life[1]
- **Maternal Mortality Ratio (MMR):** Maternal deaths per 100,000 live births[1]
- **Hemorrhage:** Severe bleeding before (antepartum hemorrhage) or after (postpartum hemorrhage) labor[2]
- **Sepsis:** A complication caused by infection, resulting in high temperatures (38° C or higher) and one or more of the following symptoms: lower abdominal pain, abnormal vaginal discharge, and tender or poorly contracted uterus[2]
- **Preeclampsia:** A sudden spike in maternal blood pressure accompanied by protein in the urine, which can progress to a more serious eclampsia[2]
- **Eclampsia:** A blood-pressure related complication that can result in maternal seizures which, untreated, can progress to coma or death[2]
- **Prolonged or obstructed labor:** Occurs when the first stage of labor lasts longer than 12 hours, the second stage of labor lasts longer than one hour, or labor is obstructed by uterine scarring or fetal malpresentation (fetus side (transverse), brow, or face presenting in the uterine isthmus instead of the head)[2]
- **Abortion:** The termination of a pregnancy through the removal of pregnancy tissue, products of conception, or the fetus and placenta from the uterus[3]. Complications of a spontaneous or induced abortion, especially an induced abortion performed in non-medical/unsterile settings, can result in hemorrhage, sepsis, or other complications[2]
- **Signal functions:** Six clinical actions, three medical and three manual procedures, used during obstetric emergencies[2]
- **Clinical cascades:** A clinically-oriented approach to measuring facility readiness using a step-wise cascading relationship between emergency resources[4,5]
- **Emergency Obstetric Care (EmOC):** Clinical processes needed to rapidly manage or stabilize a patient whose life is in imminent danger during pregnancy[6]
- **Basic Emergency Obstetric Care (BEmOC):** The ability to perform six maternal signal functions: (1) administer parenteral antibiotics, (2) administer uterotonic drugs, (3) administer parenteral anticonvulsants for pre-eclampsia and eclampsia, (4) manually remove the placenta, (5) remove retained products of conception, and (6) perform assisted vaginal delivery[2]
- **Comprehensive Emergency Obstetric Care (CEmOC):** The ability to perform the six basic signal functions as well as surgery and blood transfusion[2]
- **Drop-off in readiness:** The decrease in the percentage of facilities with the resources necessary to manage basic obstetric emergencies.
- **Private facilities:** Facilities across both Kenya and Uganda that are neither owned nor operated by the government.

For peer review only - http://bmjopen.bmj.com/site/about/guidelines.xhtml

Table S1: Items that Define Signal Functions and Clinical Cascades

Obstetric Emergency	Signal Function	Signal Function Tracer Items	Clinical Cascade Items by Stage of Care		
			Stage 1: Identify	Stage 2: Treat	Stage 3: Monitor-Modify
Manage Sepsis-Infection	Administer Parenteral Antibiotics	IV Fluid (1)<br>IV Cannula/Catheter<br>IV Tubing<br>Ampicillin or Penicillin<br>Gentamicin or Cefotaxime or Ceftriaxone	Thermometer<br>Stethoscope<br>Sphygmomanometer	IV Tubing<br>IV Cannula/Catheter<br>IV Fluid (1)<br>IV Pole<br>Ampicillin or Penicillin<br>Gentamicin or Cefotaxime or Ceftriaxone	Protocol: Sepsis (2)
Manage Hemorrhage	Administer Parenteral Uterotonics	Gloves (3)<br>IV Fluid (1)<br>IV Cannula/Catheter<br>IV Tubing<br>Oxytocin or Misoprostol or Ergometrine	Staff skill (4)	Gloves (3)<br>IV Tubing<br>IV Cannula/Catheter<br>IV Fluid (1)<br>IV Pole<br>Refrigeration (5)<br>Oxytocin	Sphygmomanometer<br>Stethoscope<br>Misoprostol or Ergometrine<br>Urinary Catheter<br>Oxygen Source (6)<br>Oxygen Tubing<br>Oxygen Delivery (7)<br>Protocol: Hemorrhage
Manage Hypertensive Emergencies	Administer Parenteral Anticonvulsant	IV Fluid (1)<br>IV Cannula/Catheter<br>IV Tubing	Sphygmomanometer<br>Stethoscope<br>Urine Cup	IV Tubing<br>IV Cannula/Catheter<br>IV Fluid (1)	Urinary Catheter<br>Calcium Gluconate<br>Oxygen Source (6)

For peer review only

		Magnesium Sulfate	Urine Dipstick	IV Pole<br><br>Magnesium Sulfate<br>Hydralazine or Labetalol or Nifedipine or Methyldopa	Oxygen Tubing<br>Oxygen Delivery [7]<br>Protocol: Eclampsia
Manage Retained Placenta	Perform Manual Removal of Retained Placenta	Flashlight or power<br>IV Fluid [1]<br>IV Cannula/Catheter<br>IV Tubing<br><br>Oxytocin<br>Ampicillin or Penicillin or Gentamicin or Cefotaxime or Ceftriaxone	Staff Skill [4]<br>Light Source [8]	Gloves [3]<br>IV Tubing<br>IV Cannula/Catheter<br>IV Fluid [1]<br><br>IV Pole<br>Refrigeration [5]<br><br>Oxytocin<br>Diazepam<br>Ampicillin or Penicillin	Sphygmomanometer<br>Stethoscope<br>Misoprostol or Ergometrine<br>Gentamicin or Cefotaxime or Ceftriaxone<br>Urinary Catheter<br>Protocol(s) [9]
Manage Incomplete Abortion	Perform Manual Removal of Retained Products of Conception	Flashlight or power<br><br>MVA Kit<br>IV Fluid [1]<br>IV Cannula/Catheter<br>IV Tubing	Speculum<br>Light Source [8]	Gloves [3]<br>IV Tubing<br>IV Cannula/Catheter<br>IV Fluid [1]<br>Manual Vacuum Aspirator Kit [10]	Sphygmomanometer<br>Stethoscope<br>Refrigeration [5]<br>Misoprostol or Ergometrine<br>Gentamicin or Cefotaxime or Ceftriaxone

For peer review only

		Ampicillin or Penicillin or Gentamicin or Cefotaxime or Ceftriaxone		IV Pole<br><br>Lidocaine<br>Ampicillin or Penicillin	Urinary Catheter<br><br>Protocol(s) (11)

(1) Either normal saline (NS) or lactated ringer's (LR)

(2) Puerperal sepsis, infection, or antibiotic administration Protocol

(3) Sterile gloves or clean disposable latex gloves (represented as a single variable in the original dataset)

(4) 100% staff skill for identifying the emergency was assumed

(5) Power used as proxy for refrigeration

(6) Oxygen concentrator and power or oxygen cylinder and oxygen head

(7) Oxygen mask or nasal cannula

(8) Flashlight or power (as proxy for electric lights)

(9) Retained placenta protocol or hemorrhage protocol or infection protocol

(10) Manual vacuum device and cannula

(11) Incomplete abortion protocol or hemorrhage protocol or infection protocol

For peer review only

Table S2. Facility Characteristics

	**%**	**n** [(1)]
**Country**		
Kenya	73.9%	17
Uganda	26.1%	6
**Level (ownership)**		
Private	17.4%	4
Government	82.6%	19
Regional Referral Hospital	*4.4%*	*1*
District Hospital	*13.0%*	*3*
County Referral Hospital	*4.4%*	*1*
Sub-County Hospital	*43.5%*	*10*
Health Center	*17.4%*	*4*
**Reported C-Section Capability**		
Yes	43.5%	10
No	56.5%	13

[(1)] n = 23

For peer review only

Table S3. Annual Delivery Volume

	n	Annual Total [1]	Median [2]	25th	75th	IQR
**Kenya**	17	15,858.5	598.9	495.3	999.3	504.0
**Uganda**	6	21,591.3	2,662.5	2,059.3	5,396.0	3,336.7
Total	23	*37,449.8*	*994.5*	*537.3*	*1,808.6*	*1,271.4*

[1] Data was provided for up to 11 months in Kenya and up to 12 months in Uganda. To calculate the total annual delivery volume, we calculate the monthly average using the number of reported months for each individual facility and then multiplied that number by 12.
[2] The median and IQR are reported instead of the mean and standard deviation as the data are not normally distributed.

For peer review only

Table S4. Comparison of Emergency Readiness Using Clinical Cascades and Signal Functions by Reported C-Section Capability

	Signal Functions		Clinical Cascades		Overestimated Readiness	
	**% Readiness, Tracer Items**		**% Readiness, Stage 2**		**Percentage Point Difference [Signal Functions (-) Cascade]**	
**Clinical Cascade** *(Signal Function)*	**C-section** (1)	**No C-section** (2)	**C-section**	**No C-section**	**C-section**	**No C-section**
**Medical Treatments**						
**Manage Sepsis - Infection** *(Antibiotic)*	90.0%	53.9%	50.0%	46.2%	40.0	7.7
**Manage Hemorrhage** *(Uterotonics)*	70.0%	53.9%	70.0%	53.9%	0.0	0.0
**Manage Hypertensive Emergency** *(Anticonvulsant)*	90.0%	69.2%	30.0%	23.1%	60.0	46.2
Medical Readiness, Overall Mean (pooled)	83.3%	59.0%	50.0%	41.0%	33.3	18.0
**Manual Procedures**						
**Manage Retained Placenta** *(Removal of retained placenta)*	70.0%	69.2%	70.0%	23.1%	0.0	46.2
**Manage Incomplete Abortion** *(Removal of retained products of conception)*	80.0%	61.5%	70.0%	46.2%	10.0	15.4
Manual Readiness, Overall Mean (pooled)	75.0%	65.4%	70.0%	34.6%	5.0	30.8
**Overall Mean Readiness (pooled)**	**80.0%**	**61.5%**	**58.0%**	**38.5%**	**22.0**	**23.1**
	Signal Function Estimate		*Cascade Estimate*		*Percentage Point Overestimation by Signal Functions*	

(1) n = 10 facilities
(2) n = 13 facilities

For peer review only

Table S5. Comparison of Emergency Readiness Using Clinical Cascades and Signal Functions by Ownership

	Signal Functions		Clinical Cascades		Overestimated Readiness	
	% Readiness, Tracer Items		% Readiness, Stage 2		Percentage Point Difference [Signal Functions (-) Cascade]	
**Clinical Cascade** *(Signal Function)*	**Gov.** [(1)]	**Private** [(2)]	**Gov.**	**Private**	**Government**	**Private**
**Medical Treatments**						
**Manage Sepsis - Infection** *(Antibiotic)*	63.2%	100.0%	42.1%	75.0%	21.1	25.0
**Manage Hemorrhage** *(Uterotonics)*	57.9%	100.0%	57.9%	100.0%	0.0	0.0
**Manage Hypertensive Emergency** *(Anticonvulsant)*	73.7%	100.0%	26.3%	25.0%	47.4	75.0
Medical Readiness, Overall Mean (pooled)	64.9%	100.0%	42.1%	66.7%	22.8	33.3
**Manual Procedures**						
**Manage Retained Placenta** *(Removal of retained placenta)*	68.4%	100.0%	36.8%	100.0%	31.6	0.0
**Manage Incomplete Abortion** *(Removal of retained products of conception)*	63.2%	75.0%	52.6%	75.0%	10.5	0.0
Manual Readiness, Overall Mean (pooled)	65.8%	87.5%	44.7%	87.5%	21.1	0.0
**Overall Mean Readiness (pooled)**	**65.3%**	**95.0%**	**43.2%**	**75.0%**	**22.1**	**20.0**
	Signal Function Estimate		*Cascade Estimate*		*Percentage Point Overestimation by Signal Functions*	

[(1)] n = 19 facilities
[(2)] n = 4 facilities

For peer review only

Table S6. Comparison of Emergency Readiness Using Clinical Cascades and Signal Functions by Country

	Signal Functions		Clinical Cascades		Overestimated Readiness	
	% Readiness, Tracer Items		% Readiness, Stage 2		Percentage Point Difference[Signal Functions (-) Cascade]	
**Clinical Cascade** *(Signal Function)*	**Kenya**[(1)]	**Uganda**[(2)]	**Kenya**	**Uganda**	**Kenya**	**Uganda**
**Medical Treatments**						
**Manage Sepsis – Infection** *(Antibiotic)*	64.7%	83.3%	52.9%	33.3%	11.8	50.0
**Manage Hemorrhage** *(Uterotonics)*	58.8%	66.7%	58.8%	66.7%	0.0	0.0
**Manage Hypertensive Emergency** *(Anticonvulsant)*	76.5%	83.3%	23.5%	33.3%	52.9	50.0
Medical Readiness, Overall Mean (pooled)	66.7%	77.8%	45.1%	44.4%	21.6	33.3
**Manual Procedures**						
**Manage Retained Placenta** *(Removal of retained placenta)*	70.6%	66.7%	35.3%	66.7%	35.3	0.0
**Manage Incomplete Abortion** *(Removal of retained products of conception)*	70.6%	66.7%	58.8%	50.0%	11.8	16.7
Manual Readiness, Overall Mean (pooled0	70.6%	66.7%	47.0%	58.3%	23.5	8.3
**Overall Mean Readiness (pooled)**	**68.2%**	**73.3%**	**45.9%**	**50.0%**	**22.4**	**23.3**
	Signal Function Estimate		*Cascade Estimate*		*Percentage Point Overestimation by Signal Functions*	

(1) n = 17 facilities
(2) n = 6 facilities

For peer review only

Table S7. Emergency Readiness by Clinical Cascade

Clinical Cascade *(Signal Function)*	Cascade Stage	Item	%	n [1]
Manage Sepsis-Infection *(Antibiotic)*	Identify	Thermometer	95.7	22
		Stethoscope	78.3	18
		Sphygmomanometer	73.9	17
	Treat (Consumables)	IV Tubing	60.9	14
		IV Cannula-Catheter	52.2	12
		IV Fluid [2]	52.2	12
	Treat (Durables)	IV Pole	52.2	12
	Treat (Treatments)	Parenteral Antibiotic-Step 1 [3]	52.2	12
		Parenteral Antibiotic-Step 2 [4]	47.8	7
	Monitor-Modify	Protocol: Sepsis [5]	30.4	1
Manage Hemorrhage (*Uterotonic*)	Identify	Staff skill [6]	100.0	23
	Treat (Consumables)	Gloves, aseptic [7]	78.3	18
		IV Tubing	69.6	16
		IV Cannula-Catheter	65.2	15
		IV Fluid	65.2	15
	Treat (Durables)	IV Pole	65.2	15
		Refrigeration [8]	65.2	15
	Treat (Treatments)	Parenteral Uterotonic [9]	60.9	14
	Monitor-Modify	Sphygmomanometer	52.2	12
		Stethoscope	52.2	12
		Uterotonic, Non-oxytocin [10]	21.7	5
		Urinary catheter	21.7	5
		Oxygen source [11]	21.7	5
		Oxygen tubing	17.4	4
		Oxygen delivery [12]	17.4	4
		Protocol: Hemorrhage	13.0	3
Manage Hypertensive Emergencies (*Anticonvulsant*)	Identify	Sphygmomanometer	91.3	21
		Stethoscope	78.3	18
		Urine Cup	43.5	10
		Urine Dipstick	30.4	7
	Treat (Consumables)	IV Tubing	30.4	7

For peer review only

		IV Cannula-Catheter	30.4	6
		IV Fluid	26.1	6
	Treat (Durables)	IV Pole	26.1	6
	Treat (Treatments)	Parenteral Anticonvulsant [13]	26.1	6
		Parenteral Antihypertensive [14]	26.1	6
	Monitor-Modify	Urinary catheter	26.1	6
		Calcium Gluconate	17.4	4
		Oxygen source [11]	17.4	4
		Oxygen tubing	13.0	3
		Oxygen delivery [12]	13.0	3
		Protocol: Eclampsia	13.0	3

Manage Retained Placenta *(Manual removal of retained placenta)*	Identify	Staff Skill [6]	100.0	23
		Light Source [15]	100.0	23
	Treat (Consumables)	Gloves, Aseptic [7]	78.3	18
		IV Tubing	69.6	16
		IV Cannula-Catheter	65.2	15
		IV Fluid	65.2	15
	Treat (Durables)	IV Pole	65.2	15
		Refrigeration [8]	65.2	15
	Treat (Treatments)	Parenteral Uterotonic (Oxytocin)	60.9	14
		Parenteral Sedative (Diazepam)	43.5	10
		Parenteral Antibiotic-Step 1 [3]	43.5	10
	Monitor-Modify	Sphygmomanometer	34.8	8
		Stethoscope	34.8	8
		Uterotonic, non-oxytocin [10]	17.4	4
		Parenteral Antibiotic-Step 2 [4]	13.0	3
		Urinary catheter	13.0	3
		Protocols [16]	8.7	2
Manage Incomplete Abortion *(Manual removal of retained products of conception)*	Identify	Speculum	100.0	23
		Light Source [15]	100.0	23
	Treat (Consumables)	Gloves, Sterile [7]	78.3	18
		IV Tubing	69.6	16
		IV Cannula-Catheter	65.2	15
		IV Fluid	65.2	15

For peer review only

	Treat (Durables)	Manual Vacuum Aspirator Kit [17]	56.5	13
		IV Pole	56.5	13
	Treat (Treatments)	Local Anesthetic (Lidocaine)	56.5	13
		Parenteral Antibiotic-Step 1 [3]	56.5	13
	Monitor-Modify	Sphygmomanometer	52.2	12
		Stethoscope	47.8	11
		Refrigeration [8]	47.8	11
		Uterotonic, non-oxytocin [10]	17.4	4
		Parenteral Antibiotic-Step 2 [4]	13.0	3
		Catheter, Urinary	13.0	3
		Protocols [18]	8.7	2

(1) Total sample n=23 facilities
(2) Normal saline (NS) or Lactated Ringer's (LR)
(3) Parenteral ampicillin or parenteral penicillin
(4) Parenteral gentamycin or cefotaxime/ceftriaxone
(5) Puerperal sepsis, infection or antibiotic administration protocol
(6) 100% staff skill for identifying the emergency was assumed
(7) Sterile gloves or aseptic disposable gloves were reported as a single item in the dataset
(8) Power used as a proxy for refrigeration
(9) Oxytocin (as tranexamic acid and carbetocene were not available in the dataset
(10) Misoprostol tablets or ergometrine (IM)
(11) Oxygen concentrator plus power or oxygen cylinder and oxygen head
(12) Oxygen mask or nasal cannula
(13) Magnesium sulfate IV
(14) Hydralazine or Labetalol or Nifedipine or Methyldopa
(15) Flashlight or power (as proxy for electric lights
(16) Protocol for retained placenta or hemorrhage or infection
(17) Manual vacuum aspirator device and MVA cannula
(18) Protocol for incomplete abortion, hemorrhage or infection

For peer review only

Table S8. Mean Drop-Off by Cascade and Stage of Care by Reported C-Section Capability

Readiness Drop-Off by Stage of Care							Readiness Drop-Off by Emergency			
**Clinical Cascade**	**1**		**2**		**3**		***Mean Drop-Off Across 3 Cascade Stages of Care***		***SD***	
	**Identify**		**Treat**		**Monitor-Modify**					
**Reported C-Section Capability**	**C-section** [1]	**No C-section** [2]	**C-section**	**No C-section**	**C-section**	**No C-section**	**C-section**	**No C-section**	**C-section**	**No C-section**
	--		--		--		**26.7%**[(3)]	**29.7%**	24.8[(4)]	29.5
**Sepsis-Infection**	40.0%	15.4%	10.0%	38.5%	10.0%	23.1%	20.0%	25.6%	17.3	11.8
**Hemorrhage**	0.0%	0.0%	30.0%	46.2%	60.0%	38.5%	30.0%	28.2%	30.0	24.7
**Hypertensive Emergency**	60.0%	76.9%	10.0%	0.0%	20.0%	7.7%	30.0%	28.2%	26.5	42.4
**Retained Placenta**	0.0%	0.0%	30.0%	76.9%	50.0%	23.1%	26.7%	33.3%	25.2	39.5
**Incomplete Abortion**	0.0%	0.0%	30.0%	53.9%	50.0%	46.2%	26.7%	33.3%	25.2	29.1
**Overall Drop-Off by Stage of Care**										
Drop-Off by Stage of Care (Across All Emergencies), Pooled Mean	**20.0%**	**18.5%**	**22.0%**	**43.1%**	**38.0%**	**27.7%**				
SD	28.3	33.4	11.0	28.1	21.7	15.0	4.1[(5)]	3.4		

(1) n = 10 facilities
(2) n = 13 facilities
(3) Pooled mean readiness drop-off across 3 clinical cascade stages of care and 5 emergencies
(4) Mean of the standard deviations
(5) Standard deviation across 3 stages of care and 5 emergency cascades

For peer review only

Table S9. Mean Drop-Off in Readiness by Cascade and Stage of Care by Ownership

**Readiness Drop-Off by Stage of Care**							**Readiness Drop-Off by Emergency**			
**Clinical Cascade**	**1**		**2**		**3**		***Mean Drop-Off Across 3 Cascade Stages of Care***		***SD***	
	**Identify**		**Treat**		**Monitor-Modify**					
**Ownership**	**Gov.** (1)	**Private** (2)	**Gov.**	**Private**	**Gov.**	**Private**	**Gov.**	**Private**	**Gov.**	**Private**
	--		--		--		**29.1%**[3]	**25.0%**	**24.7**[4]	**35.7**
**Sepsis-Infection**	26.3%	25.0%	31.6%	0.0%	21.1%	0.0%	26.3%	8.3%	5.3	14.4
**Hemorrhage**	0.0%	0.0%	42.1%	0.0%	42.1%	100.0%	28.1%	33.3%	24.3	57.7
**Hypertensive Emergency**	68.4%	75.0%	5.3%	0.0%	10.5%	25.0%	28.1%	33.3%	35.0	38.2
**Retained Placenta**	0.0%	0.0%	63.2%	0.0%	31.6%	75.0%	31.6%	25.0%	31.6	43.3
**Incomplete Abortion**	0.0%	0.0%	47.4%	25.0%	47.4%	50.0%	31.6%	25.0%	27.4	25.0
**Overall Drop-Off by Stage of Care**										
Drop-Off by Stage of Care (Across All Emergencies), Pooled Mean	**19.1%**	**20.0%**	**37.9%**	**5.0%**	**30.5%**	**50.0%**				
SD	**29.9**	**32.6**	**21.5**	**11.2**	**15.1**	**39.5**	**2.4**[5]	**10.2**		

(1) n = 19 facilities
(2) n = 4 facilities
(3) Pooled mean readiness drop-off across 3 clinical cascade stages of care and 5 emergencies
(4) Mean of the standard deviations
(5) Standard deviation across 3 stages of care and 5 emergencies

For peer review only

Table S10. Mean Drop-Off in Readiness by Cascade and Stage of Care by Country

Readiness Drop-Off by Stage of Care							Readiness Drop-Off by Emergency			
**Clinical Cascade**	**1**		**2**		**3**		***Mean Drop-Off Across 3 Cascade Stages of Care***		***SD***	
	**Identify**		**Treat**		**Monitor-Modify**					
**Country**	**Kenya**(1)	**Uganda**(2)	**Kenya**	**Uganda**	**Kenya**	**Uganda**	**Kenya**	**Uganda**	**Kenya**	**Uganda**
	--		--		--		**27.8%**(3)	**30.0%**	26.7(4)	24.0
**Sepsis-Infection**	17.7%	50.0%	29.4%	16.7%	17.7%	16.7%	21.6%	27.8%	6.8	19.3
**Hemorrhage**	0.0%	0.0%	41.2%	33.3%	41.2%	66.7%	27.5%	33.3%	23.8	33.3
**Hypertensive Emergency**	76.5%	50.0%	0.0%	16.7%	5.9%	33.3%	27.5%	33.3%	42.6	16.7
**Retained Placenta**	0.0%	0.0%	64.7%	33.3%	29.4%	50.0%	31.4%	27.8%	32.4	25.5
**Incomplete Abortion**	0.0%	0.0%	41.2%	50.0%	52.9%	33.3%	31.4%	27.8%	27.8	25.5
**Overall Drop-Off by Stage of Care**										
Drop-Off by Stage of Care (Across All Emergencies), Pooled Mean	**18.8%**	**20.0%**	**35.3%**	**30.0%**	**29.4%**	**40.0%**				
SD	**33.1**	**27.4**	**23.5**	**13.9**	**18.6**	**19.0**	**4.0**(5)	**3.0**		

(1) n = 17 facilities

(2) n = 6 facilities

(3) Pooled mean readiness drop-off across 3 clinical cascade stages of care and 5 emergencies

(4) Mean of the standard deviations

(5) Standard deviation across 3 stages of care and 5 emergency cascades

For peer review only

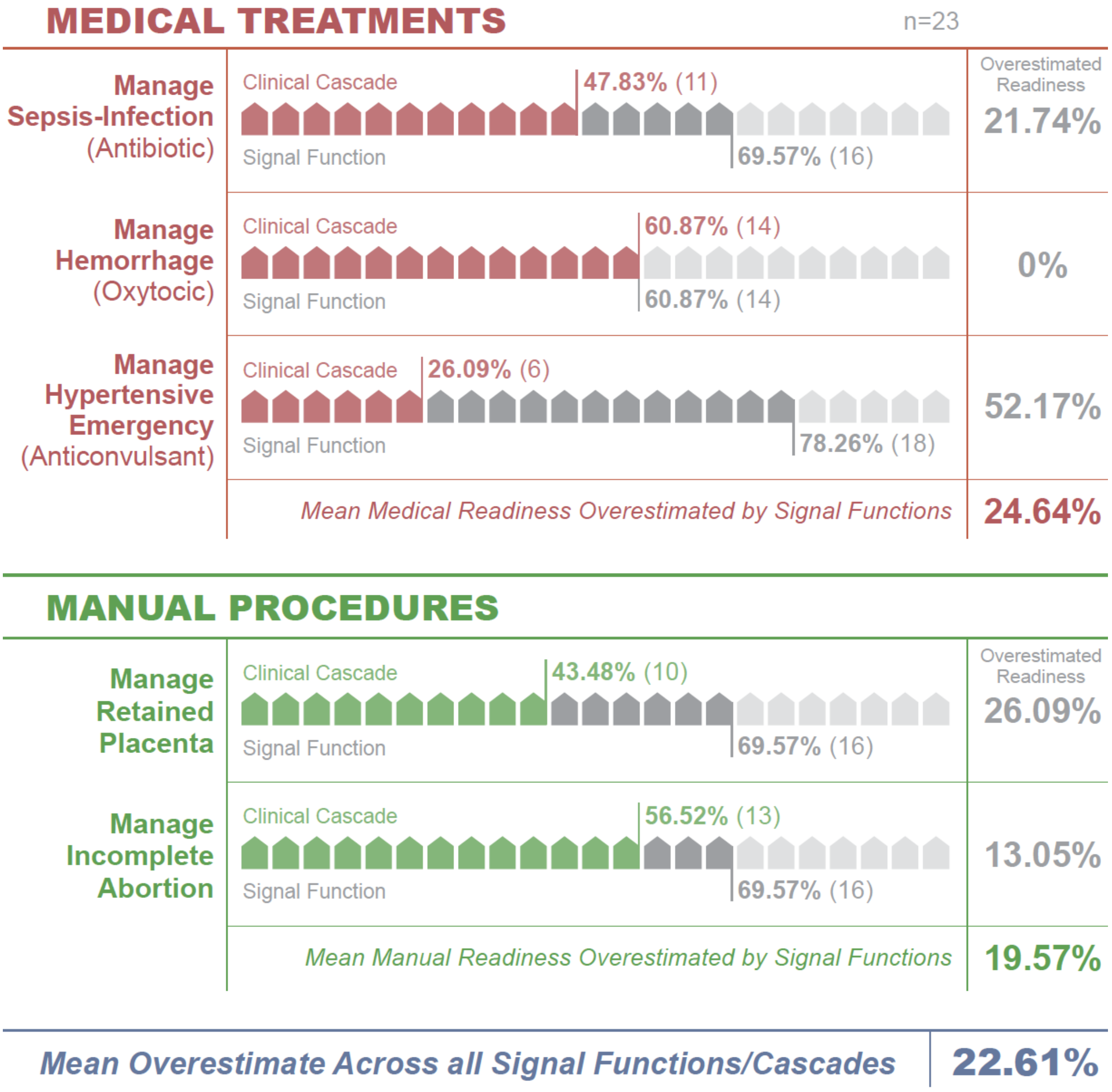

Figure S1. Mean Overestimate Across All Signal Functions and Cascades

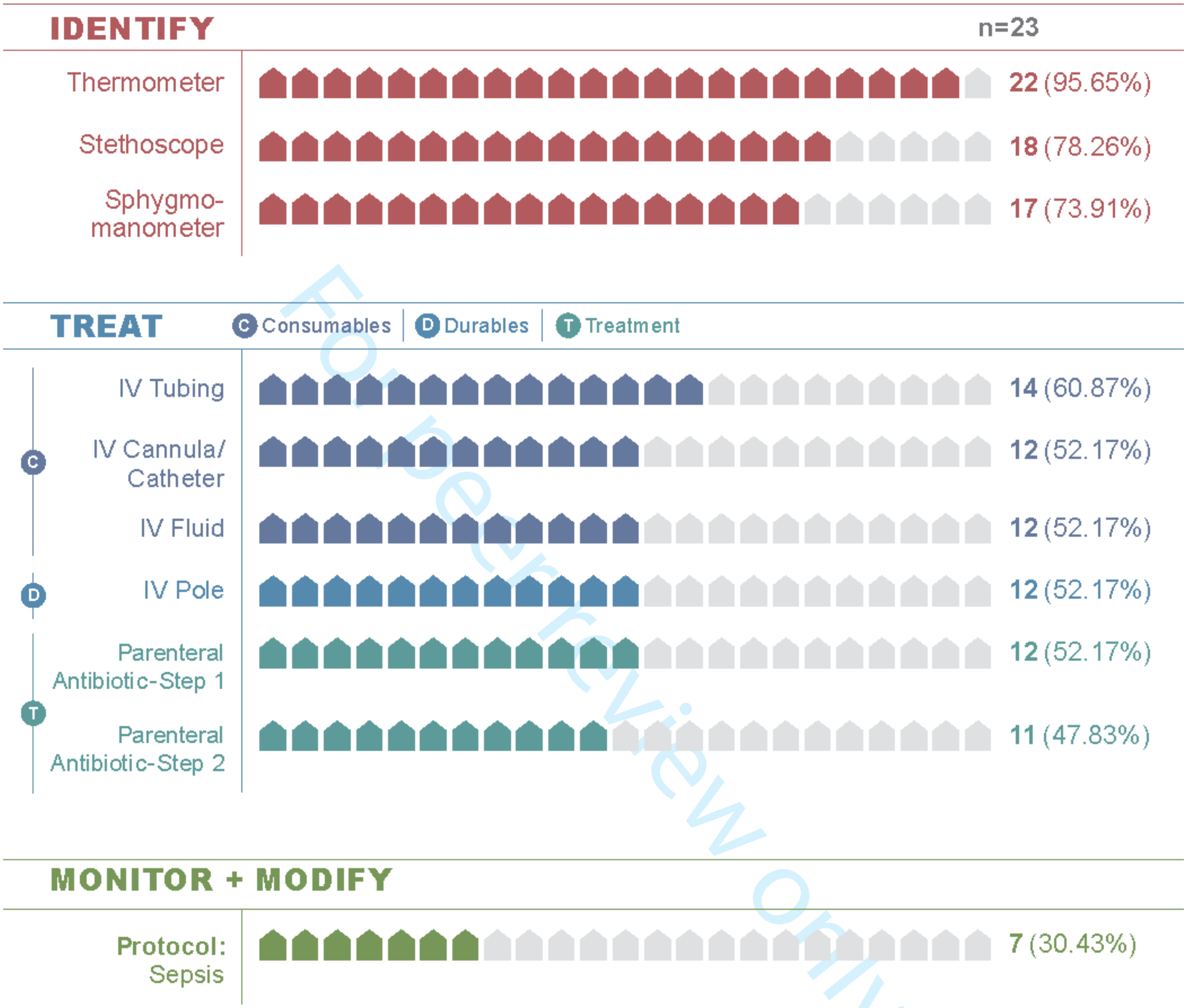

Figure S2. Sepsis Clinical Cascade

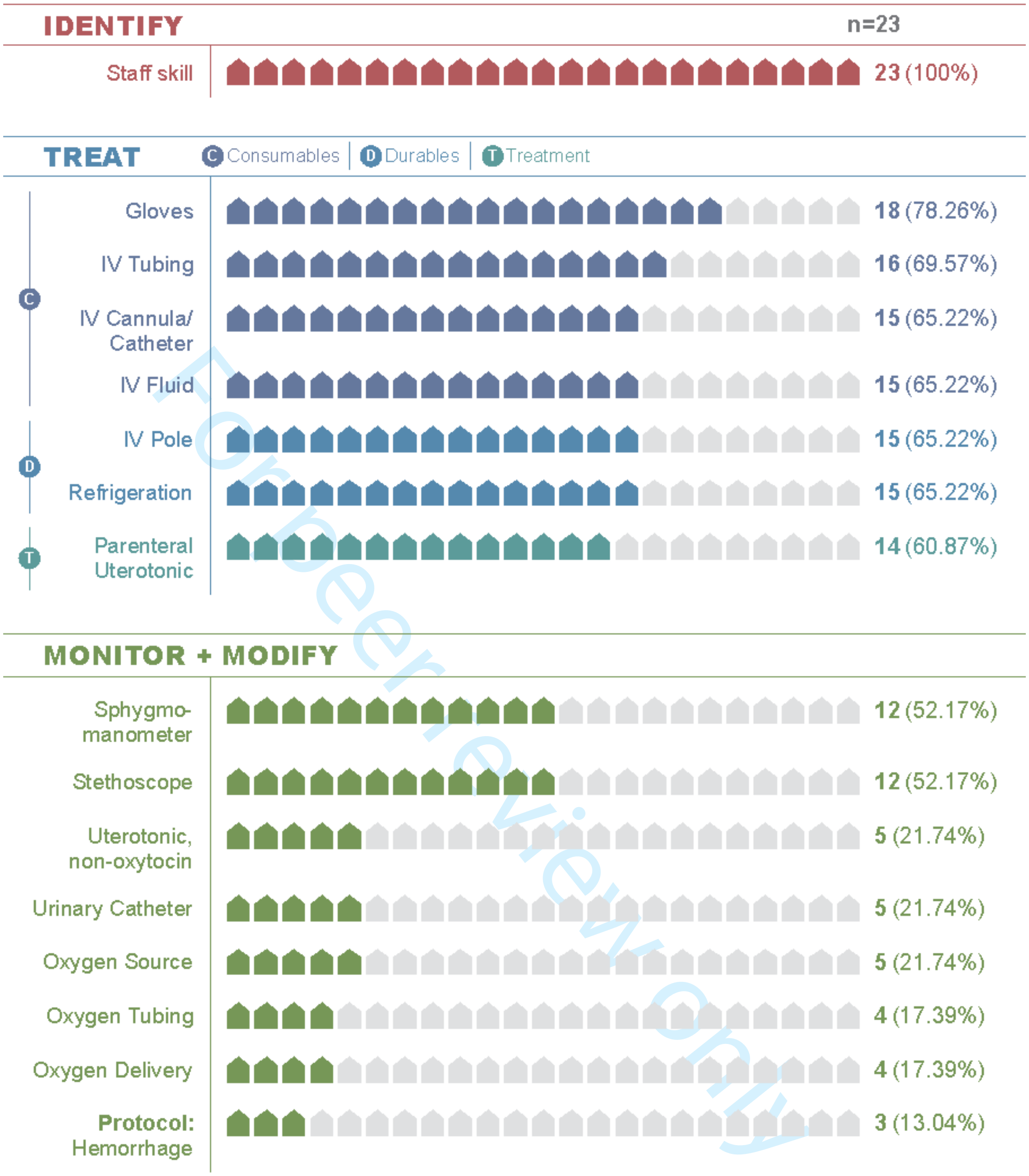

Figure S3. Hemorrhage Clinical Cascade

For peer review only

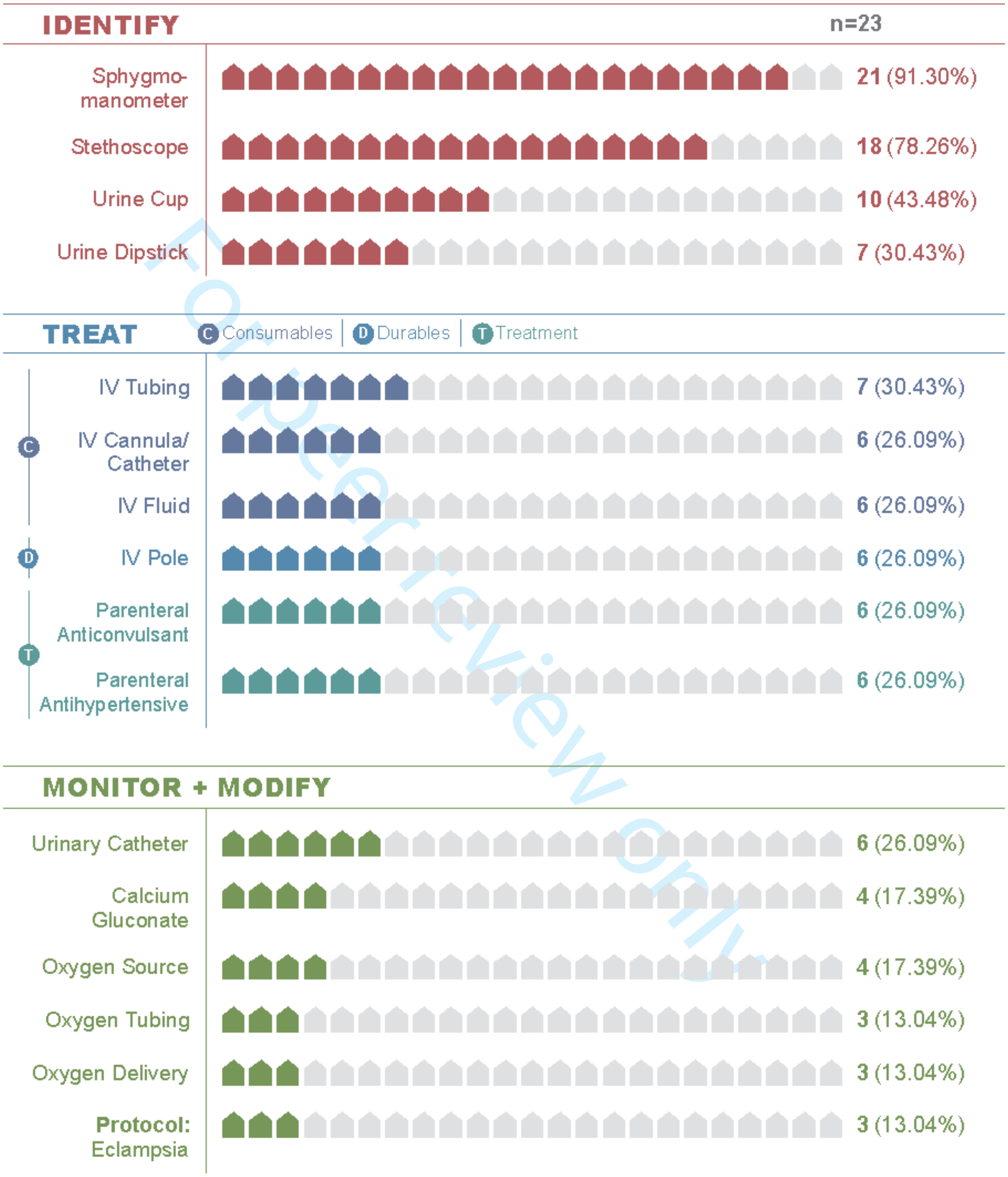

Figure S4. Hypertensive Emergency Clinical Cascade

For peer review only

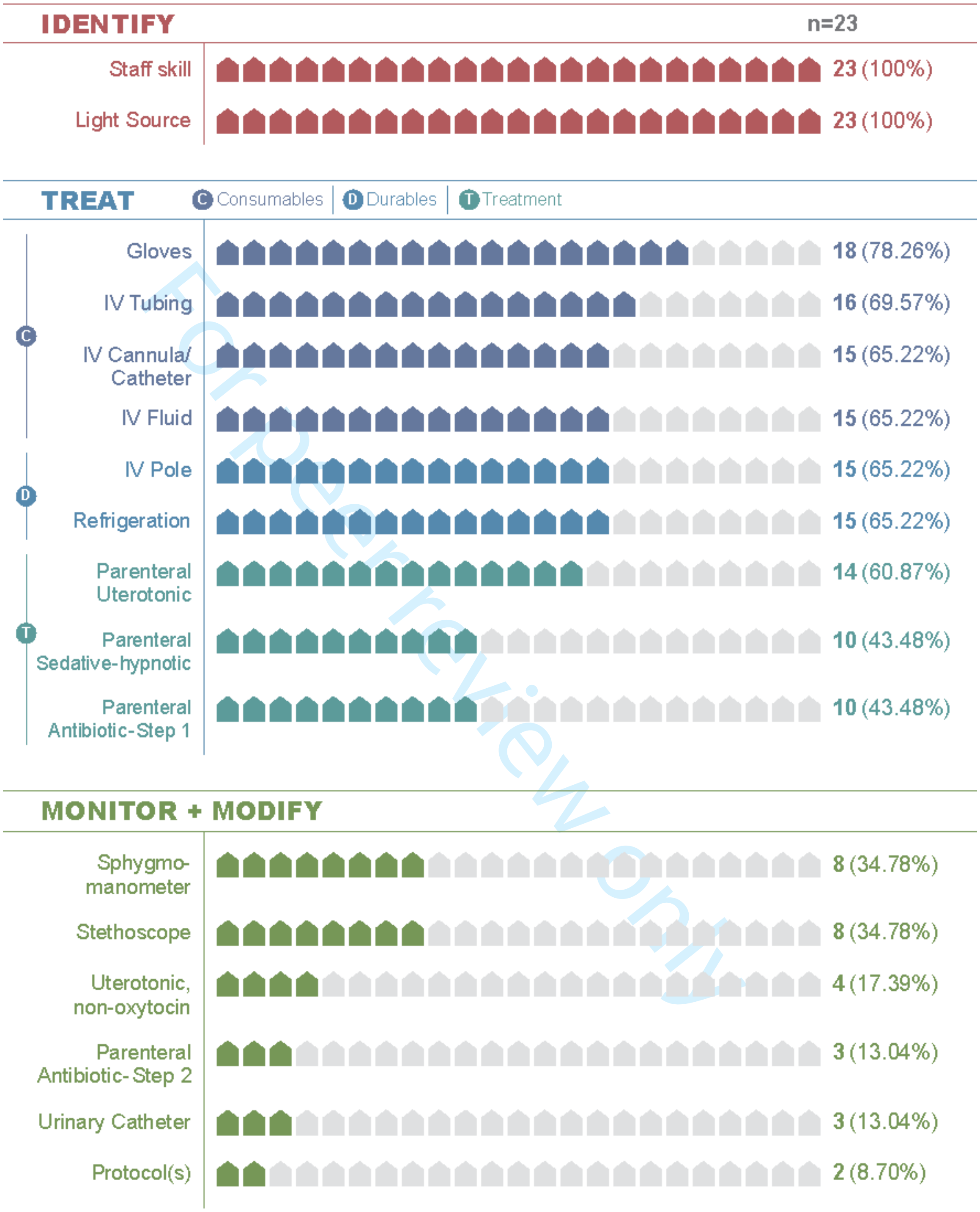

Figure S5. Retained Placenta Clinical Cascade

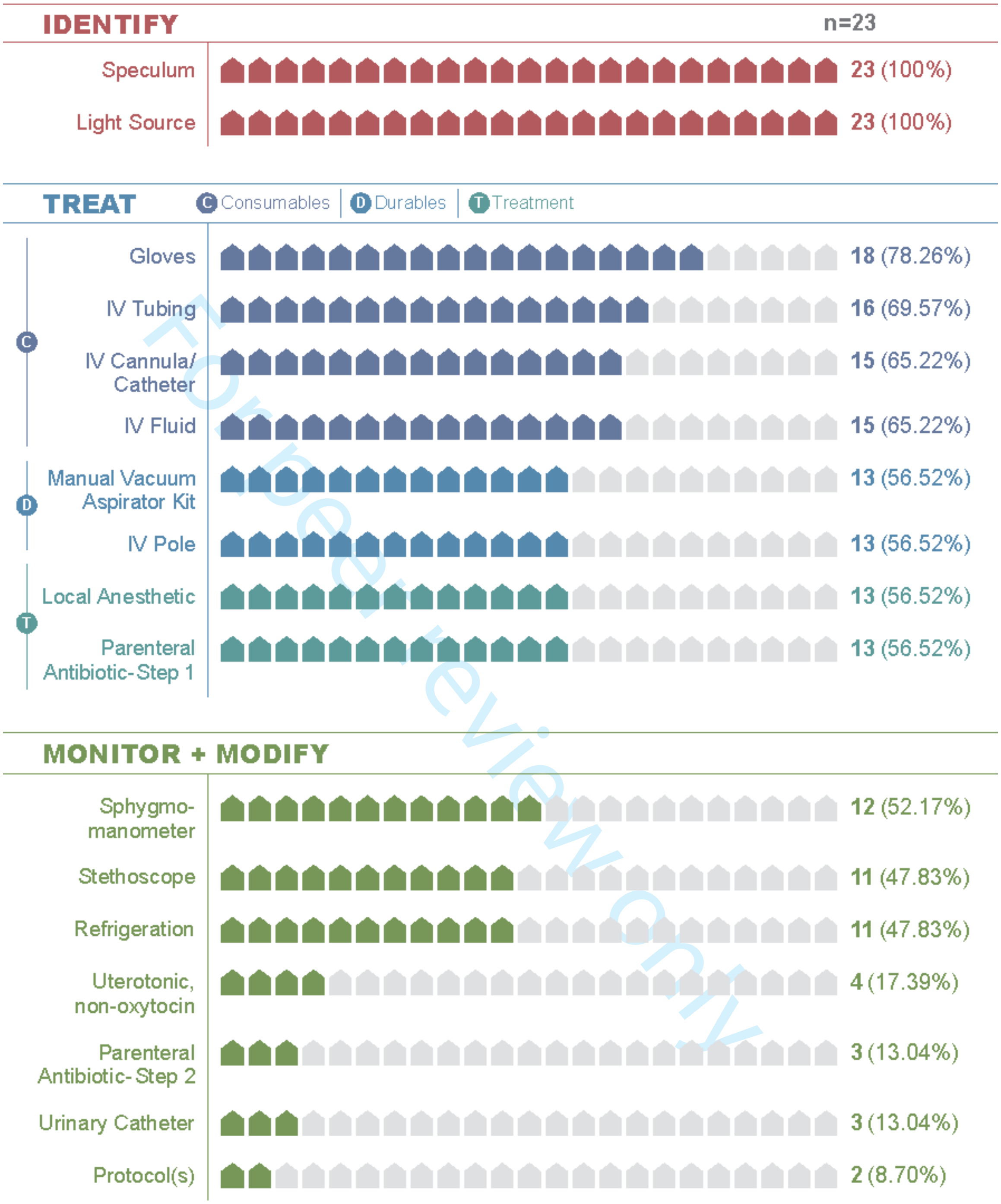

Figure S6. Incomplete Abortion Clinical Cascade

REFERENCES

[1] World Health Organization. Health statistics and information systems: maternal mortality ratio (per 100,000 live births), 2020. Available: https://www.who.int/healthinfo/statistics/indmaternalmortality/en/.

[2] World Health Organization. Monitoring emergency obstetric care: a handbook. Geneva, Switzerland: Department of Reproductive Health and Research, 2009.

[3] Harvard School of Medicine. Abortion (Termination of Pregnancy), 2019. Available: https://www.health.harvard.edu/medical-tests-and-procedures/abortion-termination-of-pregnancy-a-to-z.

[4] Cranmer JN, Dettinger J, Calkins K, et al. Beyond signal functions in global obstetric care: Using a clinical cascade to measure emergency obstetric readiness. PLoS One. 2018;13:e0184252. doi:10.1371/journal.pone.0184252

[5] Morgan MC, Spindler H, Nambuya H, et al. Clinical cascades as a novel way to assess physical readiness of facilities for the care of small and sick neonates in Kenya and Uganda. *PLoS One*. 2018;13:e0207156. doi:10.1371/journal.pone.0207156

[6] Brenner S, De Allegri M, Gabrysch S, et al. The quality of clinical maternal and neonatal healthcare – A strategy for identifying 'routine care signal functions'. *PLoS One*. 2015;10:e0123968. doi:10.1371/journal.pone.0123968

For peer review only

STROBE Statement—Checklist of items that should be included in reports of ***cross-sectional studies***

	Item No	Recommendation	Page No
**Title and abstract**	1	(*a*) Indicate the study's design with a commonly used term in the title or the abstract	1
		(*b*) Provide in the abstract an informative and balanced summary of what was done and what was found	2
**Introduction**			
Background/rationale	2	Explain the scientific background and rationale for the investigation being reported	3-4
Objectives	3	State specific objectives, including any prespecified hypotheses	4
**Methods**			
Study design	4	Present key elements of study design early in the paper	4
Setting	5	Describe the setting, locations, and relevant dates, including periods of recruitment, exposure, follow-up, and data collection	4
Participants	6	(*a*) Give the eligibility criteria, and the sources and methods of selection of participants	
Variables	7	Clearly define all outcomes, exposures, predictors, potential confounders, and effect modifiers. Give diagnostic criteria, if applicable	4-5
Data sources/ measurement	8*	For each variable of interest, give sources of data and details of methods of assessment (measurement). Describe comparability of assessment methods if there is more than one group	*4-5*
Bias	9	Describe any efforts to address potential sources of bias	
Study size	10	Explain how the study size was arrived at	
Quantitative variables	11	Explain how quantitative variables were handled in the analyses. If applicable, describe which groupings were chosen and why	4-6
Statistical methods	12	(*a*) Describe all statistical methods, including those used to control for confounding	6
		(*b*) Describe any methods used to examine subgroups and interactions	6
		(*c*) Explain how missing data were addressed	5
		(*d*) If applicable, describe analytical methods taking account of sampling strategy	
		(*e*) Describe any sensitivity analyses	
**Results**			
Participants	13*	(a) Report numbers of individuals at each stage of study—eg numbers potentially eligible, examined for eligibility, confirmed eligible, included in the study, completing follow-up, and analysed	7
		(b) Give reasons for non-participation at each stage	N/A
		(c) Consider use of a flow diagram	N/A
Descriptive data	14*	(a) Give characteristics of study participants (eg demographic, clinical, social) and information on exposures and potential confounders	7
		(b) Indicate number of participants with missing data for each variable of interest	N/A
Outcome data	15*	Report numbers of outcome events or summary measures	7-13
Main results	16	(*a*) Give unadjusted estimates and, if applicable, confounder-adjusted estimates and their precision (eg, 95% confidence interval). Make clear which confounders were adjusted for and why they were included	7-13

For peer review only - http://bmjopen.bmj.com/site/about/guidelines.xhtml

		(*b*) Report category boundaries when continuous variables were categorized	
		(*c*) If relevant, consider translating estimates of relative risk into absolute risk for a meaningful time period	
Other analyses	17	Report other analyses done—eg analyses of subgroups and interactions, and sensitivity analyses	7-13
**Discussion**			
Key results	18	Summarise key results with reference to study objectives	14-16
Limitations	19	Discuss limitations of the study, taking into account sources of potential bias or imprecision. Discuss both direction and magnitude of any potential bias	14-16
Interpretation	20	Give a cautious overall interpretation of results considering objectives, limitations, multiplicity of analyses, results from similar studies, and other relevant evidence	14-16
Generalisability	21	Discuss the generalisability (external validity) of the study results	14-16
**Other information**			
Funding	22	Give the source of funding and the role of the funders for the present study and, if applicable, for the original study on which the present article is based	16

*Give information separately for exposed and unexposed groups.

Note: An Explanation and Elaboration article discusses each checklist item and gives methodological background and published examples of transparent reporting. The STROBE checklist is best used in conjunction with this article (freely available on the Web sites of PLoS Medicine at http://www.plosmedicine.org/, Annals of Internal Medicine at http://www.annals.org/, and Epidemiology at http://www.epidem.com/). Information on the STROBE Initiative is available at www.strobe-statement.org.

For peer review only

For peer review only - http://bmjopen.bmj.com/site/about/guidelines.xhtml
